# Backbone-controlled LUMO energy induces intramolecular C–H activation in *ortho*-bis-9-borafluorene-substituted phenyl and *o*-carboranyl compounds leading to novel 9,10-diboraanthracene derivatives†

**DOI:** 10.1039/d2sc06057d

**Published:** 2022-11-23

**Authors:** Johannes Krebs, Alena Häfner, Sonja Fuchs, Xueying Guo, Florian Rauch, Antonius Eichhorn, Ivo Krummenacher, Alexandra Friedrich, Lei Ji, Maik Finze, Zhenyang Lin, Holger Braunschweig, Todd B. Marder

**Affiliations:** Institute for Inorganic Chemistry and Institute for Sustainable Chemistry & Catalysis with Boron, Julius-Maximilians-Universität Würzburg Am Hubland 97074 Würzburg Germany h.braunschweig@uni-wuerzburg.de todd.marder@uni-wuerzburg.de; Department of Chemistry, The Hong Kong University of Science and Technology Clear Water Bay Hong Kong chzlin@ust.hk; Frontiers Science Center for Flexible Electronics, Xi'an Institute of Flexible Electronics (IFE), Northwestern Polytechnical University 127 West Youyi Road Xi'an Shaanxi P. R. China iamlji@nwpu.edu.cn

## Abstract

The choice of backbone linker for two *ortho*-bis-(9-borafluorene)s has a great influence on the LUMO located at the boron centers and, therefore, the reactivity of the respective compounds. Herein, we report the room temperature rearrangement of 1,2-bis-(9-borafluorenyl)-*ortho*-carborane, C_2_B_10_H_10_-1,2-[B(C_12_H_8_)]_2_ ([2a]) featuring *o*-carborane as the inorganic three-dimensional backbone and the synthesis of 1,2-bis-(9-borafluorenyl)benzene, C_6_H_4_-1,2-[B(C_12_H_8_)]_2_ (2b), its phenylene analog. DFT calculations on the transition state for the rearrangement support an intramolecular C–H bond activation process *via* an S_E_Ar-like mechanism in [2a], and predicted that the same rearrangement would take place in 2b, but at elevated temperatures, which indeed proved to be the case. The rearrangement gives access to 3a and 3b as dibora-benzo[*a*]fluoroanthene isomers, a form of diboron polycyclic aromatic hydrocarbon (PAH) that had yet to be explored. The isolated compounds 2b, 3a, and 3b were fully characterized by NMR, HRMS, cyclic voltammetry (CV), single-crystal X-ray diffraction analysis, and photophysical measurements, supported by DFT and TD-DFT calculations.

## Abstract 2

The backbone linker for two *ortho*-bis-(9-borafluorene)s influences the LUMO at the boron centers and the reactivity of the respective compounds.

## Graphical abstract

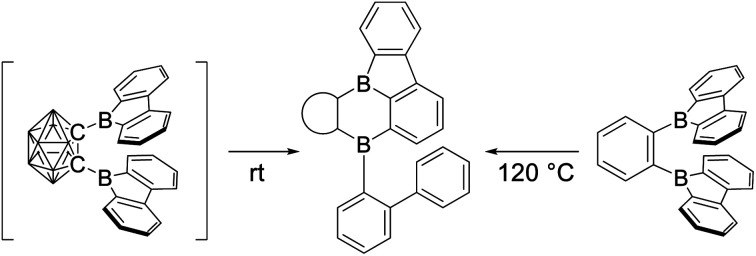

## Introduction

For several decades, 3-coordinate boron, as a carbenium analog, has been of significant interest for use in connected π-systems as a neutral p-doping option. The trigonal planar boron and its perpendicular, vacant p-orbital lead to its use as a strong π-acceptor moiety and as a Lewis acid.^1–6^ Applications include chromophores for biological applications and electrooptical devices,^7–12^ Lewis acids for small molecule activation,^13–18^ and selective small anion sensors.^19–22^

**Fig. 1:**
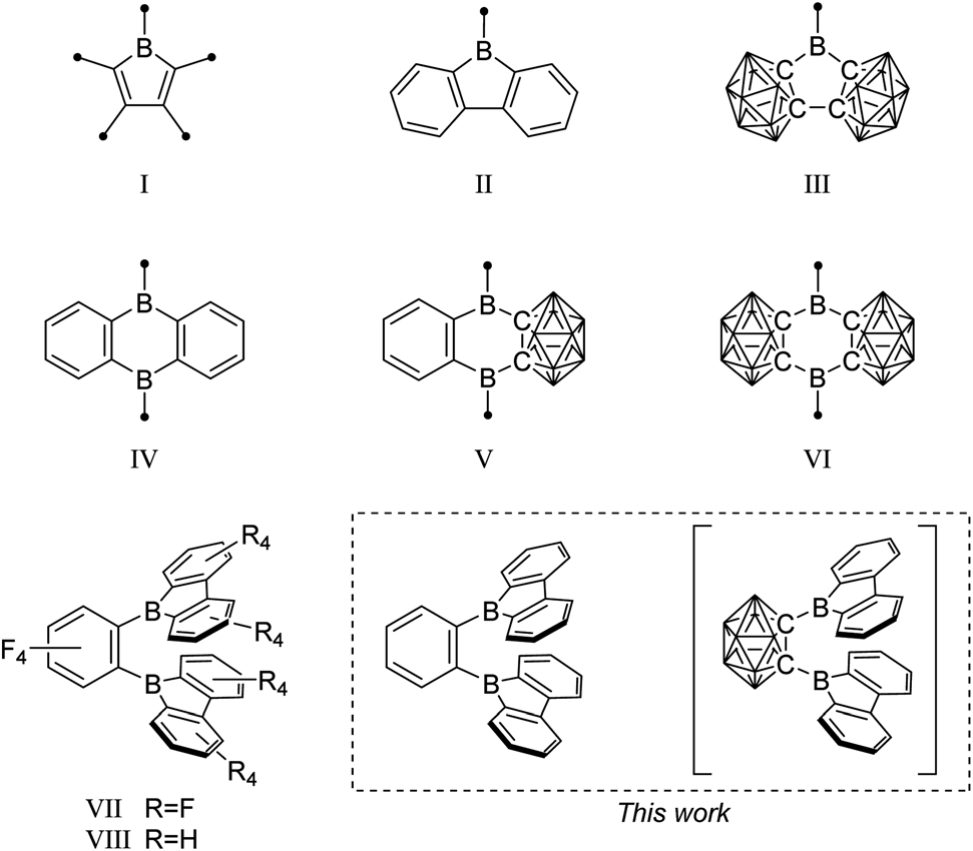
Examples of borole, 9-borafluorene, and 9,10-diboraanthracene motifs, and their 3-dimensional analogs. The unlabeled cluster vertices represent BH units.

Specifically, boroles (I) (Fig. 1), the isoelectronic BC_4_-ring analogs of the 4π-electron cyclopentadienyl cation (Cp^+^), have recently generated considerable interest due to their antiaromatic electron configuration and low HOMO–LUMO energy gap that results in unique reactivities as well as photophysical properties.^23–29^ Benzannulated 9-borafluorenes (II) offer a stabilized version of the otherwise hard to handle boroles, thus facilitating accessibility.^30–32^ Much research has been carried out to investigate the influence of the *exo*-substituent on boron,^33,34^ the fused π-system,^30,35^ its (anti-)aromaticity,^36,37^ and the boron-centered LUMO energy.

Similar to boroles,^24^ 9-borafluorenes are known to undergo ring expansion reactions *via* insertions of heteroatoms,^38–44^ alkynes,^45–47^ and alkenes^48^ into one of the endocyclic B–C bonds. Ring opening reactions with moisture or protic solvents such as alcohols were reported for VII and the generic boroles.^49,50^ The ring-opening polymerization of 9-*H*-9-borafluorenes is well understood as a progression from its preferred *in situ* dimer formation to oligomeric structures utilizing the loss of antiaromaticity and ring strain as the driving force.^51–54^ In the case of the tetraarylborate anion, 9-mes_2_-9-borafluorene (mes = mesityl), and its analogs, ring expansion *via* benzylic C–H activation of one *ortho*-methyl group on the mesityl moiety was observed when irradiated at 300 nm.^55^

In search of increased Lewis acidity, several groups experimented with exchanging boron-bound phenylene backbone systems for C–C-substituted *ortho*-carboranes, locked in plane by both the C1 and C2 vertices. Martin *et al.* published a three dimensional analog of 9-borafluorene (III).^56^ In a subsequent study, Welch *et al.* confirmed the strong Lewis acidic behavior of such compounds.^57^ The same trend was observed for the 9,10-diboraanthracene^58,59^ motif (IV), when phenyl groups were replaced by the carborane moiety (V)^60^ resulting in Lewis superacids that show very low reduction potentials (VI).^61^

*Ortho*-carborane can act as a multifunctional acceptor in charge transfer (CT) processes when connected to π-systems.^62–65^ Additionally, carborane itself has been shown to lower the LUMO energy of boranes, especially when the p_*z*_ orbital on boron aligns with the C1–C2 σ*-anti-bonding orbital.^48,66–68^ A similar effect can be observed when the carborane is attached to boron *via* a conjugated π-system.^68^ It is well-known that C-bound *ortho*-1,2-C_2_B_10_H_11_ exerts a strong inductive electron withdrawing effect.^69,70^

A focus on boron centers and how they interact with a proximal substituent has been prevalent in the literature starting from classical triaryl boranes and their use as bidentate Lewis acid initiators in olefin polymerization chemistry.^71–74^ In this pursuit, a perfluorinated bis-9-borafluorene (VII) further increased the Lewis acidity.^75^ Furthermore, the backbone fluorinated bis-9-borafluorene VIII was reported from a C–F-bond activation of hexafluorobenzene using the dianion of a 9-borafluorene dimer.^76^ Additionally, one-electron bonds were studied in naphthyl-, diphenyl-, or methyl-bridged bis-9-borafluorenes.^77–80^

## Results and discussion

### Synthesis and observation

*O*-Carborane has a strong stabilizing effect on the LUMO of an *exo* C-bound boron moiety.^48,66,67^ With this in mind, we planned the synthesis of [2a]. We expected a geometry that would align the *exo* B p_*z*_-orbitals and the C1–C2 σ*-anti-bonding *o*-carborane orbital, maximizing the effect on photophysical and electrochemical properties of 9-borafluorene.

**Scheme 1:**
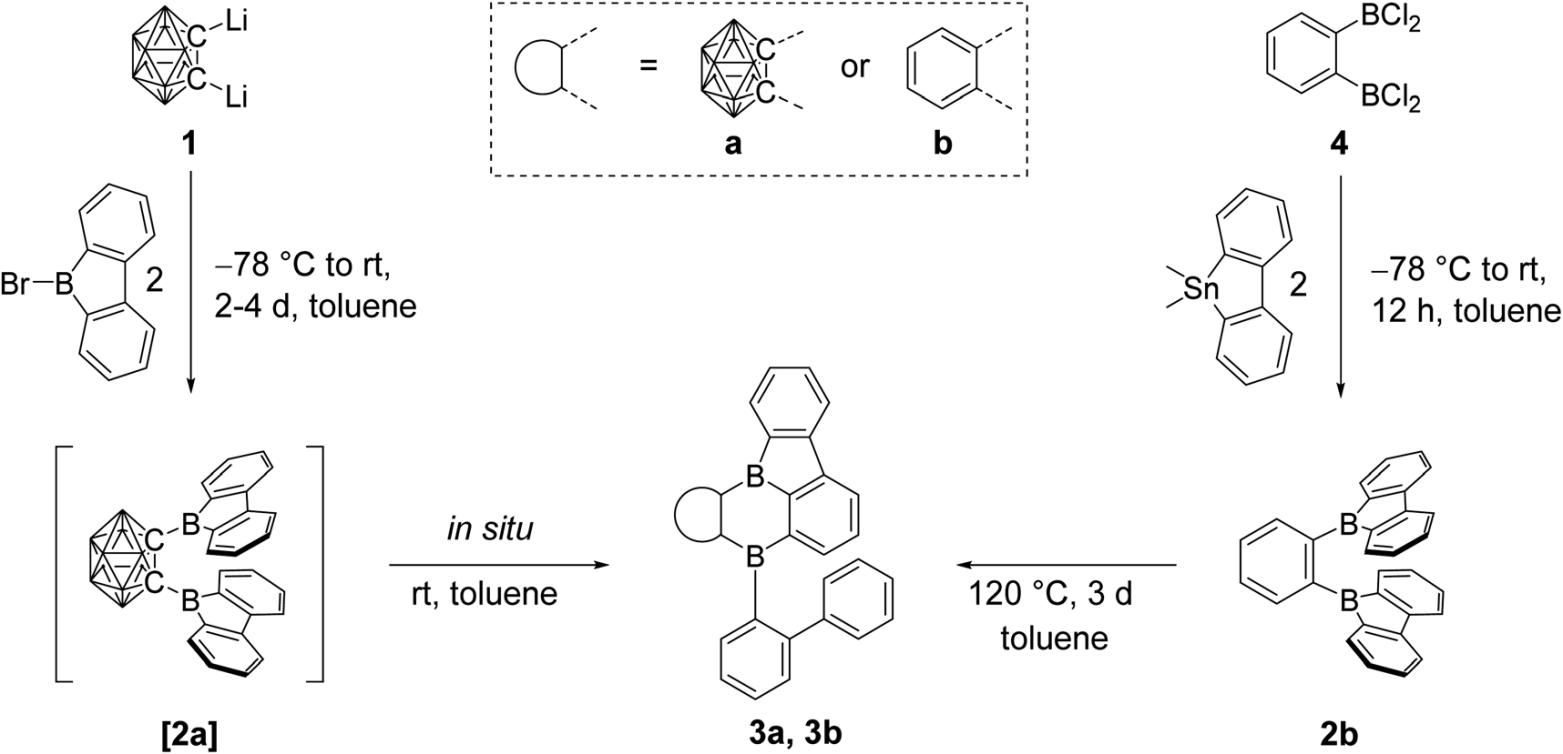
Synthesis of [2a], 2b, 3a, and 3b. The unlabeled cluster vertices represent BH units.

Thus, dilithio *o*-carborane was reacted with two equivalents of 9-bromo-9-borafluorene (Scheme 1). To our surprise, instead of [2a], a bright orange solid 3a was isolated. Compound 3a was characterized by ^1^H, ^11^B and ^13^C{^1^H} NMR spectroscopy, HRMS, and single-crystal X-ray diffraction. The latter revealed that one 9-borafluorene unit is connected to the second *exo* boron center resulting from a C–H bond activation, forming a phenyl-*o*-carborane-B_2_C_4_-subunit similar to that in V. The other 9-borafluorene unit undergoes a ring opening process resulting in an *ortho*-biphenyl substituent (Fig. 3). At this point, we suspected a reaction cascade in which [2a] formed and rearranged yielding 3a as a result of the reaction conditions. The low yield of 13% suggests incomplete conversion or the loss of ionic side products during filtration of the toluene suspension. Compound 3a is prone to nucleophilic attack and was handled strictly under an inert atmosphere.

**Fig. 2:**
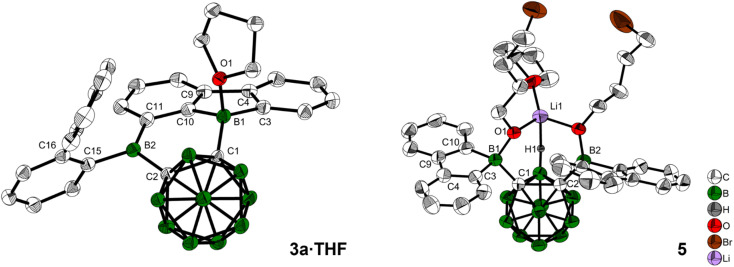
Solid state molecular structures of 3a·THF and 5 from single-crystal X-ray diffraction at 100 K. Atomic displacement ellipsoids are drawn at the 50% probability level and solvent molecules and hydrogen atoms are omitted for clarity. For 5, the second cation [Li(THF)_4_]^+^, the minor parts of disordered THF, and one of the alkyl groups are omitted for clarity. Selected bond lengths (Å) and dihedral angles (deg): for 3a·THF: C1–C2 1.717(2), B1–O1 1.624(3), B1–C1 1.624(3), B2–C2 1.603(3); B1–C1–C2–B2 0.9(2). For 5: C1–C2 1.806(5), B1–C1 1.680(6), B2–C2 1.701(6), H1–Li1 2.15(4); B1–C1–C2–B2 1.1(5).

When 3a was crystallized from toluene *via* hexane diffusion in the presence of a small amount of THF, the mono THF adduct 3a·THF was isolated as single crystals suitable for X-ray diffraction (Fig. 2). This structure suggests that B1 of the five-membered BC_4_-ring is more Lewis acidic than B2. This was confirmed by subsequent DFT-calculations, that show a larger contribution to the LUMO from B1 compared to B2, *vide infra*. The boron carbon bond lengths in 3a·THF are in the expected range for a 4-coordinate, sp^3^-hybridized boron. The loss of conjugation in the π-system results in colorless crystals.

In our initial reactions, we used *n*BuLi (2.1 eq.) for *in situ* lithiation of the *o*-carborane followed by reaction with the 9-bromo-9-borafluorene at elevated temperatures. To reduce the likelihood of competing reactions, due to the high temperatures and the use of excess base, in subsequent studies dilithio *o*-carborane was isolated prior to use as 1,2-Li_2_-1,2-C_2_B_10_H_10_·(Et_2_O)_2_.^81^ From experience, we know that substitution at the second *o*-carborane carbon atom at room temperature requires extended reaction times. The observation of the rearrangement, even at room temperature, led us to investigate the transition state using quantum chemical calculations (*vide infra*). Simultaneously, to confirm the initial formation of [2a], the reaction was monitored at lower temperatures *via* NMR spectroscopy. However, due to the low solubility of the dilithium salt in toluene at or below room temperature, the results were inconclusive. Running the reaction in ether solvents, especially THF, increased the solubility of the dilithium salt significantly, but 9-bromo-9-borafluorene is known to cleave ethers^82,83^ which, in this case, led to the formation of compound 5. Crystals of 5 were isolated and characterized using single-crystal X-ray diffraction (Fig. 2 and ESI†).

**Scheme 2:**
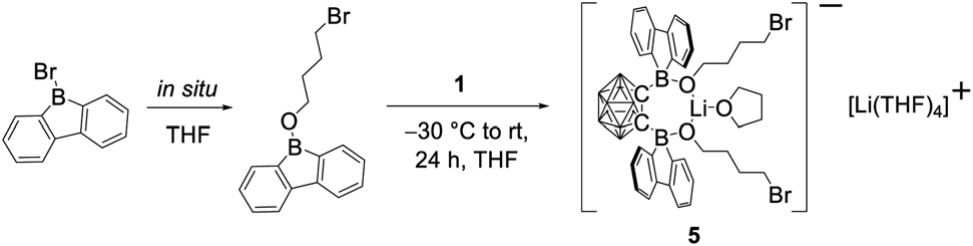
Formation of compound 5. The unlabeled cluster vertices represent BH units.

The structure of 5 shows that two equivalents of 9-bromo-9-borafluorene had already reacted with THF *via* ring opening to form 9-(4-bromobutoxy)-9-borafluorene, which subsequently reacted with the dilithio *o*-carborane generating two 4-coordinate boron centers without elimination of the alkoxy groups (Scheme 2 and ESI†).

The resulting dianionic structure coordinates to one of the corresponding lithium cations *via* the two oxygen atoms and an hydridic cluster B–H (H1–Li1 = 2.15(4) Å). The tetra coordination of the boron centers B1 and B2 in compound 5 inhibits a subsequent rearrangement. The formation of this bis-*ortho*-substituted carborane constitutes important evidence for the *in situ* formation of [2a]. From these experiments we conclude that the rate of the rearrangement reaction is higher than the rate at which [2a] is formed.

**Fig. 3:**
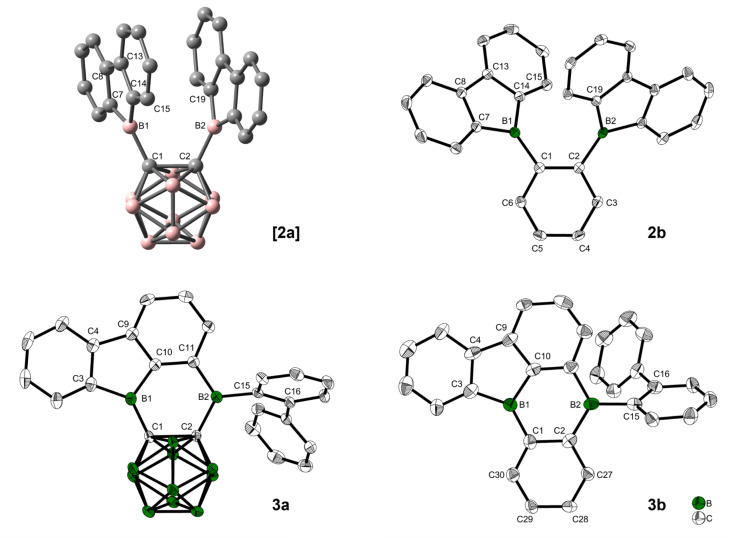
Optimized structure of [2a] at the ωB97X-D/6-31G(d,p) level of theory in the gas phase (B = red, C = grey). Solid–state molecular structures of 2b, 3a, and 3b from single-crystal X-ray diffraction at 100 K. Atomic displacement ellipsoids are drawn at the 50% probability level and hydrogen atoms are omitted for clarity. For 3b, only one of four symmetry-independent molecules is shown, and solvent molecules are omitted for clarity.

Quantum chemical calculations suggested a higher activation barrier for the rearrangement of the analog of [2a] containing a phenylene backbone (2b) as will be discussed in detail later (*vide infra*). As a result, we synthesized 2b, the electron rich analog of its fluorinated predecessors VII and VIII. Transmetallation of a stannafluorene with the known^84^ bis-(dichloroboryl)benzene (4) (Scheme 1) gave a bright yellow solid in an excellent yield of 79%. It is noteworthy that the product formation does not depend on the stoichiometry, as observed in an equimolar reaction. The compound was handled under an inert atmosphere and decomposes if vacuum dried from a CH_2_Cl_2_ solution. The ^11^B NMR spectrum shows a broad resonance at 67.0 ppm, typical of 9-borafluorenes.^31^ Furthermore, ^1^H and ^13^C{^1^H} NMR spectroscopy, along with HRMS and crystal structure analysis, confirm the structure of 2b (Fig. 3).

Unfortunately, a selective route to the carborane analogue of 1,2-(BCl_2_)_2_-1,2-C_2_B_10_H_10_, has not been reported. Reaction of dilithio *o*-carborane with trihaloboranes led to mixtures containing higher order substitutions.^61^ As a result, we did not attempt this route for the synthesis of [2a].

Confirming the quantum chemical prediction, heating compound 2b for three days at 120 °C in toluene led to the formation of a bright orange solid with complete conversion, according to ^1^H NMR spectroscopy. Without the need for further purification, compound 3b was characterized by ^1^H and ^13^C{^1^H} NMR spectroscopy, HRMS, and single-crystal X-ray diffraction. The broad ^11^B NMR signal of 3b is shifted upfield slightly from that of 2b to 63.9 ppm, and only one signal was detected despite the lower symmetry. The rearrangement of 2b is identical, in principle, to the one observed for [2a], in this case resulting in a diboraanthracene-like subunit. UV irradiation for 4 h in a C_6_H_6_ solution at room temperature did not lead to rearrangement.

### Molecular geometries before and after the rearrangement

Solid state molecular structures of 2b, 3a, and 3b are shown in Fig. 3. The optimized structures of 2b, 3a, and 3b at the ωB97X-D/6-31G(d,p) level of theory in the gas phase fit the crystal structures nicely (Table 1). As compound [2a] could not be isolated, the DFT-optimized structure was used for comparisons (Fig. 3).

**Table 1:** Selected bond lengths [Å], distances [Å], and angles [°] of [2a], 2b, 3a, and 3b in the crystal structures and optimized structures at the ωB97X-D/6-31G(d,p) level of theory

	[2a], calc.^a^	2b, crystal	2b, calc.^a^		3a, crystal	3a, calc.	3b, crystal^c^	3b, calc.
C_1_–C_2_	1.674	1.426(2)	1.420	C_1_–C_2_	1.718(2)	1.693	1.43(2)^c^	1.429
C_1_–C_6_/	—	1.402(2)	1.402	B_1_–C_1_–C_2_	114.4(1)	114.3	117.2(5)^c^	117.2
C_2_–C_3_		1.405(2)						
C_5_–C_6_/	—	1.388(2)	1.393	B_2_–C_2_–C_1_	118.5(1)	118.8	122.9(5)^c^	122.7
C_3_–C_4_		1.389(2)						
C_4_–C_5_	—	1.386(2)	1.393	C_1_–B_1_–C_10_	119.5(1)	119.9	117.9(5)^c^	118.5
				C_10_–B_1_–C_3_	105.5(1)	105.1	103.5(5)^c^	103.3
				C_3_–B_1_–C_1_	135.0(1)	134.9	138.6(5)^c^	138.2
B_1_–C_1_^b^	1.600	1.554(2)	1.556	ΣC–B_1_–C	360.0(3)	359.9	360.0(15)^c^	360.0
		1.551(2)						
B_1_⋯B_2_	3.054	3.186(2)	3.083	ΣC–B_2_–C	358.8(3)	359.4	360.0(15)^c^	359.9
B_2_⋯C_15_^b^	3.487	3.252(2)	3.231	B_1_–C_1_–C_2_–B_2_	2.6(2)	3.6	3.0(7)^c^	2.7
		3.354(2)						
C_2_–C_1_–B_1_	115.6	124.9(1)	122.1	C_2_–B_2_–C_15_–C_16_	82.4(1)	79.4	59.3(6)^c^	63.3
C_1_–C_2_–B_2_		123.6(1)						
B_1_–C_1_–C_2_–B_2_	6.3	13.2(2)	9.4					
C_2_–C_1_–B_1_–C_14_^b^	59.5	43.6(1)	43.5					
		39.2(1)						

a The optimized structures of [2a] and 2b have a *C*_2_ axis and therefore equivalent 9-borafluorene moieties.

b Values are given for both symmetry independent 9-borafluorene moieties for the crystal structure of 2b.

c Four symmetry-independent molecules are present in the crystal structure of 3b and the mean values are shown here. The separate values can be found in the ESI.

Crystals suitable for X-ray analysis of 2b were grown from toluene. Although not crystallographically identical, both 9-borafluorenes in the molecule have similar relevant structural parameters within the range of error. The 3-coordinate boron atoms B1 and B2 each form one B–C bond (B1–C1 = 1.554(2) Å and B2–C2 = 1.551(2) Å) to the phenylene-backbone, which may be slightly shorter than those in 9-phenyl-9-borafluorene (1.573(9)/1.58(1) Å), although the large errors in the latter values do not allow a definitive conclusion.^85^ In the solid state, the 9-borafluorenes and the backbone planes are twisted at dihedral angles of 43.6(2)° (C14–B1–C1–C2) and 39.2(2)° (C1–C2–B2–C19), respectively, while the dihedral angle between both 9-borafluorene substituents (B1–C1–C2–B2) is 13.2(2)°. The two 9-borafluorene substituents repel each other slightly, resulting in a B1–C1–C2 angle of 124.9(1)° and a B2–C2–C1 angle of 123.6(1)°. A deformation is visible in the phenylene backbone with the C1–C2 bond (1.426(2) Å) being the longest and C4–C5 (1.386(2) Å), on the opposite side of the phenylene ring, being the shortest bond. The B⋯B distance in 2b (3.186(2) Å) is longer in comparison to the unfused perfluoroaryldiborane C_6_F_4_-1,2-[B(C_6_F_5_)_2_]_2_ (3.138 Å)^72^ and its perfluoro-bis-9-borafluorenyl analog C_6_F_4_-1,2-[B(C_12_F_8_)]_2_ (VII) (3.056 Å).^75^ The distance between atoms B2 and C15 that are involved in the bond formation towards the rearranged compound 3b is 3.252(2) Å.

Crystals suitable for X-ray analysis of 3a were grown from toluene *via* hexane diffusion. The B1 center of this rearranged product, while not perfectly trigonal (C1–B1–C10 = 119.5(1)°, C10–B1–C3 = 105.5(1)°, C3–B1–C1 = 135.0(1)°), is planar with the sum of angles being 360.0(1)°. All of the C–B2–C angles are close to 120°, with the sum being 358.8(1)° in the solid state, only slightly deviating from planarity. In comparison to the carborane-phenyl analog of 9,10-diboraanthracene V (C–C = 1.675(7) Å)^60^ and the carborane–carborane analog of 9,10-diboraanthracene VI (1.667(3) Å),^61^ the C1–C2 carborane bond in 3a is significantly elongated (1.718(2) Å). The extended 9-borafluorene system is nearly planar with internal dihedral angles close to 0° or 180° (B1–C1–C2–B2 = 2.6(2)°, C2–C1–B1–C3 = 176.1(1)°). The *ortho*-biphenyl group is rotated close to a perpendicular orientation (C2–B2–C15–C16 = 82.4(1)°) with respect to the boron-containing π-system.

Crystals suitable for X-ray analysis of 3b were grown from toluene *via* pentane diffusion. The unit cell contains four symmetry-independent molecules. Due to a low bond precision and a large bond length variation between symmetry-independent molecules (see Table S3 in the ESI†), a detailed discussion of experimental B–C and C–C bond length differences is not possible. However, the bond angles and dihedral angles are of sufficient accuracy and vary only slightly between the symmetry-independent molecules, which allows a thorough discussion. Similar to 3a, the angles surrounding the boron center B1 show a strong distortion from perfect trigonal geometry (C10–B1–C3 = 102.4(5)–104.0(5)°, C1–B1–C10 = 116.9(5)–118.8(5)°, C1–B1–C3 = (137.5(5)–140.6(5)°)) with the sum of angles being 360.0(15)°. The second boron center B2 is perfectly trigonal planar with each C–B–C angle being 120° within 3 esd's and the sum of angles being 360.0(15)°. The former backbone shows a similar average bond length for C1–C2 (*ca.* 1.43(2) Å) as that in compound 2b (1.426(2) Å). The biphenyl group bonded to B2 is rotated by 58.2(6)–60.7(6)° with respect to the boron-containing π-system in all four symmetry-independent molecules. The crystal structure of 3b has a π-system with the structure of a di-borabenzo[*a*]fluoroanthene.

A comparison of the optimized starting geometries at the ωB97X-D/6-31G(d,p) level of theory (Table 1) reveals some relevant differences between [2a] and 2b, that can be seen in the C1–C2 bond lengths of 1.674 Å and 1.420 Å, respectively, and in the B1–C1 bond lengths of 1.600 Å and 1.556 Å, respectively. The differences are a direct result of the different backbones and are in their expected range. In general, this should result in a longer distance between the 9-borafluorenyl substituents for the *o*-carborane backbone. However, an attraction in [2a] between the two moieties can be observed in the B1–C1–C2 angle (115.6°), while signs of steric repulsion (122.1°), consistent with the crystal structure, can be observed in 2b, compared to the ideal 120° angle expected for both phenylene and carboranyl backbones. Both compounds show similar B1–B2 distances (3.054 Å ([2a]) and 3.083 Å (2b)) in their optimized geometries which, for 2b, is a significant deviation from the solid-state structure (3.186(2) Å). This could be a result of packing effects in the crystalline state and the calculation being in the gas phase. The dihedral angles between the backbone C–C bond and the 9-borafluorene described by C2–C1–B1–C14 are relevant for possible interactions with the backbone. For 2b, a twist of 43.5° is observed, while [2a] shows a larger angle of 59.5°. The difference in these angles also results in a longer B2⋯C15 distance for [2a] (3.487 Å) compared to 2b (3.231 Å), where the new bond is formed in the rearrangement.

### Quantum chemical transition state calculations

DFT calculations were carried out using different DFT functionals (ωB97X-D, M062X, and B3LYP-D3(BJ)) to provide further insight into the rearrangement processes of [2a] and 2b with a focus on the differences observed for the carboranyl and phenylene backbones. More information on the calculated geometries can be found in the ESI (Tables S2–S4†).

**Fig. 4:**
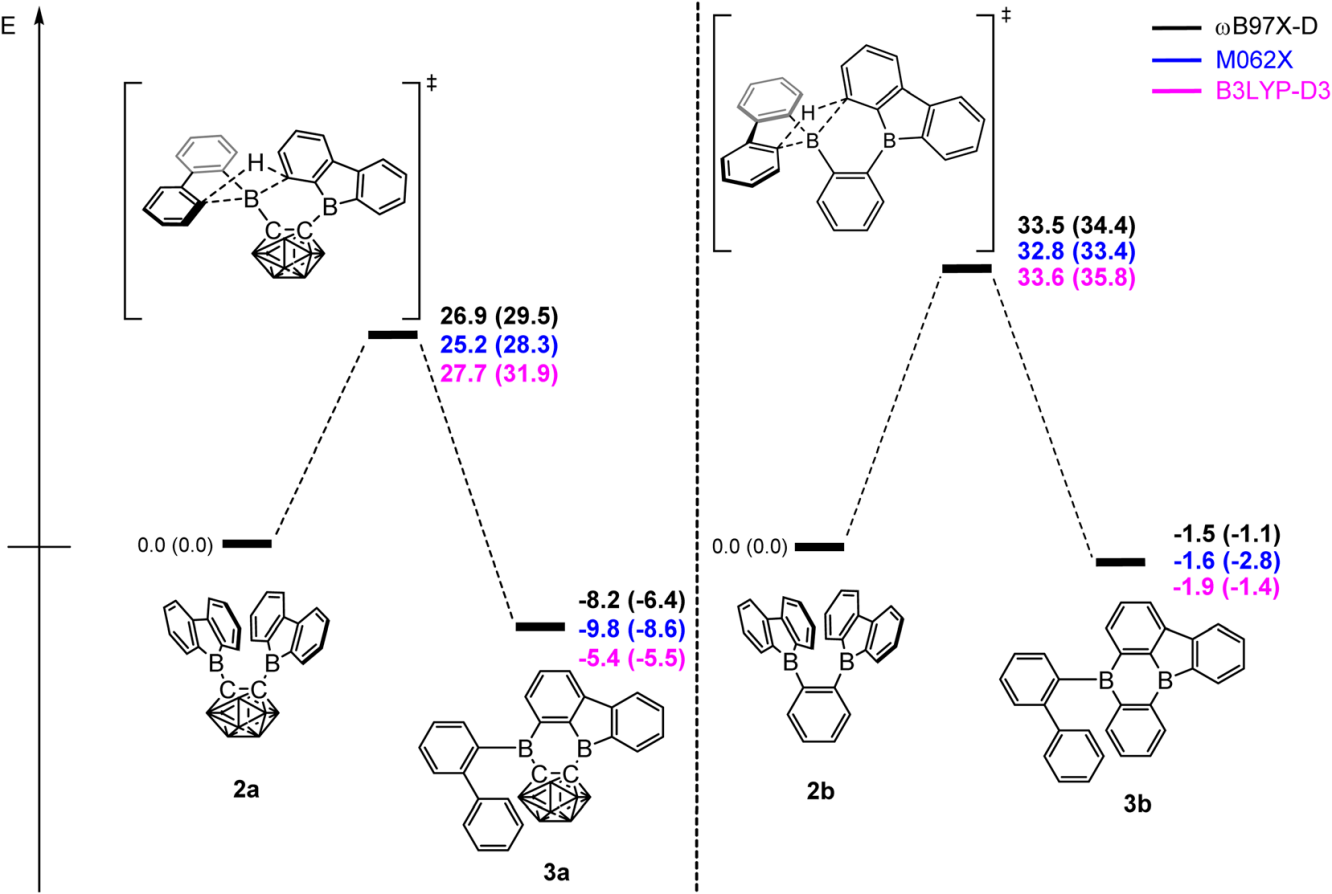
Energy profiles calculated for the rearrangement processes for [2a] (left) and 2b (right). The relative Gibbs free energies and electronic energies (in parenthesis) are given in kcal mol^−1^. The unlabeled cluster vertices represent BH units.

**Fig. 5:**
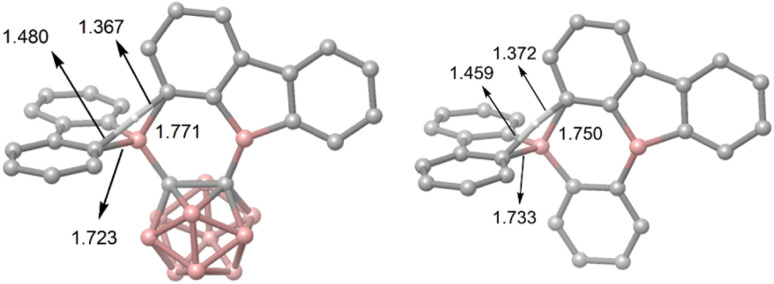
The two calculated transition state structures at the ωB97X-D level of theory for the rearrangement processes shown in Fig. 4 involving C–H bond activation. Bond lengths are shown in Å.

Fig. 4 shows that the resulting energy profiles calculated with these functionals are very similar. In both cases, the rearrangement follows the path of an electrophilic aromatic substitution. The rearrangement starts with the bis-9-borafluorenyl *ortho*-substituted species which offer boron-centered LUMOs and aromatic electron density in proximity. The calculated rearrangement transition state structures involve C–H bond activation *via* an S_E_Ar-like mechanism, resembling a concerted electrophilic aromatic substitution process having a Wheland-like transition state (Fig. 4 and 5). After the proton shift and the associated BC_4_-ring opening, the arene σ-complex evolves to form a new planar system having a biphenyl substituent.

**Fig. 6:**
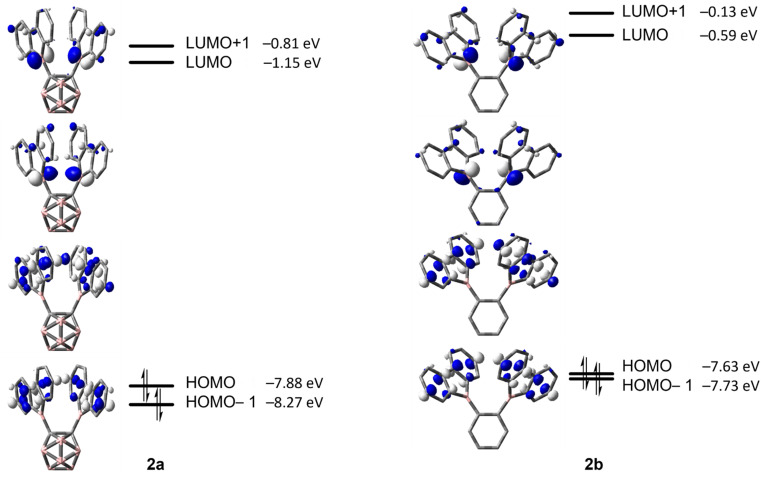
HOMO−1, HOMO, LUMO and LUMO+1 energies calculated at the ωB97X-D/6-31G(d,p) level of theory for [2a] and 2b (isovalue: ±0.06 [e Å^−3^]^1/2^).

The energy barriers calculated for the rearrangement of [2a] are within the range 25.2–27.7 kcal mol^−1^, a result that is reasonable considering a reaction time of 48 h or more. The activation barrier for the rearrangement process of 2b is *ca.* 7 kcal mol^−1^ higher, confirming our experimental observation that 2b is kinetically much more stable than [2a]. Furthermore, the calculations correctly predicted the rearrangement at elevated temperatures. The significantly lower barrier for the rearrangement of [2a] is associated with a relatively higher exothermicity of the rearrangement, consistent with the notion that a C-connected *o*-carboranyl group is highly electron-withdrawing, which facilitates the C–H bond activation process. This effect can be seen in the stabilized LUMO energy of [2a] (Fig. 6). The small energy difference between 2b and 3b suggests a possible equilibrium that was not observed experimentally. In contrast, 3a is thermodynamically significantly more stable than [2a].

### Photophysical properties and TD-DFT calculations

The photophysical properties of compounds 2b, 3a, and 3b were investigated, and observations rationalized *via* complementary DFT and TD-DFT calculations. The photophysical data are summarized in Table 2. The ground state structures were first optimized in the gas phase at the B3LYP/6-31+G(d, p) level of theory starting from their crystal structures. As previous studies have shown the need for range-separated hybrid functionals in CT transitions, TD-DFT calculations were done using the CAM-B3LYP functional with and without a hexane solvent sphere (ESI†).^33,86,87^ We determined the degree of CT character by calculating the orbital overlap parameter *Λ* for the transitions (Table 3 and ESI†).

**Table 2:** Photophysical data of compounds 2b, 3a and 3b

Compound	Solvent	*λ*_abs_ [nm]	*λ*_em_ [nm]^a^	Apparent stokes shift [cm^−1^]	*τ* [ns]	*Φ*
2b	Solid	440^b^	540	4200	126	0.49
3a	Hexane	438, 377,^c^ 323, 299	568	5200	100^d^	0.13^d^
	Toluene	450, 372,^c^ 325, 295	592	5300	50^d^	0.12^d^
	CH_2_Cl_2_	451,^c^ 327, 301	596	5400	39^d^	0.12^d^
	Solid	499^b^	604	3500	44	0.19
3b	Hexane	450, 380, 350, 320	548, 580	4000, 5000	84 (86%)^d^	0.07^d^
					129 (14%)^d^	
	Solid	450,^b^ 500^b^^,^^c^	583	5100, 2800	33 (20)	0.11
					86 (80)	

a Excited at the respective *λ*_abs,max_ of the S_1_ ← S_0_ transition.

b Calculated from excitation spectrum (see ESI).

c Approximate values due to the lack of clear maximum.

d Low reliability due to visible decomposition.

**Table 3:** Lowest energy transitions with a 9-borafluorene character calculated at the CAM-B3LYP/6-31+G(d,p) level of theory for 2b in the gas phase and for 3a and 3b in *n*-hexane

Compound	State	*E* [eV]	*λ* [nm]	*f*	Symmetry	Major contributions	*Λ*
2b	S_1_ ← S_0_	3.07	403	0.0016	A	HOMO → LUMO (66%)	0.61
						H−1 → L+1 (30%)	
	S_2_ ← S_0_	3.10	401	0.0001	A	H−1 → LUMO (62%)	0.61
						HOMO → L+1 (35%)	
	S_3_ ← S_0_	3.97	313	0.1529	A	H−2 → LUMO (83%)	0.61
3a	S_1_ ← S_0_	2.90	427	0.0021	A	H−1 → LUMO (68%)	0.51
						HOMO → LUMO (25%)	
	S_2_ ← S_0_	3.75	331	0.059	A	HOMO → LUMO (52%)	0.41
						H−1 → LUMO (16%)	
						HOMO → L+1 (12%)	
	S_5_ ← S_0_	4.32	287	0.4836	A	H−4 → LUMO (39%)	0.63
						H−1 → L+1 (30%)	
						HOMO → L+1 (10%)	
	S_15_ ← S_0_	5.23	237	0.4388	A	H−7 → LUMO (13%)	0.62
						H−6 → LUMO (11%)	
						H−4 → L+1 (35%)	
3b	S_1_ ← S_0_	2.88	430	0.0031	A	HOMO → LUMO (91%)	0.51
	S_2_ ← S_0_	3.53	351	0.0091	A	H−2 → LUMO (89%)	0.60
	S_3_ ← S_0_	3.69	336	0.0738	A	H−1 → LUMO (62%)	0.34
						H−3 → LUMO (16%)	
	S_7_ ← S_0_	4.37	284	0.4162	A	H−6 → LUMO (32%)	0.62
						H−4 → LUMO (14%)	
						HOMO → L+1 (21%)	
	S_12_ ← S_0_	4.98	249	0.5429	A	H−8 → LUMO (10%)	0.55
						H−2 → L+1 (39%)	
						H−1 → L+1 (12%)	

**Fig. 7:**
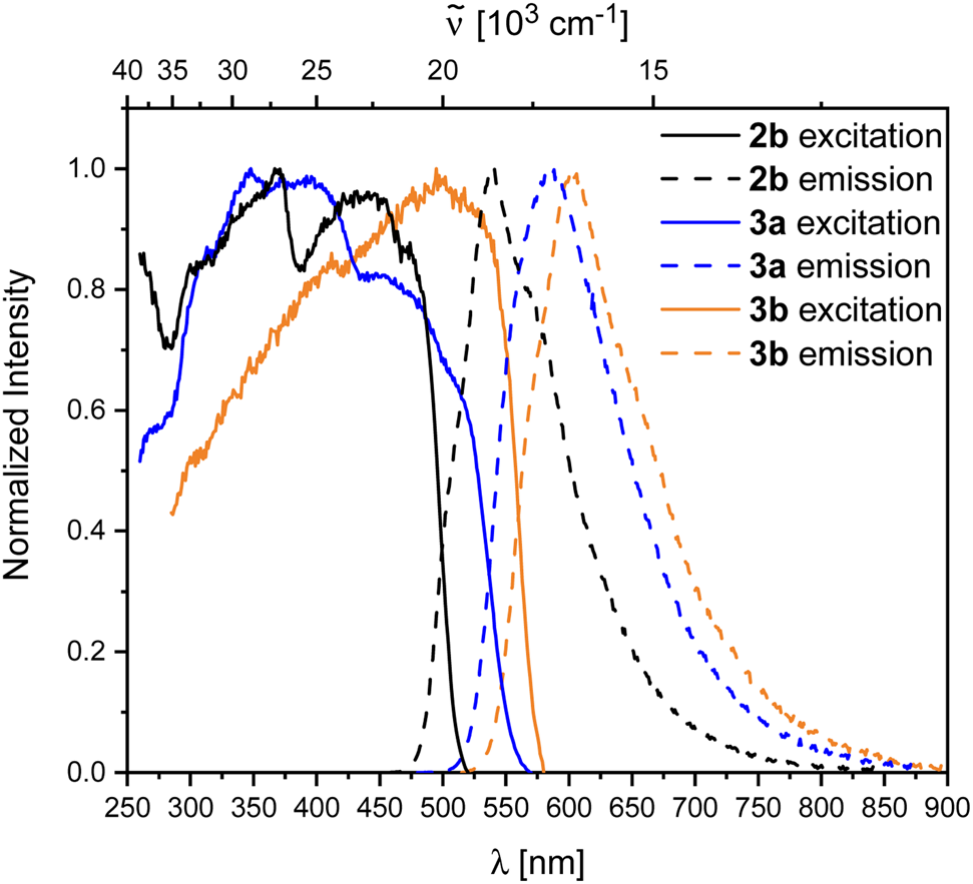
Normalized excitation and emission spectra of the compounds 2b (black), 3a (blue), and 3b (orange) in the solid state.

Compound 2b showed rapid decomposition in solution, so providing reliable data was only possible from solid-state experiments. The excitation and emission spectra of 2b, 3a, and 3b are depicted in Fig. 7. For 2b, we observe a broad absorption reaching 500 nm with its maximum at 440 nm and an emission maximum at 540 nm. Both emission and excitation maxima are in the range of previously reported non-donor-functionalized 9-borafluorene derivatives (*λ*_abs_ ≈ 400 nm, *λ*_em_ ≈ 510 nm).^30^ Similar to other 9-borafluorene compounds,^33–35,88,89^2b exhibits a very long fluorescence lifetime (*τ*_F_ = 126 ns), suggesting a small transition dipole moment. The quantum yield (*Φ*_F_ = 0.49) is comparatively high for borafluorenes, and is the highest quantum yield reported for 9-borafluorenes in the solid state.

In comparison to our previous results,^33^ this suggests a weakly allowed localized excitation (LE) on the borafluorene moieties. This is supported by TD-DFT calculations carried out at the CAM-B3LYP/6-31+G(d, p) level of theory in the gas phase. The lowest energy S_1_ ← S_0_ and S_2_ ← S_0_ transitions exhibit very low oscillator strengths and can be classified as weakly allowed transitions (Table 3). HOMO and HOMO−1, as well as LUMO and LUMO+1 are situated on the borafluorene moieties with only minor contributions from the phenylene moiety (Fig. 9).

Photophysical properties of 3a and 3b in the solid state show a significant resemblance to those of 2b, with a broad excitation to *ca.* 500 nm and emissions at 604 and 583 nm for 3a and 3b, respectively. Unlike 2b, the rearranged compounds exhibit significantly lower quantum yields of *Φ*_F_ = 0.19 and 0.11 for 3a and 3b, respectively.

**Fig. 8:**
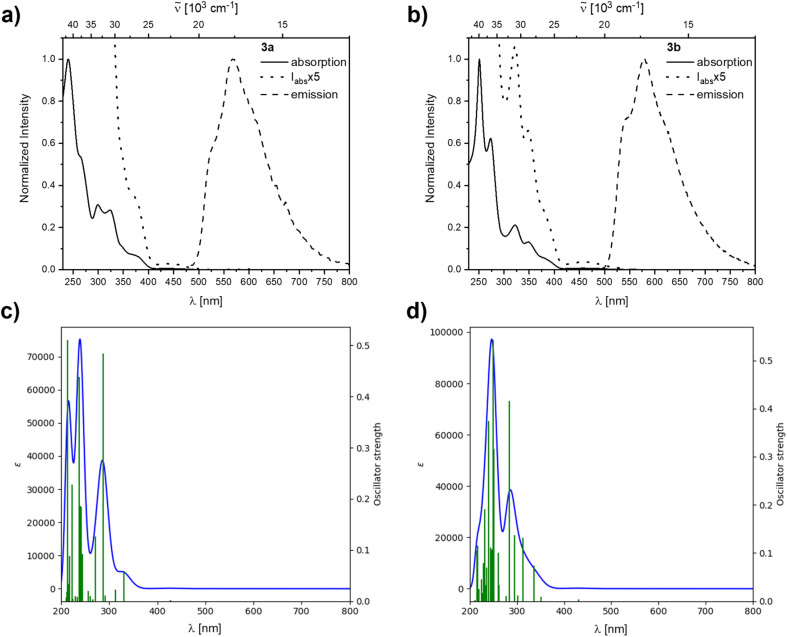
Normalized absorption and emission spectra of the rearranged compounds 3a (a) and 3b (b) in hexane. The dotted lines are blowups shown to visualize the lowest energy transitions. Simulated spectra calculated at the CAM-B3LYP/6-31+G(d,p) level of theory in a hexane solvent model for 3a (c) and 3b (d).

Similar to, but slower than for compound 2b, rapid decomposition of compounds 3a and 3b was observed, and extinction coefficients could not be determined reliably. The photophysical properties of the rearranged compounds 3a and 3b in solution retain their 9-borafluorene-like transitions (Fig. 8). Both compounds show very weak transitions in hexane at 438 and 450 nm, respectively. TD-DFT calculations at the CAM-B3LYP/6-31+G(d,p) level of theory confirm weakly allowed transitions in this energy region with small transition dipole moments and, generally, give a good fit to the experimental spectra (Fig. 8).

**Fig. 9:**
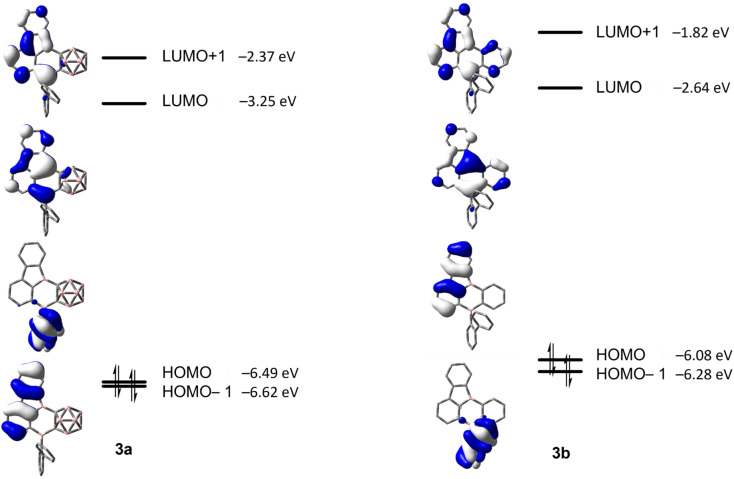
HOMO−1, HOMO, LUMO and LUMO+1 energies calculated at the CAM-B3LYP/6-31+G(d,p) level of theory for 3a and 3b (isovalue: ±0.03 [e Å^−3^]^1/2^).

For 3a and 3b, the lowest energy transition has a strong resemblance to that in typical 9-borafluorenes in the orbital distribution across the π-system (Fig. 9 and Table 3). With the resemblance to benzo[*a*]fluoranthene, the additional boron center and the resulting loss in symmetry, the rearranged compounds 3a and 3b show slightly larger transition dipole moments and an increase in CT character (*Λ* ≤ 0.6). Interestingly, the calculated 9-borafluorene-like HOMO appears as HOMO−1 in the carborane-containing compound 3a. Thus in 3a, there is a significant stabilization of the 9-borafluorene-like HOMO−1 due to the strong –I inductive effect of the *o*-carborane.^69,70^ This inductive effect is known to stabilize connected 3-coordinate boranes and adjacent π-systems.^66,90–93^ This stabilization can also be observed for the LUMO and LUMO+1, but is weaker for the biphenyl HOMO due to its unfavorable out of plane rotation. For 3b, the higher energy of the comparable orbital (HOMO) can also be explained by the increase in PAH-HOMO energies with increasing number of aromatic carbon atoms in the system.^94–97^

**Fig. 10:**
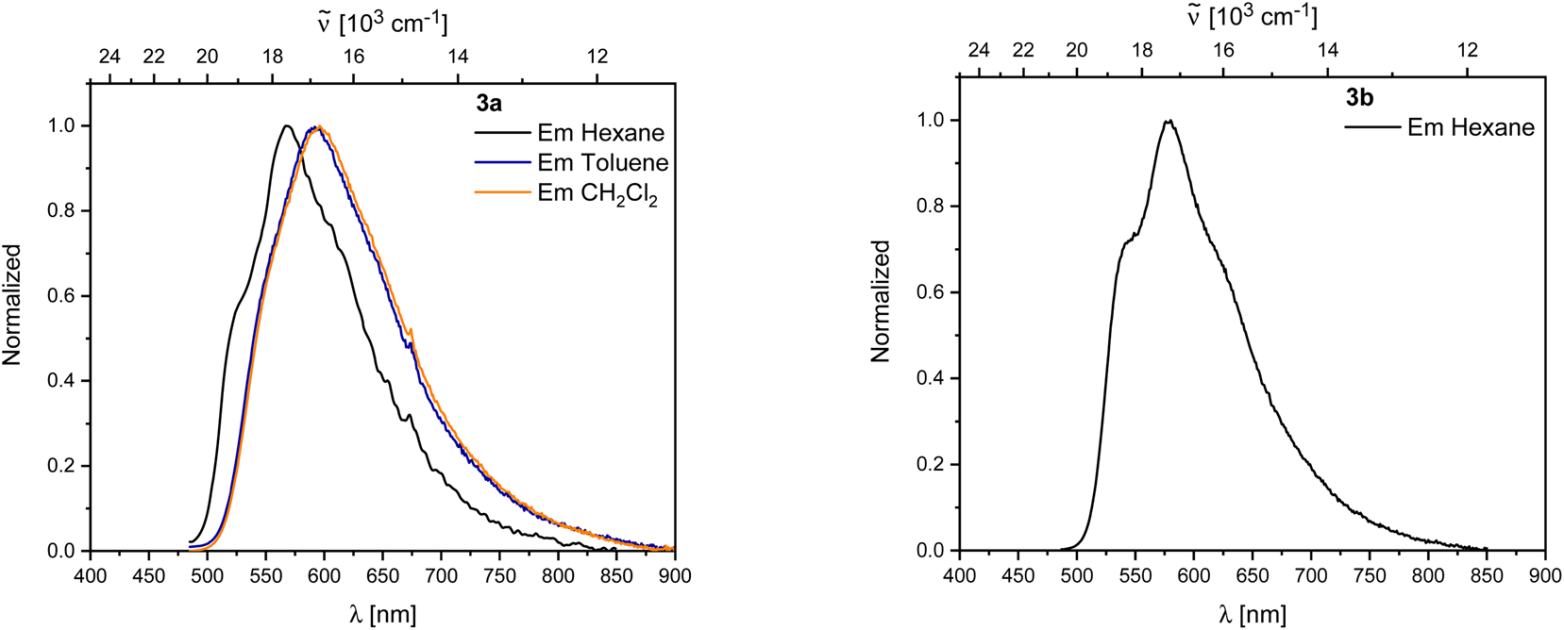
Left: Emission spectra of 3a in hexane (black), toluene (blue), and CH_2_Cl_2_ (orange). Right: Emission spectra of 3b in hexane.

Both 3a and 3b exhibit yellow to orange, low energy emissions at 568 and 580 nm in hexane, respectively, showing a broad vibrational progression that is lost in solvents of higher polarity for 3a. For 3a, a bathochromic shift in the emission, depending on the solvent polarity, is observed from 568 nm in hexane to 596 nm in dichloromethane (Fig. 10).

Decomposition was observed in solution for both rearranged compounds and should be kept in mind regarding the reliability of quantitative measurements. Both compounds show long lifetimes of *ca.* 100 ns in hexane with low quantum yields (*Φ*_F_ = 0.13 (3a) and 0.07 (3b)), indicating weakly allowed transitions (Table 2).

Furthermore, we were interested in the BC_4_-ring antiaromaticity of both compounds. Calculations of out of plane nucleus-independent chemical shift values (NICS(1)_*zz*_) at the B3LYP/6-31+G(d,p) level of theory show higher values for 3a, being 26.4 and 24.5 for 3a and 3b, respectively. The NICS(1)_*zz*_ values are in the range of other reported benzannulated boroles.^33^

### Electrochemical properties

**Fig. 11:**
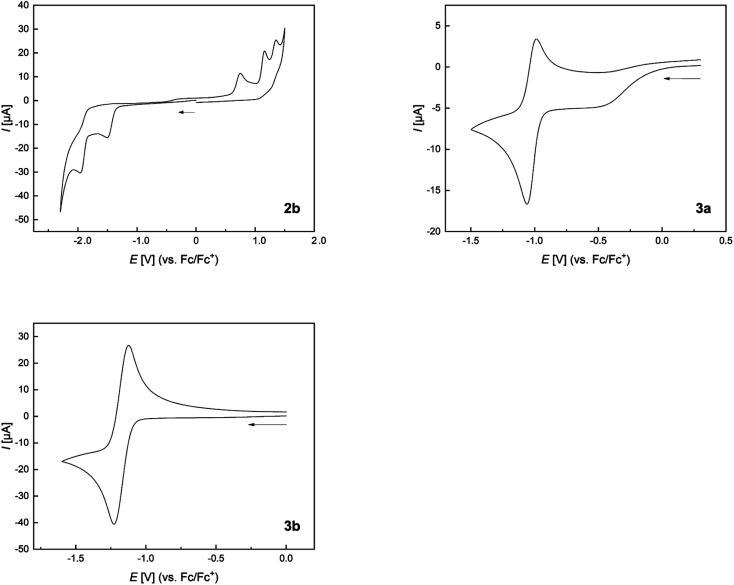
Normalized cyclic voltammograms of 2b, 3a, and 3b as solutions in CH_2_Cl_2_ at room temperature; supporting electrolyte, [*n*Bu_4_N][PF_6_] (0.1 mol L^−1^); scan rate, 200 mV s^−1^; *vs.* Fc/Fc^+^. Additional information can be found in the ESI.†

The cyclic voltammogram of 2b shows an irreversible reduction at *E*_pc_ = −1.5 V and a second irreversible reduction at *E*_pc_ = −1.96 V (Fig. 11 and Table 4). The presence of two borafluorenyl groups is clearly reflected in the first reduction potential, which is significantly lower than that in other 9-borafluorenes.^34^ However, the reduction potentials are in good agreement with related 1,8-naphthalenediyl- and methylene-bridged bisboranes from the groups of Gabbaï and Wagner.^77,78,80^ In addition, the observation of two clearly spaced reduction processes suggests a communication between the two boron centers and the possible formation of a one-electron B–B bond.^77–80^ Compounds 3a and 3b exhibit even lower, at least partially reversible, first reduction potentials at *E*_1/2_ = −1.03 and *E*_1/2_ = −1.17 V, respectively, in agreement with their calculated lower LUMO energies (see Fig. 9). Moreover, the effect of the carborane unit in stabilizing the LUMO is clearly visible.

**Table 4:** Reduction and oxidation potentials of 2b, 3a, and 3b

	1^st^ red. [V]	2^nd^ red. [V]	1^st^ ox. [V]
2b	*E*_pc_ = −1.50	*E*_pc_ = −1.96	*E*_pa_ = 0.98
3a	*E*_1/2_ a = −1.03	*E*_pc_ = −2.14	*E*_pa_ = 0.84
3b	*E*_1/2_ = −1.17	*E*_pc_ = −1.85	—

a Partially reversible reduction.

The extended π-system, which resembles that of benzo[*a*]fluoranthene, involving the two boron atoms in 3a and 3b, explains their lower reduction potentials compared to those in 9,10-diboraanthracenes^58^ and related all-carbon PAHs.^98^ A more apt comparison, especially for 3b, is with the doubly boron-doped PAHs reported by Würthner *et al.*, which also show exceptionally low reduction potentials of up to *E*_1/2_ = −1.07 V.^99^ Comparison with the doubly carborane-fused and π-electron poor variants of 9,10-diboraanthracene (VI) reported by Ye and co-workers, with reduction potentials as low as *E*_pc_ = −0.75 V, further underscores the LUMO-lowering effect of carboranes even without the participation of their C1–C2 σ*-orbitals.^61^

## Conclusions

While attempting the synthesis of 1,2-bis-9-borafluorenyl-*o*-carborane ([2a]), using *ortho*-carborane as a three-dimensional backbone to lower the boron based LUMO energies in 9-borafluorenes, we observed an intramolecular rearrangement at room temperature. Investigation of the transition state using DFT calculations showed that the rearrangement follows an S_E_Ar-like mechanism that is enabled by a low lying LUMO, the targeted feature of compound [2a]. The same calculations for a phenyl-backbone predict a significantly higher energy barrier for such a rearrangement. As a result, we were able to synthesize and characterize 1,2-bis-9-borafluorenylbenzene (2b), the phenylene analog of [2a] and VII, following a tin–boron exchange route. The crystal structure analysis of 2b shows the two 9-borafluorene units in a face-to-face arrangement with a dihedral angle of 43.5° with respect to the phenylene backbone. In solution, communication between the two boron centers was observed in the CV measurement. Reliable photophysical data were obtained from the solid state, showing typical 9-borafluorene properties and a high quantum yield with respect to comparable compounds. After confirming the predicted rearrangement in compound 2b, products 3a and 3b were isolated and fully characterized. The single-crystal structures show very similar structural motifs that differ by the phenylene-/*o*-carboranyl ring and, therefore, by the size of the π-system and inductive effects of the cluster. Isomers of this diborane motif have been suggested for use in organic light-emitting devices.^100^ Our photophysical studies show weak absorption bands at *ca.* 450 nm and yellow emissions at *ca.* 590 nm. TD-DFT calculations and experimental observations agree with a 9-borafluorene-like transition character. A strong electron affinity was observed for 3a and 3b with reduction potentials of *E*_1/2_ = −1.03 and *E*_1/2_ = −1.17 V, respectively. In general, the reactivity shown herein offers a pathway to BC_4_B-six membered ring-containing PAHs for phenylene and, especially, carborane-containing systems. In principle, following the bis-*ortho* substitution motif, other tri-aryl substituents that offer a β-CH unit might rearrange in a similar fashion.

## Data availability

Additional data and photophysical spectra, and crystallographic data, NMR spectra, and Cartesian coordinates for calculations can be found in the ESI.†

## Author contributions

J. K., A. H., S. F., and L. J. carried out the synthesis and single-crystal growth; J. K., S. F., A. F., and A. E. carried out the X-ray crystallographic studies; J. K. and F. R. carried out the photophysical studies; X. G. and Z. L. carried out the transition state calculations; J. K. carried out the TD-DFT calculations; I. K. carried out the cyclic voltammetry studies; L. J., Z. L., H. B., and T. B. M. supervised the overall project; all authors were involved in the preparation of the manuscript.

## Conflicts of interest

There are no conflicts to declare.

## Supplementary Material

SC-013-D2SC06057D-s001

SC-013-D2SC06057D-s002
