# Supplementary material for: Backbone-controlled LUMO energy induces intramolecular C–H activation in ortho-bis-9-borafluorene-substituted phenyl and o-carboranyl compounds leading to novel 9,10-diboraanthracene derivatives
Source: Chem Sci. 2022 Nov 23;13(47):14165–78. doi: 10.1039/d2sc06057d (PMC9728567; doi:10.1039/d2sc06057d)
Supplement: SC-013-D2SC06057D-s001 [file SC-013-D2SC06057D-s001.pdf]

Electronic Supplementary Material (ESI) for Chemical Science.
This journal is © The Royal Society of Chemistry 2022

# Backbone-controlled LUMO energy induces intramolecular C–H activation in *ortho*-bis-9-borafluorene-substituted phenyl and *o*-carboranyl compounds leading to novel 9,10-diboraanthracene derivatives

Johannes Krebs,[a]† Alena Häfner,[a]† Sonja Fuchs,[a] Xueying Guo,[b] Dr. Florian Rauch,[a] Dr. Antonius Eichhorn,[a] Dr. Ivo Krummenacher,[a] Dr. Alexandra Friedrich,[a] Prof. Dr. Lei Ji,[a,c]* Prof. Dr. Maik Finze,[a] Prof. Dr. Zhenyang Lin,[b]* Prof. Dr. Holger Braunschweig,[a]* Prof. Dr. Todd B. Marder[a]*

[a]Institute for Inorganic Chemistry and Institute for Sustainable Chemistry & Catalysis with Boron, Julius-Maximilians-Universität Würzburg, Am Hubland, 97074 Würzburg, Germany (E-mail: h.braunschweig@uni-wuerzburg.de; todd.marder@uni-wuerzburg.de)

[b]Department of Chemistry, The Hong Kong University of Science and Technology, Clear Water Bay, Hong Kong (E-mail: chzlin@ust.hk)

[c]Frontiers Science Center for Flexible Electronics, Xi'an Institute of Flexible Electronics (IFE), Northwestern Polytechnical University, 127 West Youyi Road, Xi'an, Shaanxi, P.R. China (E-mail: iamlji@nwpu.edu.cn)

# Supporting Information

## Table of Contents

General experimental details ........................................ S3
Synthetic procedures ........................................ S7
Single-crystal X-ray diffraction ........................................ S27
Geometry tables ........................................ S32
Photophysical data ........................................ S36
Cyclic voltammetry ........................................ S39
DFT and TD-DFT results ........................................ S41
References ........................................ S69

# General experimental details

Unless otherwise noted, the following conditions apply.

All syntheses were carried out using standard Schlenk and glovebox techniques under an argon atmosphere. The solvents used were dried using either a solvent purification system (SPS) from Innovative Technology or were distilled and degassed from appropriate drying agents and stored under argon. Deuterated solvents ($CD_2Cl_2$ and $C_6D_6$) used for NMR spectroscopy were purchased from Cambridge Isotope Laboratories. $C_6D_6$ and $CD_2Cl_2$ were dried over molecular sieves, degassed by three freeze-pump-thaw cycles and stored under an argon atmosphere prior to use. *n*-Butyllithium (2.5 M solution in hexane) was purchased from Acros Organics and used as received. The compounds 9-bromo-9-borafluorene[1] and 1,2-bis(dichloroboryl)benzene[2] were prepared according to literature procedures. The dilithiated carborane 1,2-$Li_2$-1,2-$C_2B_{10}H_{10}$ was prepared *in situ* according to a published procedure.[3] Isolation of 1,2-$Li_2$-1,2-$C_2B_{10}H_{10}$ from lithiation in toluene at 80 °C overnight does not result in a fully dilithiated product, as a significant portion of monolithiated product remains. To lower the amount of side product formation and problems of identification in NMR experiments, we synthesized 1,2-$Li_2$-1,2-$C_2B_{10}H_{10}$ in $Et_2O$ following a known route.[4] This results in the formation of 1,2-$Li_2$-1,2-$C_2B_{10}H_{10}\cdot(Et_2O)_2$. All other starting materials were purchased from commercial sources and were used without further purification.

**NMR** Spectra were recorded on a Bruker Avance 500 FT NMR spectrometer (operating at $^1$H: 500 MHz, $^{11}$B: 160 MHz, $^{13}$C{$^1$H}: 126 MHz) or Bruker Avance III HD 300 spectrometer (operating at $^1$H: 300 MHz, $^{11}$B: 96 MHz, $^{13}$C{$^1$H}: 75 MHz). Chemical shifts ($\delta$) are given in ppm and $^{11}$B{$^1$H} NMR spectra are referenced to external $BF_3\cdot Et_2O$. $^1$H NMR spectra were referenced via residual proton resonances of $CD_2Cl_2$ (5.32 ppm), $C_6D_6$ (7.16 ppm), and THF-$d_8$ (1.72 ppm). $^{13}$C{$^1$H} spectra were referenced to $CD_2Cl_2$ (53.84 ppm), $C_6D_6$ (128.06 ppm), and THF-$d_8$ (25.31 ppm).

**HRMS** were recorded using a Thermo Scientific Exactive Plus Orbitrap MS system by Liquid Injection Field Desorption Ionization (LIFDI) or an Atmospheric Sample Analysis Probe (ASAP).

**Single-crystal X-ray diffraction:** Crystals suitable for single-crystal X-ray diffraction were selected, coated in perfluoropolyether oil or polybutyl oil, mounted on a polyimide microloop

(MicroMounts fromMiTeGen) and transferred to a stream of cold nitrogen (Oxford Cryostream 700 or 800, respectively). Diffraction data were collected on a Bruker X8 Apex II 4-circle diffractometer with a CCD area detector, using Mo-$K_\alpha$ radiation generated by a Nonius FR591 rotating anode and monochromated by graphite (**3a**) or by multi-layer focusing mirrors (**2b**). Diffraction data were collected on a Rigaku Oxford Diffraction XtaLAB Synergy diffractometer with a semiconductor HPA-detector (HyPix-6000 or HyPix-Arc-150) and multi-layer mirror monochromated Cu-$K_\alpha$ radiation generated by a PhotonJet (**3a·THF**) or a PhotonJet-R (**3b, 5, 9-(4-bromobutoxy)-9-borafluorene**, **9-($Me_2S$)-9-Br-9-borafluorene**, and **1-MeS-2-($Me_2S$-9-borafluorene)-1,2-$C_2B_{10}H_{10}$**) source. Data were collected at 100 K or 173 K (**5**). The images were processed and corrected for Lorentz-polarization effects and absorption (empirical scaling) as implemented in the Bruker software packages (**2b** and **3a**) or using the CrysAlis$^{Pro}$ software from Rigaku Oxford Diffraction (**3b**, **3a·THF**, **5**, **9-(4-bromobutoxy)-9-borafluorene**, **9-($Me_2S$)-9-Br-9-borafluorene**, and **1-MeS-2-($Me_2S$-9-borafluorene)-1,2-$C_2B_{10}H_{10}$**). The structures were solved using the intrinsic phasing method (SHELXT)[5] and Fourier expansion technique. All non-hydrogen atoms were refined in anisotropic approximation, with all hydrogen atoms 'riding' in idealized positions, by full-matrix least squares against $F^2$ of all data, using SHELXL[6] software and the SHELXLE[7] graphical user interface. In the case of **3a** disordered solvent was masked using SQUEEZE/PLATON.[8] **3b** and **3a·THF** were refined as two-component twins, both with twin fractions of 48%. **1-MeS-2-($Me_2S$-9-borafluorene)-1,2-$C_2B_{10}H_{10}$** was refined as two-component twin with a twin fraction of 43%. Diamond software was used for graphical representation.[9] Crystal data and experimental details are listed in Table S1. Full structural information has been deposited with the Cambridge Crystallographic Data Centre. CCDC-2174245 (**2b**), 2174247 (**3a**), 2174246 (**3b**), 2174248 (**3a·THF**), 2174249 (**5**), 2216649 (**9-(4-bromobutoxy)-9-borafluorene**), 2216647 (**9-($Me_2S$)-9-Br-9-borafluorene**), and 2216648 (**1-MeS-2-($Me_2S$-9-borafluorene)-1,2-$C_2B_{10}H_{10}$**).

**Photophysical measurements:** All measurements were performed in standard quartz cuvettes (1 cm x 1 cm cross-section). UV–visible absorption spectra were recorded using a Perkin Elmer Lambda 465 UV-visible spectrophotometer. **Emission spectra** were recorded using an Edinburgh Instruments FLSP920 spectrophotometer equipped with a double monochromator for both excitation and emission, operating in right-angle geometry mode, and all spectra were fully corrected for the spectral response of the instrument. **Fluorescence quantum yields** were measured using a calibrated integrating sphere (inner diameter: 150 mm) from Edinburgh Instruments combined with the FLSP920 spectrophotometer described above. For solution-state and solid-state measurements, the longest-wavelength absorption maximum of the

compound in the respective solvent was chosen as the excitation wavelength. **Fluorescence lifetimes** were recorded using the time-correlated single-photon counting (TCSPC) method using the same FLSP920 spectrometer described above. Solutions were excited with a picosecond pulsed diode laser at 376.6 nm. The full width at half maximum (FWHM) of the laser pulses were ca. 70–200 ps, while the instrument response function (IRF) had a FWHM of ca. 1.0 ns, measured from the scatter of a Ludox solution at the excitation wavelength. Decays were recorded to at least 10000 counts in the peak channel with a record length of at least 1000 channels. The band pass of the monochromator was adjusted to give a signal count rate of <10 kHz. Iterative reconvolution of the IRF with one decay function and non-linear least-squares analysis were used to analyze the data. The quality of the fit was judged by the calculated value of the reduced $\chi^2$ and visual inspection of the weighted residuals.

**Computational methods**

All molecular geometries were fully optimized via DFT calculations at the B3LYP-D(BJ), wB97X-D[10] and M062X[11-13]/6-31G(d,p)[14, 15] level of theory. Frequency calculations at the same level of theory were performed to confirm that all stationary points are local minima (no imaginary frequencies) or transition states (one imaginary frequency) and to provide free energies at 298.15 K. Transition states were located using the Berny algorithm and further confirmed by calculations of intrinsic reaction coordinates (IRC)[16] showing that the transition states indeed connect the two relevant minima. All DFT calculations were performed with the Gaussian 09 (D.01) program.[17]

All calculations regarding the photophysical experiments of **2a**, **2b**, **3a**, and **3b** (DFT and TD-DFT) were carried out with the Gaussian 09 (9.E.01)[18] program package and were performed on a parallel cluster system. GaussView (6.0.16) and multiwfn[19] were used to visualize the results, to measure calculated structural parameters, and to plot orbital surfaces (isovalue: ± 0.030 [e $a_0^{-3}$]$^{1/2}$). The ground-state geometries were optimized using the B3LYP functional[20] in combination with the 6-31+G(d,p) basis set.[21, 22]

The orbital overlap parameter was calculated with $\Lambda = \frac{\sum_{i,a} c_{i,a}^2 \langle |\ \varphi_a\ || |\ \varphi_i\ | \rangle}{\sum_{i,a} c_{i,a}^2}$, resulting in $0 \leq \Lambda \leq 1$, where $\Lambda = 0$ corresponds to no overlap and $\Lambda = 1$ corresponds to complete overlap.[23] The ultrafine integration grid and symmetry constraints were used for all molecules. Frequency calculations were performed on the optimized structures to confirm them to be local minima showing no negative (imaginary) frequencies. Based on these optimized structures, the lowest-energy vertical transitions (using the polarizable continuum model) were calculated (singlets, 25 states) by TD-DFT, using the Coulomb attenuated functional CAM-B3LYP[24] as well as

B3LYP. The CAM-B3LYP functional has been shown to more accurately describe CT systems in comparison to B3LYP.[23] The optimized ground-state geometries were used as starting coordinates for TD-DFT geometry optimizations.

# Synthetic procedures

### Bis(bis-9-borafluorenyl)benzene (2b)

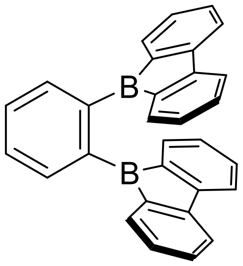

Via syringe, a solution of 1,2-bis(dichloroboryl)benzene **4** (119 mg, 495 $\mu$mol, 1.0 eq.) in 10 mL toluene was slowly added to a solution of dimethyldibenzostannole (300 mg, 990 $\mu$mol, 2.0 eq.) in 10 mL toluene at –78 °C in a Schlenk tube. The solution was stirred and allowed to warm to room temperature overnight. After removal of all volatiles including $Me_2SnCl_2$ *in vacuo*, the residue was washed with hexane (3 x 20 mL) to yield a yellow solid **2b** (158 mg, 393 $\mu$mol, 79%).

**$^{1}$H NMR** (500.1 MHz, $CD_2Cl_2$): $\delta$ = 8.03 (m, 2H, C$H_{Ar}$), 7.70 (m, 2H, C$H_{Ar}$), 7.47 (m, 4H, C*H*), 7.29 (m, 4H, C*H*), 7.23 (m, 4H, C*H*), 6.95 (m, 4H, C*H*) ppm.
**$^{11}$B NMR** (160.5 MHz, $CD_2Cl_2$): $\delta$ = 67.0 (br) ppm.
**$^{13}$C{$^{1}$H} NMR** (125.8 MHz, $CD_2Cl_2$): $\delta$ = 154.03 ($C_{q,Ar}$), 145.03 ($C_{q,Ar}$), 143.78 ($C_{q,Ar}$), 134.81 ($C$H$_{Ar}$), 134.43 ($C$H$_{Ar}$), 134.16 ($C$H$_{Ar}$), 130.70 ($C$H$_{Ar}$), 128.42 ($C$H$_{Ar}$), 120.06 ($C$H$_{Ar}$) ppm.
**HRMS LIFDI** calc. for $[C_{30}H_{20}B_2]^+$ = $[M]^+$: 402.1746, found 402.1744.

**3-([1,1'-biphenyl]-2-yl)-3*H*-1,2-(1,2-*ortho*-carboranyl)-3,10b-diborafluoranthene (3a)**

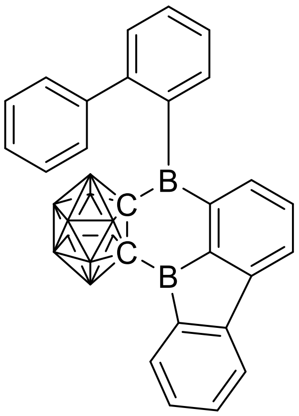

*Ortho*-carborane (200 mg, 1.39 mmol, 1.0 eq.) was dissolved in toluene (5 mL) and a 2.5 M *n*BuLi solution in hexane (1.16 mL, 2.91 mmol, 2.1 eq.) was added dropwise at –78 °C. The reaction mixture was slowly warmed to room temperature and stirred at 80 °C overnight to obtain the dilithiated species **1** *in situ*. Then 9-bromo-9-borafluorene (707 mg, 2.91 mmol, 2.1 eq.) in toluene (5 mL) was added dropwise at –78 °C after which the reaction was slowly warmed to room temperature and stirred for 4 d. The suspension was filtered, the solid was washed with toluene (5 mL) and all volatiles were removed from the filtrate *in vacuo*. The crude product was recrystallized from toluene by hexane diffusion at –30 °C to give **3b** as orange crystals (85.0 mg, 182 $\mu$mol, 13%).

**$^1$H NMR** (500.1 MHz, $CD_2Cl_2$): $\delta$ = 7.65 (m, 1H, C$H_{Ar}$), 7.59 (m, 1H, C$H_{Ar}$), 7.56 (m, 1H, C$H_{Ar}$), 7.42 (m, 3H, C$H_{Ar}$), 7.36 (m, 1H, C$H_{Ar}$), 7.31 (m, 6H, C$H_{Ar}$), 7.25 (m, 1H, C$H_{Ar}$), 7.18 (m, 1H, C$H_{Ar}$), 7.16 (m, 1H, C$H_{Ar}$), 3.18–1.53 (br, 10H, B*H*) ppm.

**$^1$H{$^{11}$B} NMR** (500.1 MHz, $CD_2Cl_2$): $\delta$ = 7.65 (m, 1H, C$H_{Ar}$), 7.59 (m, 1H, C$H_{Ar}$), 7.56 (m, 1H, C$H_{Ar}$), 7.42 (m, 3H, C$H_{Ar}$), 7.36 (m, 1H, C$H_{Ar}$), 7.31 (m, 6H, C$H_{Ar}$), 7.25 (m, 1H, C$H_{Ar}$), 7.18 (m, 1H, C$H_{Ar}$), 7.16 (m, 1H, C$H_{Ar}$), 2.61(s, 1H, B*H*), 2.45–2.34 (m, 5H, B*H*), 2.27 (s, 1H, B*H*), 2.21(s, 1H, B*H*), 2.05(s, 1H, B*H*), 1.55(s, 1H, B*H*) ppm.

**$^{11}$B NMR** (160.5 MHz, $CD_2Cl_2$): $\delta$ = 66.1, 3.2, –4.4, –8.8 ppm.

**$^{11}$B{$^1$H} NMR** (160.5 MHz, $CD_2Cl_2$): $\delta$ = 66.1, 2.6, 2.2, –5.0, –5.7, –9.6 ppm.

**$^{13}$C{$^1$H} NMR** (125.8 MHz, $CD_2Cl_2$): $\delta$ = 155.8 ($C_{q,Ar}$), 152.9 ($C_{q,Ar}$), 143.9 ($C_{q,Ar}$), 143.8 ($C_{q,Ar}$), 138.6 ($C$H$_{Ar}$), 137.1 ($C$H$_{Ar}$), 136.4 ($C$H$_{Ar}$), 136.2 ($C$H$_{Ar}$), 129.9 ($C$H$_{Ar}$), 129.7 ($C$H$_{Ar}$), 129.6 ($C$H$_{Ar}$), 126.5 ($C$H$_{Ar}$), 129.1 ($C$H$_{Ar}$), 129.0 ($C$H$_{Ar}$), 128.1 ($C$H$_{Ar}$), 126.1 ($C$H$_{Ar}$), 125.7 ($C$H$_{Ar}$), 121.7 ($C$H$_{Ar}$) ppm.

**HRMS LIFDI** calc. for $[C_{26}H_{26}B_{12}]^+$ = $[M]^+$: 468.3219, found 468.3218.

**5-([1,1'-biphenyl]-2-yl)-5H-benzo[4,5]borolo[3,2,1-de]boranthrene (3b)**

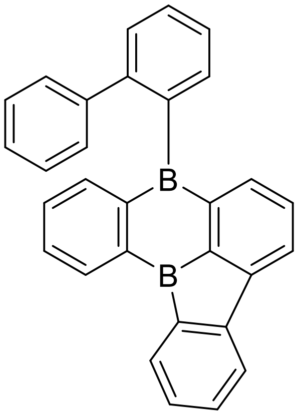

Compound **2b** (37.0 mg, 92.0 *µ*mol) was heated for 3 d at 120 °C in toluene. Removal of all volatiles *in vacuo* led to the isolation of **3b** as an orange solid (34.0 mg, 84.5 *µ*mol, 92%).

**$^{1}$H NMR** (500.1 MHz, $CD_2Cl_2$): *δ* = 8.33 (m, 1H, C$H_{Ar}$), 8.02 (m, 1H, C$H_{Ar}$), 7.73 (m, 1H, C$H_{Ar}$), 7.67 (m, 1H, C$H_{Ar}$), 7.58 (m, 1H, C$H_{Ar}$), 7.55 (m, 1H, C$H_{Ar}$) 7.48 (m, 3H, C$H_{Ar}$), 7.42 (m, 1H, C$H_{Ar}$), 7.39 (m, 1H, C$H_{Ar}$), 7.35 (m, 3H, C$H_{Ar}$), 7.20 (m, 1H, C$H_{Ar}$), 7.13 (m, 4H, C$H_{Ar}$), 6.98 (m, 1H, C$H_{Ar}$) ppm.
**$^{11}$B NMR** (160.5 MHz, $CD_2Cl_2$): *δ* = 63.9 (br) ppm.
**$^{13}$C{$^{1}$H} NMR** (125.8 MHz, $CD_2Cl_2$): *δ* = 156.80 ($C_{q,Ar}$), 153.26 ($C_{q,Ar}$), 150.80 ($C_{q,Ar}$), 149.68 ($C_{q,Ar}$), 145.62 ($C_{q,Ar}$), 144.32 ($C_{q,Ar}$), 144.11 ($C_{q,Ar}$), 143.36 ($C_{q,Ar}$), 141.99 ($C_{q,Ar}$), 141.12 ($C$H$_{Ar}$), 137.49 ($C$H$_{Ar}$), 135.79 ($C$H$_{Ar}$), 135.60 ($C$H$_{Ar}$), 134.72 ($C$H$_{Ar}$), 133.97 ($C$H$_{Ar}$), 133.95 ($C$H$_{Ar}$), 132.18 ($C$H$_{Ar}$), 131.51 ($C$H$_{Ar}$), 129.08 ($C$H$_{Ar}$), 128.96 ($C$H$_{Ar}$), 128.93 ($C$H$_{Ar}$), 128.50 ($C$H$_{Ar}$), 128.44 ($C$H$_{Ar}$), 127.52 ($C$H$_{Ar}$), 126.46 ($C$H$_{Ar}$), 123.40 ($C$H$_{Ar}$), 121.14 ($C$H$_{Ar}$), ppm.
**HRMS LIFDI** calc. for $[C_{30}H_{20}B_2]^+$ = $[M]^+$: 402.1746, found 402.1743.

**Synthesis of 5**

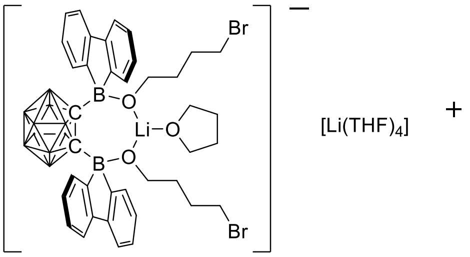


The compound 9-Br-9-borafluorene (10 mg, 41.2 $\mu$mol, 2.5 eq) was suspended in THF-$d_8$ (1 mL) and the mixture was stirred at 60 °C for 1 h. The reaction was monitored by $^{11}$B NMR spectroscopy. Then, 1,2-$Li_2$-1,2-$C_2B_{10}H_{10}$·$(Et_2O)_2$ (5 mg, 16.4 $\mu$mol, 1.0 eq) was added at –30 °C and the reaction was allowed to warm to room temperature. The reaction was monitored by $^{1}$H and $^{11}$B NMR spectroscopy. Crystals of **5** were obtained from the reaction solution by pentane diffusion at –30 °C.

**$^{1}$H NMR** (300.2 MHz, THF-$d_8$): $\delta$ = 7.82 (m, 2H, C$H_{Ar}$), 7.39 (m, 2H, C$H_{Ar}$), 6.96 (m, 4H, C$H_{Ar}$) ppm.

**$^{11}$B{$^{1}$H} NMR** (96.3 MHz, THF-$d_8$): $\delta$ = 4.1, –3.2, –6.7, –8.4 ppm.

**$^{13}$C{$^{1}$H} NMR** (75.5 MHz, THF-$d_8$): $\delta$ = 149.9 ($C_{q,Ar}$), 133.5 ($C$H$_{Ar}$), 125.6 ($C$H$_{Ar}$), 124.9 ($C$H$_{Ar}$), 117.9 ($C$H$_{Ar}$) ppm.

**Synthesis of 9-(4-bromobutoxy)-9-borafluorene**

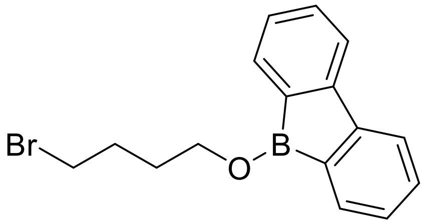


The compound 9-Br-9-borafluorene (10 mg, 41.2 $\mu$mol) was suspended in THF (1 mL). The reaction mixture was stirred at 60 °C for 1 h and was monitored by $^{11}$B NMR spectroscopy. Removal of all volatiles *in vacuo* led to the isolation of 9-(4-bromobutoxy)-9-borafluorene. Crystals of the product were obtained from a $CD_2Cl_2$ solution by pentane diffusion at –30 °C.

**$^{1}$H NMR** (500.1 MHz, $CD_2Cl_2$): $\delta$ = 7.55 (m, 2H, C$H_{Ar}$), 7.49 (m, 2H, C$H_{Ar}$), 7.35 (m, 2H, C$H_{Ar}$), 7.19 (m, 2H, C$H_{Ar}$), 4.61 (t, $^{3}J_{HH}$ = 6.24 Hz, 2H, C$H_2$), 3.54 (t, $^{3}J_{HH}$ = 6.59 Hz, 2H, C$H_2$), 2.11 (m, 2H, C$H_2$), 2.00 (m, 2H, C$H_2$) ppm.

**$^{11}$B NMR** (160.5 MHz, $CD_2Cl_2$): $\delta$ = 45.0 ppm.

**$^{13}$C{$^{1}$H} NMR** (125.8 MHz, $CD_2Cl_2$): $\delta$ = 152.8 ($C_{q,Ar}$), 132.9 ($C$H$_{Ar}$), 132.3 ($C$H$_{Ar}$), 128.2 ($C$H$_{Ar}$), 120.0 ($C$H$_{Ar}$), 68.0 ($C$H$_2$), 34.2 ($C$H$_2$), 30.7 ($C$H$_2$), 29.8 ($C$H$_2$) ppm.

**HRMS LIFDI** calc. for $[C_{16}H_{16}B_1O_1]^+$ = $[M]^+$: 314.0472, found 314.0463.

**Synthesis of 9-($Me_2S$)-9-Br-9-borafluorene**

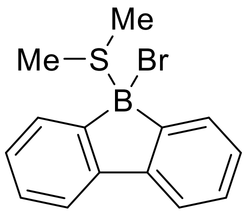


The compound 9-Br-9-borafluorene (10 mg, 41.2 $\mu$mol) was dissolved in toluene (1 mL), one drop of $Me_2S$ was added, and a colorless solid precipitated from the solution. The reaction mixture was stirred at room temperature overnight. Removal of all volatiles *in vacuo* led to the formation of the $Me_2S$ adduct in quantitative yield according to $^{1}$H and $^{11}$B NMR spectroscopy. Crystals of the product were obtained from a $Me_2S$ solution by pentane diffusion at –30 °C.

**$^{1}$H NMR** (500.1 MHz, $CD_2Cl_2$): $\delta$ = 7.64 (m, 2H, $CH_{Ar}$), 7.54 (m, 2H, $CH_{Ar}$), 7.33 (m, 2H, $CH_{Ar}$), 7.23 (m, 2H, $CH_{Ar}$), 2.18 (s, 6H, $CH_3$) ppm.

**$^{11}$B NMR** (160.5 MHz, $CD_2Cl_2$): $\delta$ = 0.3 ppm.

**$^{13}$C{$^{1}$H} NMR** (125.8 MHz, $CD_2Cl_2$): $\delta$ = 148.5 ($C_{q,Ar}$), 131.2 ($C$H$_{Ar}$), 129.4 ($C$H$_{Ar}$), 127.6 ($C$H$_{Ar}$), 120.1 ($C$H$_{Ar}$), 20.7 ($C$H$_3$) ppm.

$^{1}$H NMR spectrum of 2b in $CD_2Cl_2$

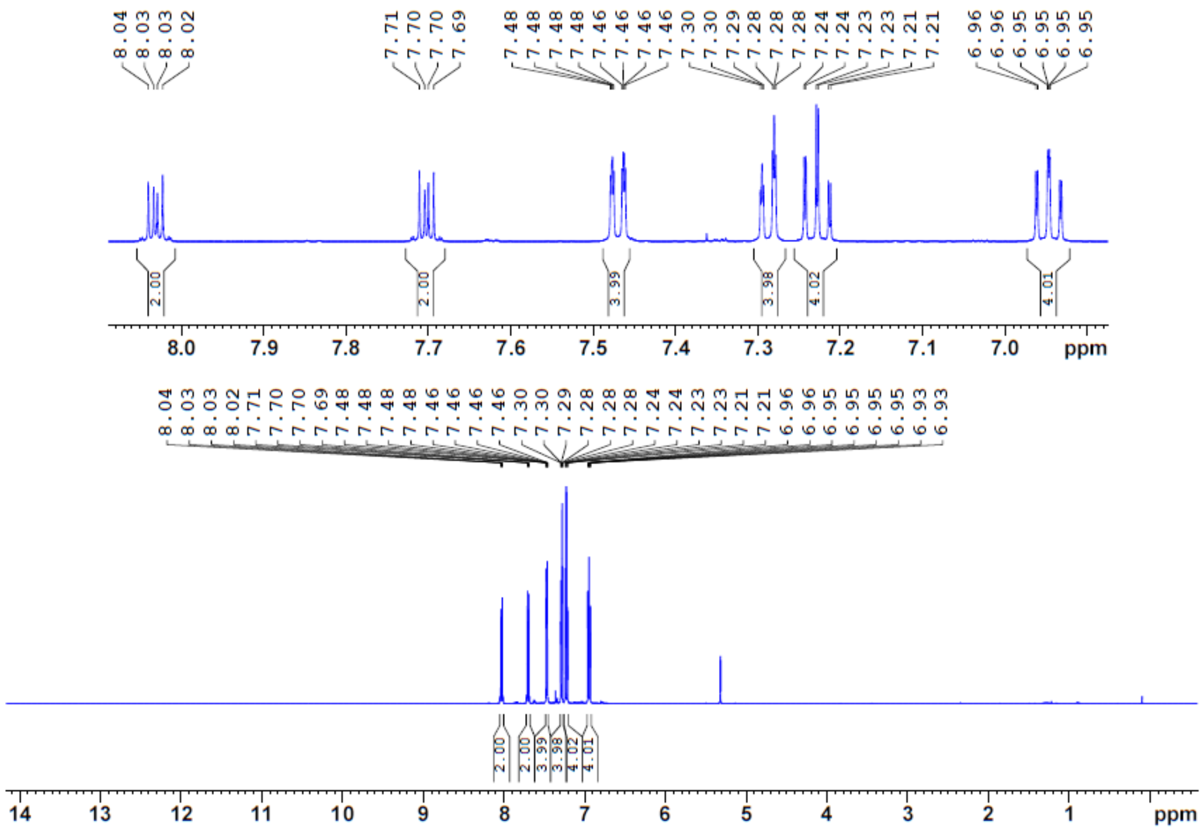

$^{11}$B NMR spectrum of 2b in $CD_2Cl_2$

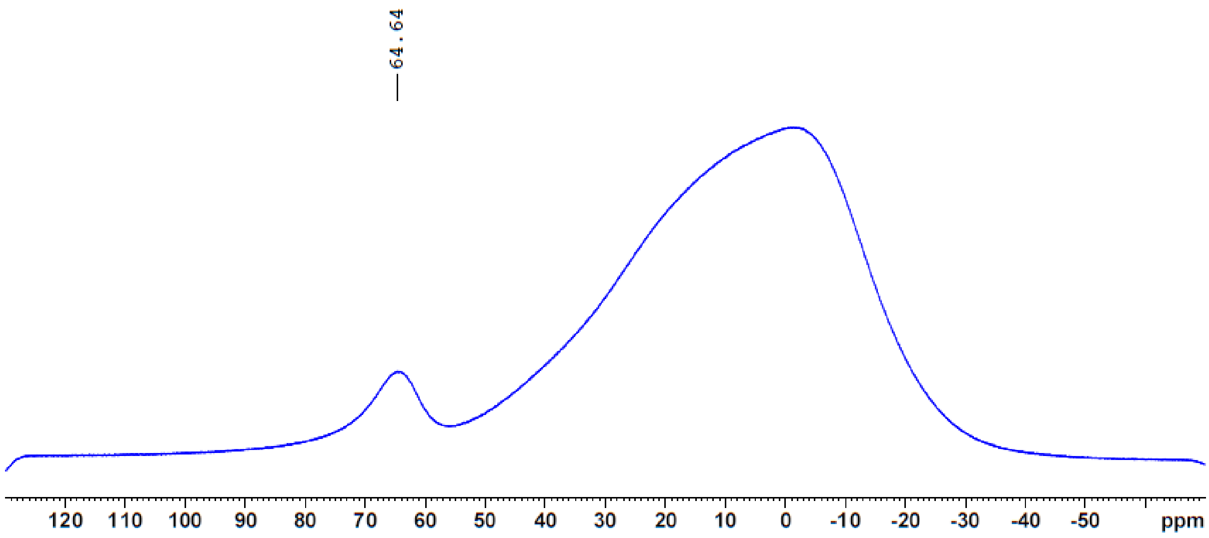

**$^{13}C\{^{1}H\}$ NMR spectrum of 2b in $CD_2Cl_2$**

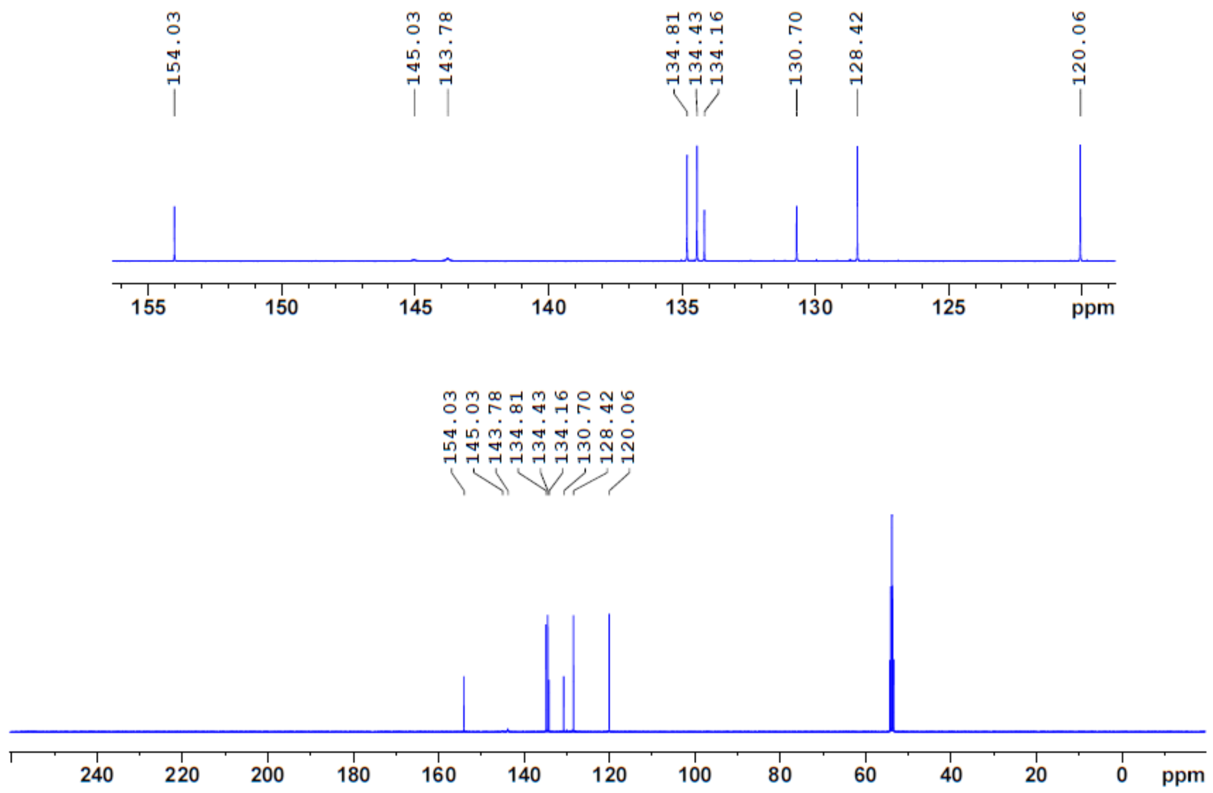


**$^{1}H$ NMR spectrum of 3a in $CD_2Cl_2$**

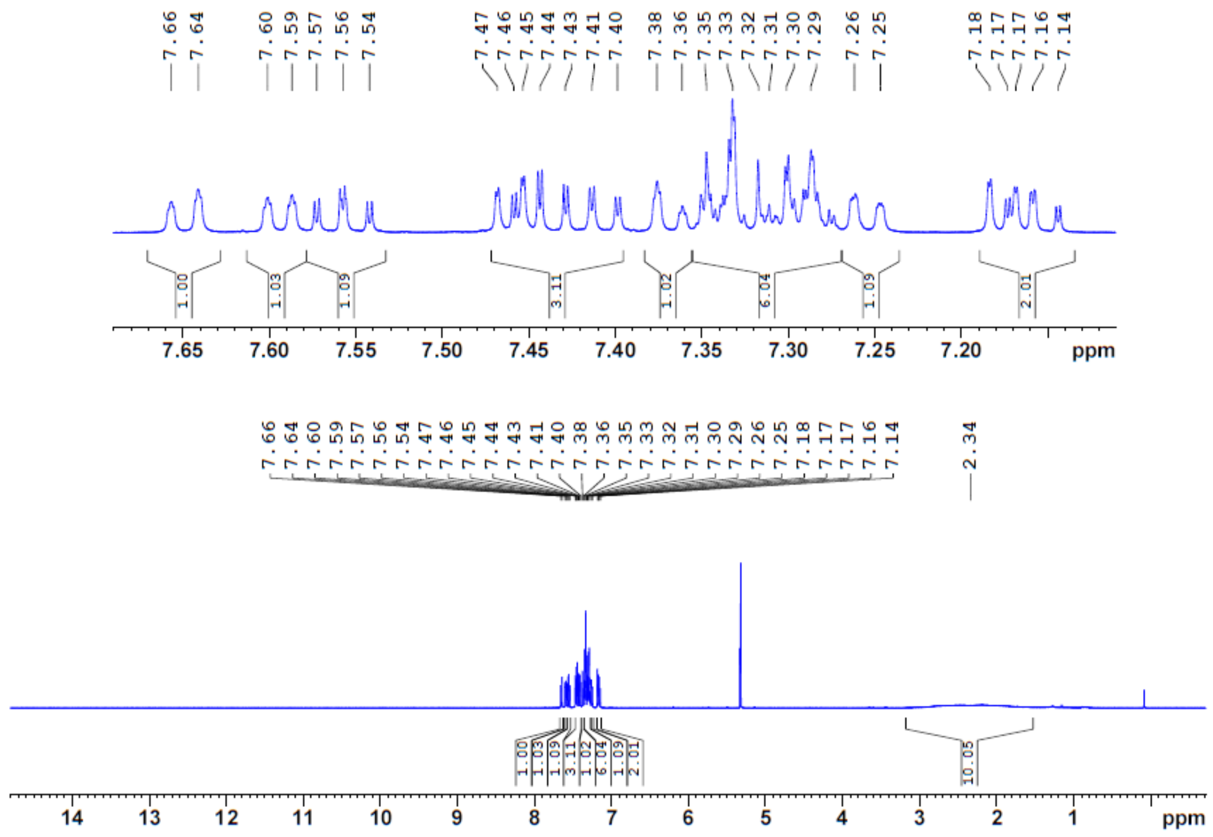

**$^{1}H$ {$^{11}B$} NMR spectrum of 3a in $CD_2Cl_2$**

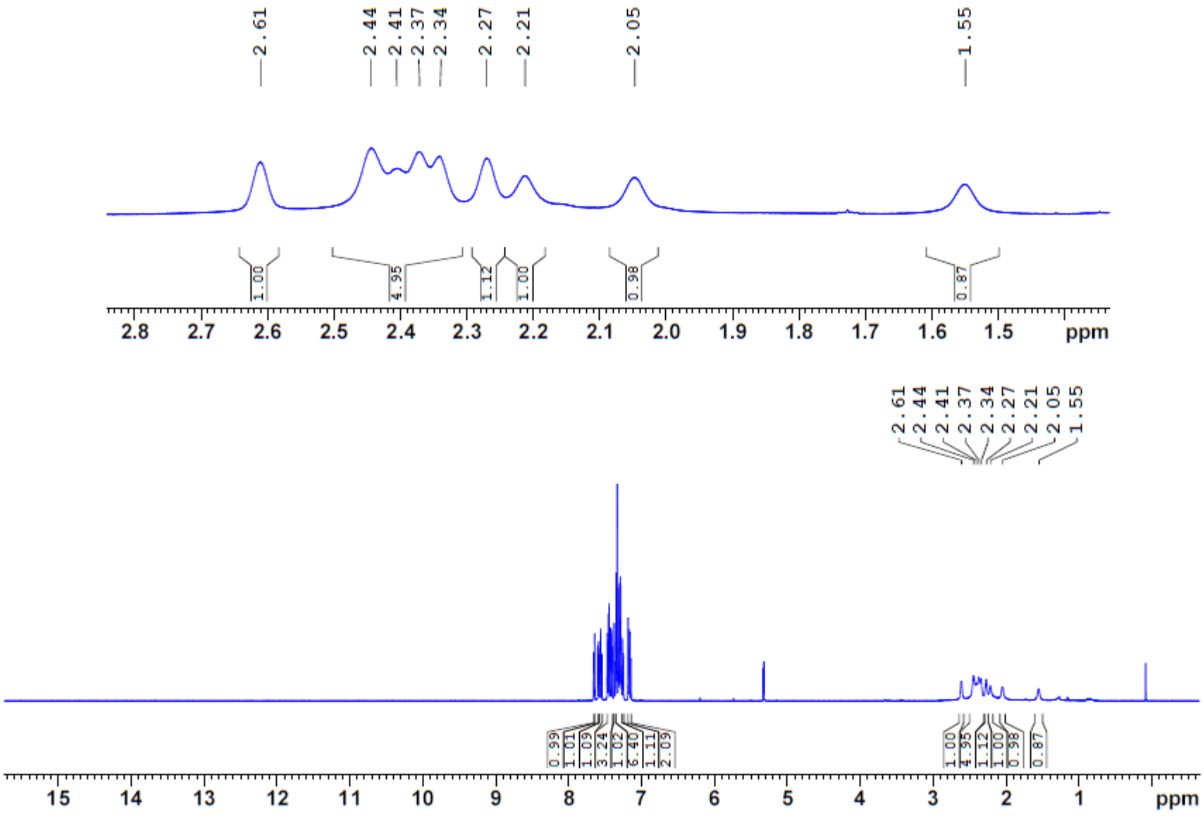

$^{11}B$ NMR spectrum of 3a in $CD_2Cl_2$

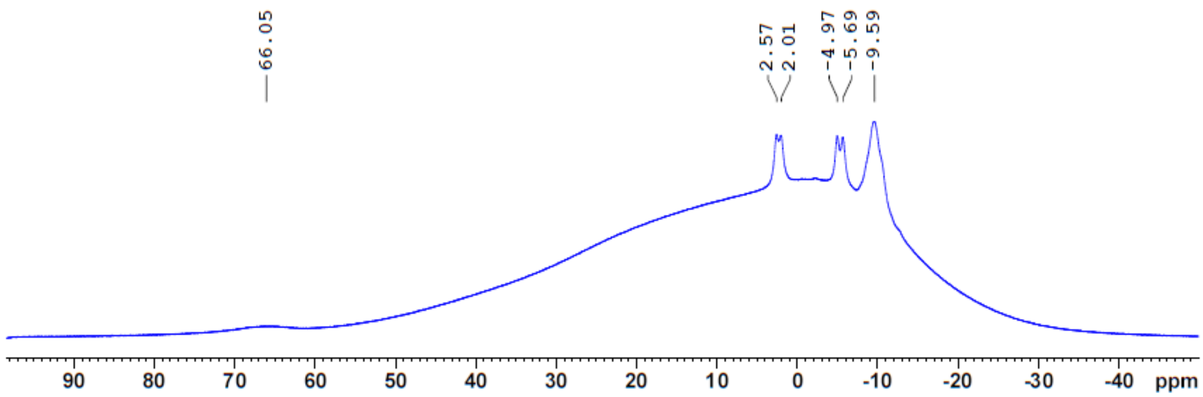


$^{11}B\{^1H\}$ NMR spectrum of 3a in $CD_2Cl_2$

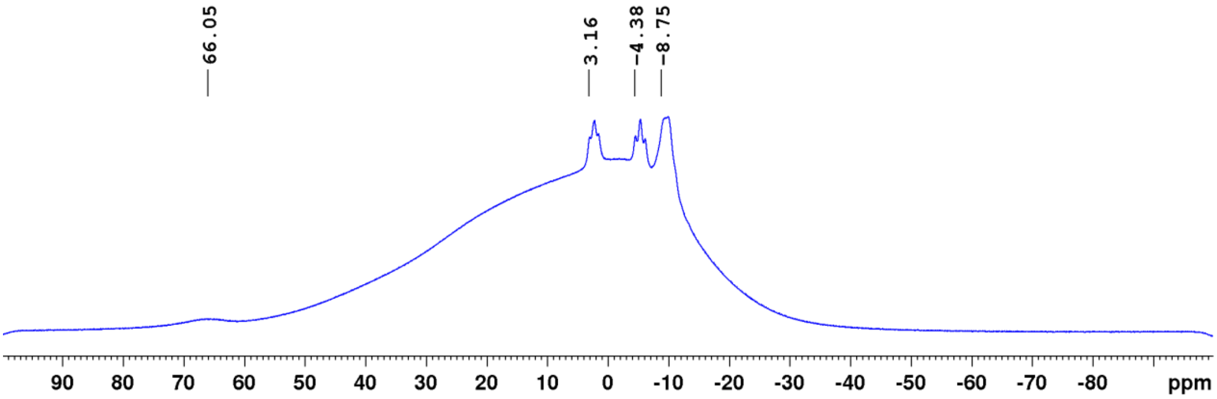


$^{13}C\{^1H\}$ NMR spectrum of 3a in $CD_2Cl_2$

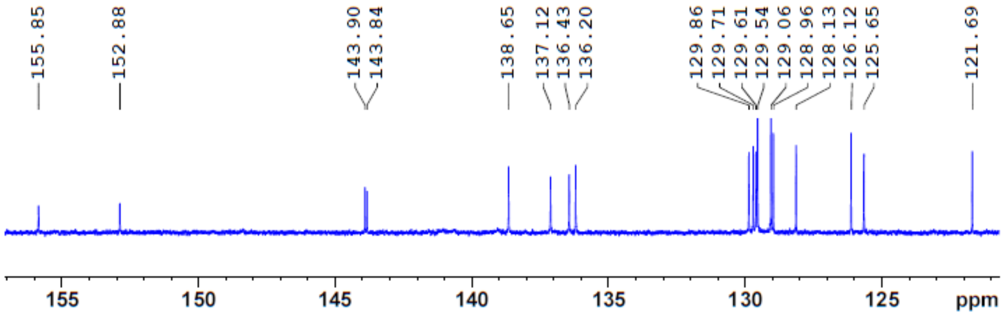


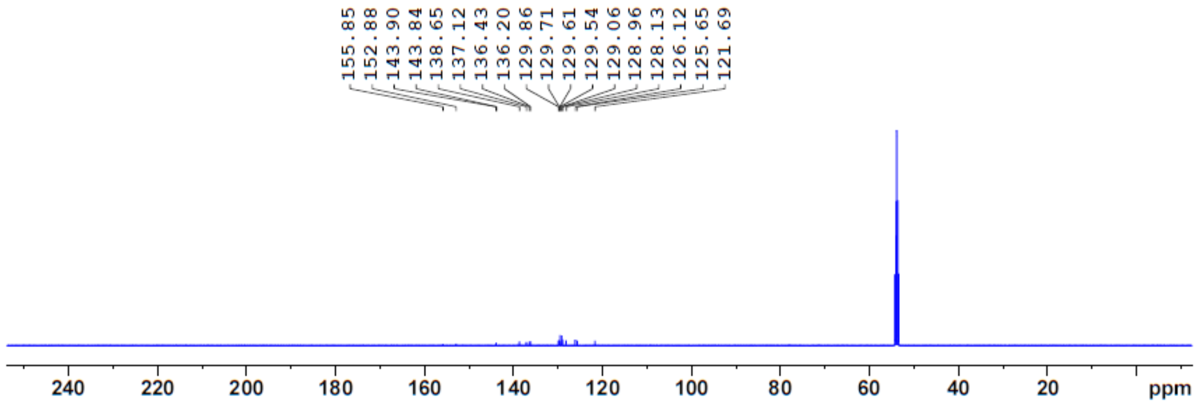

$^{1}$H NMR spectrum of 3b in $CD_2Cl_2$

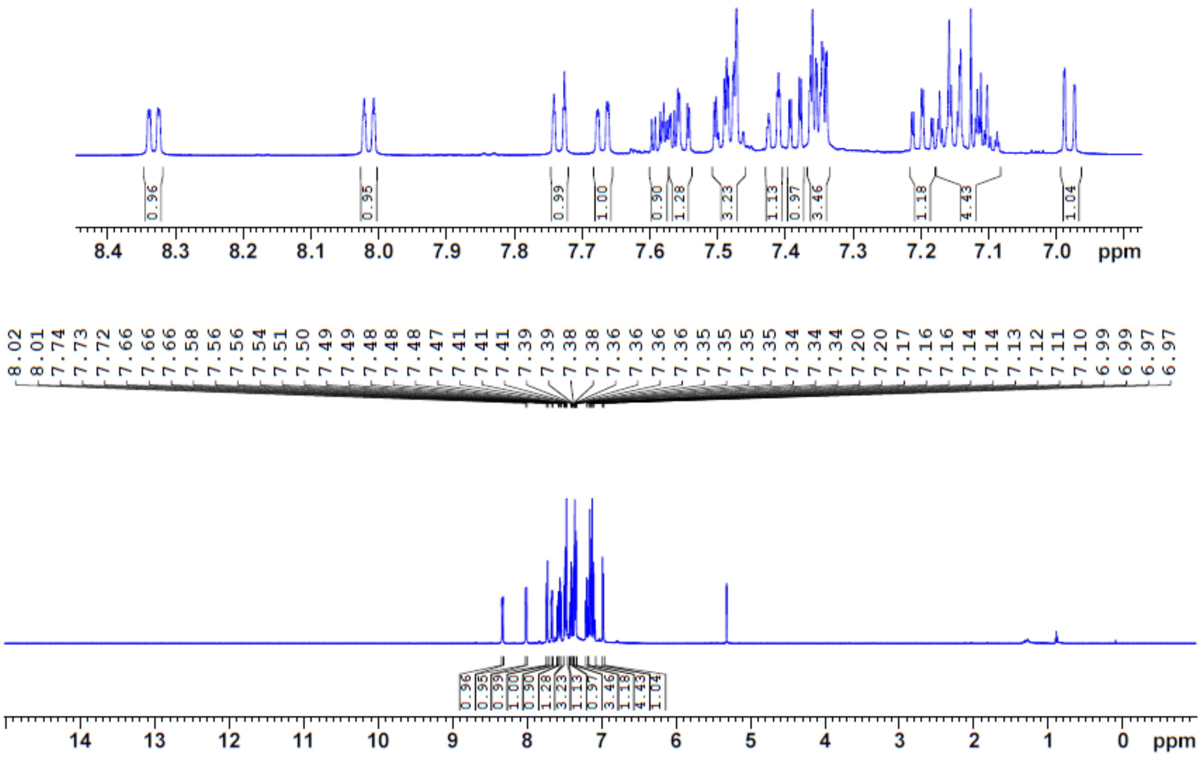


$^{11}$B NMR spectrum of 3b in $CD_2Cl_2$

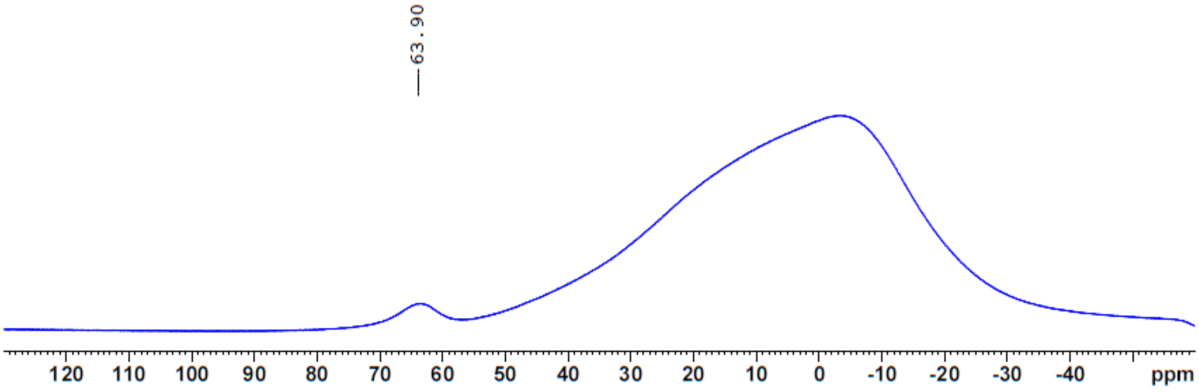

**$^{13}$C{$^{1}$H} NMR spectrum of 3b in $CD_2Cl_2$**

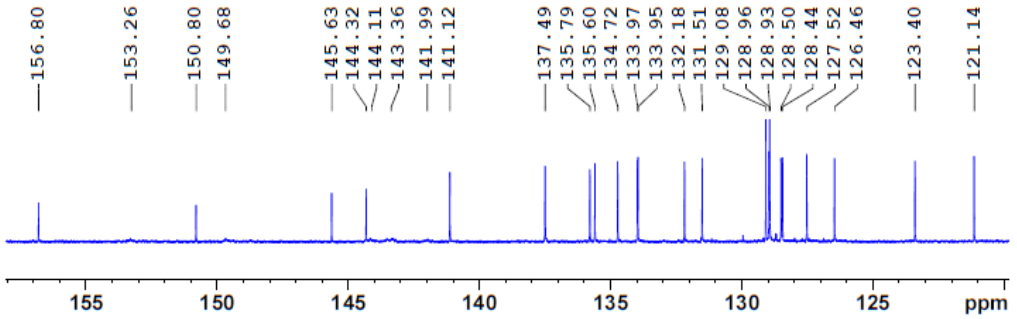


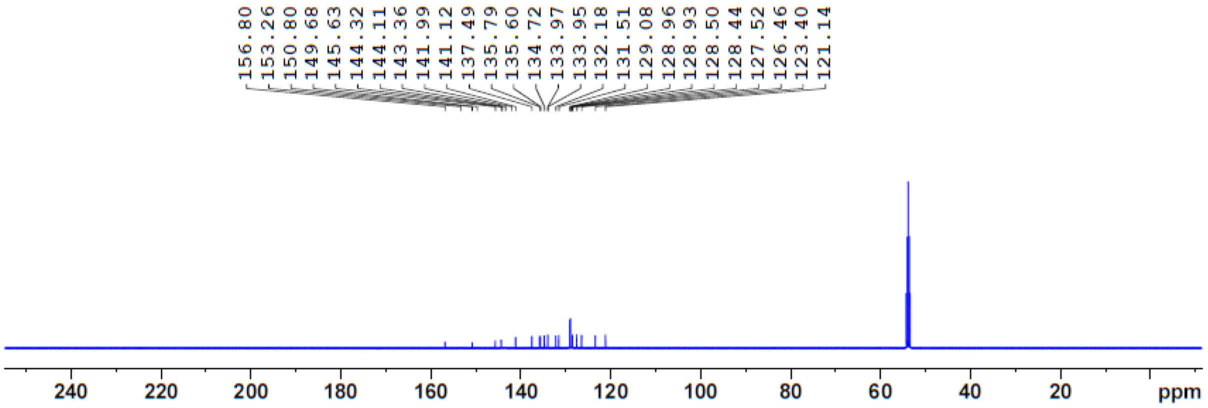


**$^{1}$H NMR spectrum of 9-(4-bromobutoxy)-9-borafluorene in $CD_2Cl_2$**

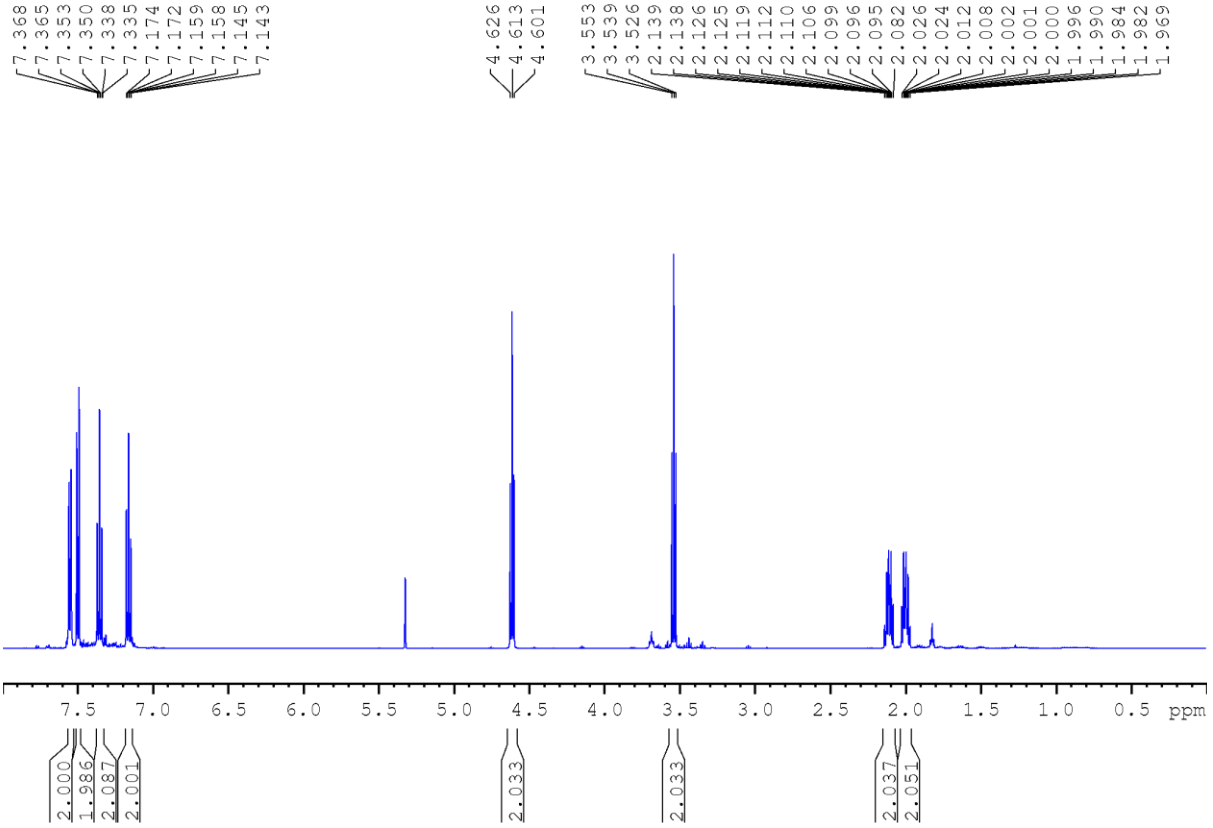

**$^{11}$B NMR spectrum of 9-(4-bromobutoxy)-9-borafluorene in $CD_2Cl_2$**

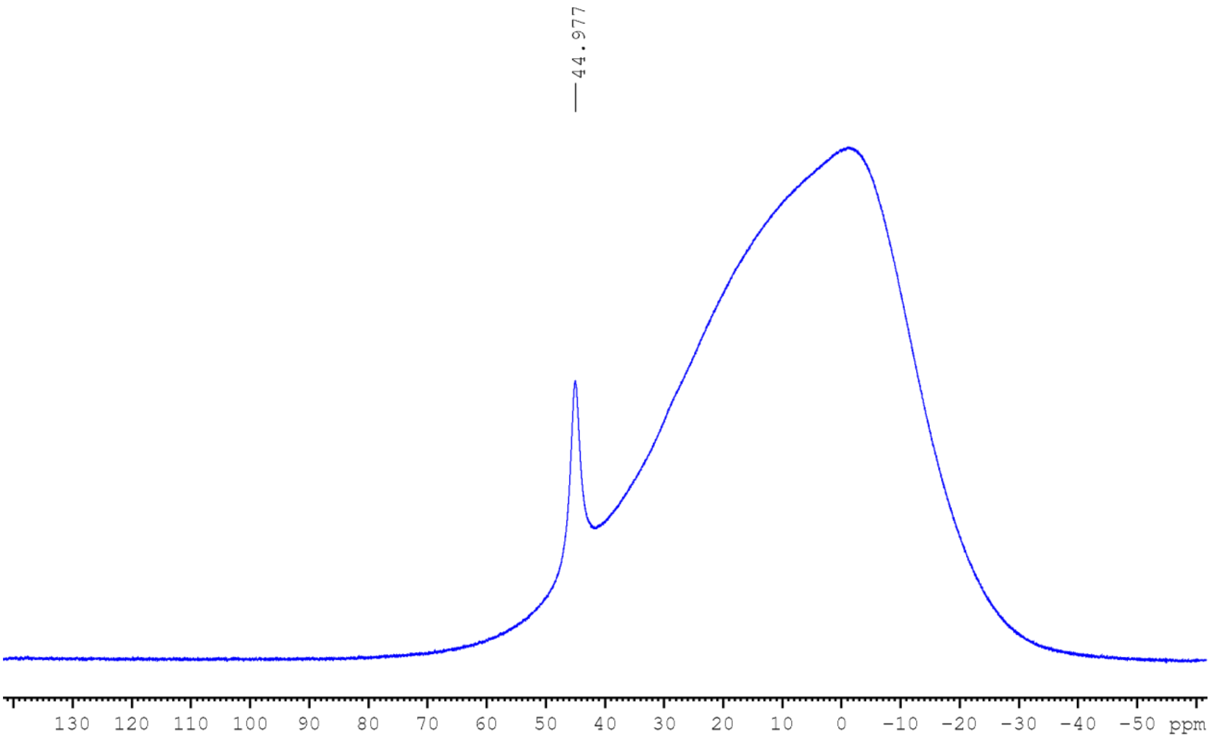


**$^{13}$C{$^{1}$H} NMR spectrum of 9-(4-bromobutoxy)-9-borafluorene in $CD_2Cl_2$**

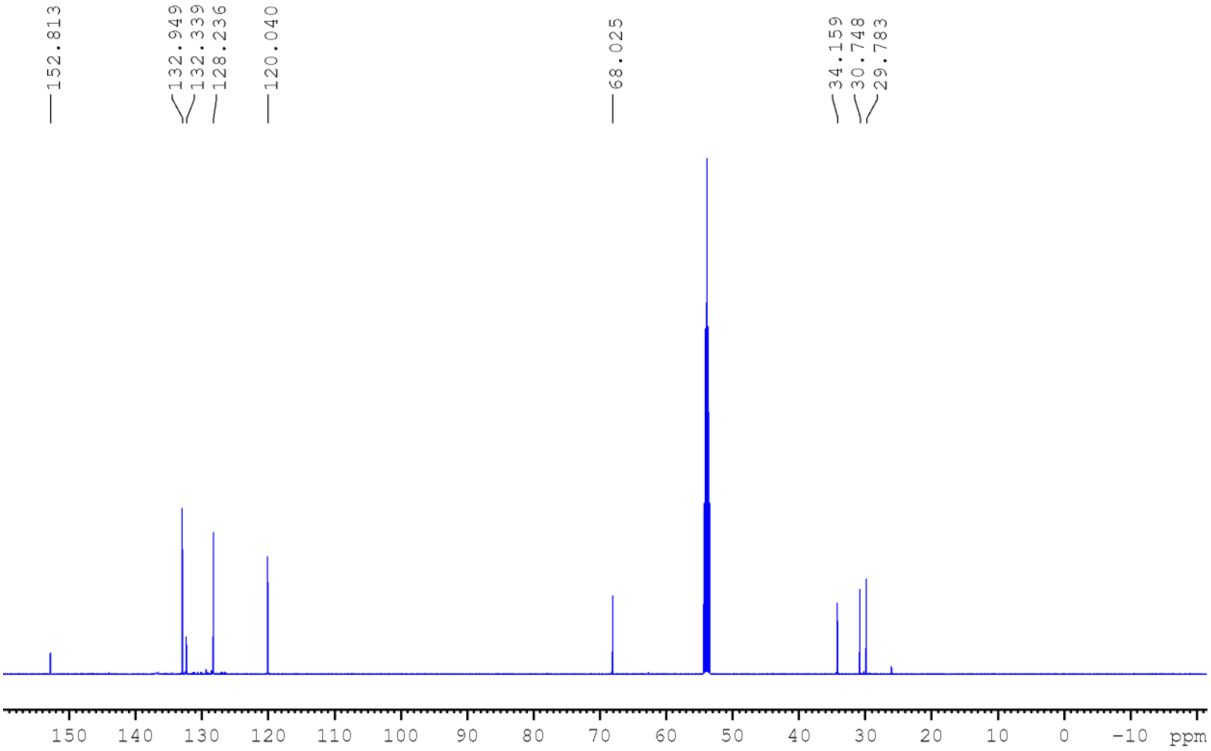

**$^{1}$H NMR spectrum of the 9-($Me_2S$)-9-Br-9-borafluorene in $CD_2Cl_2$**

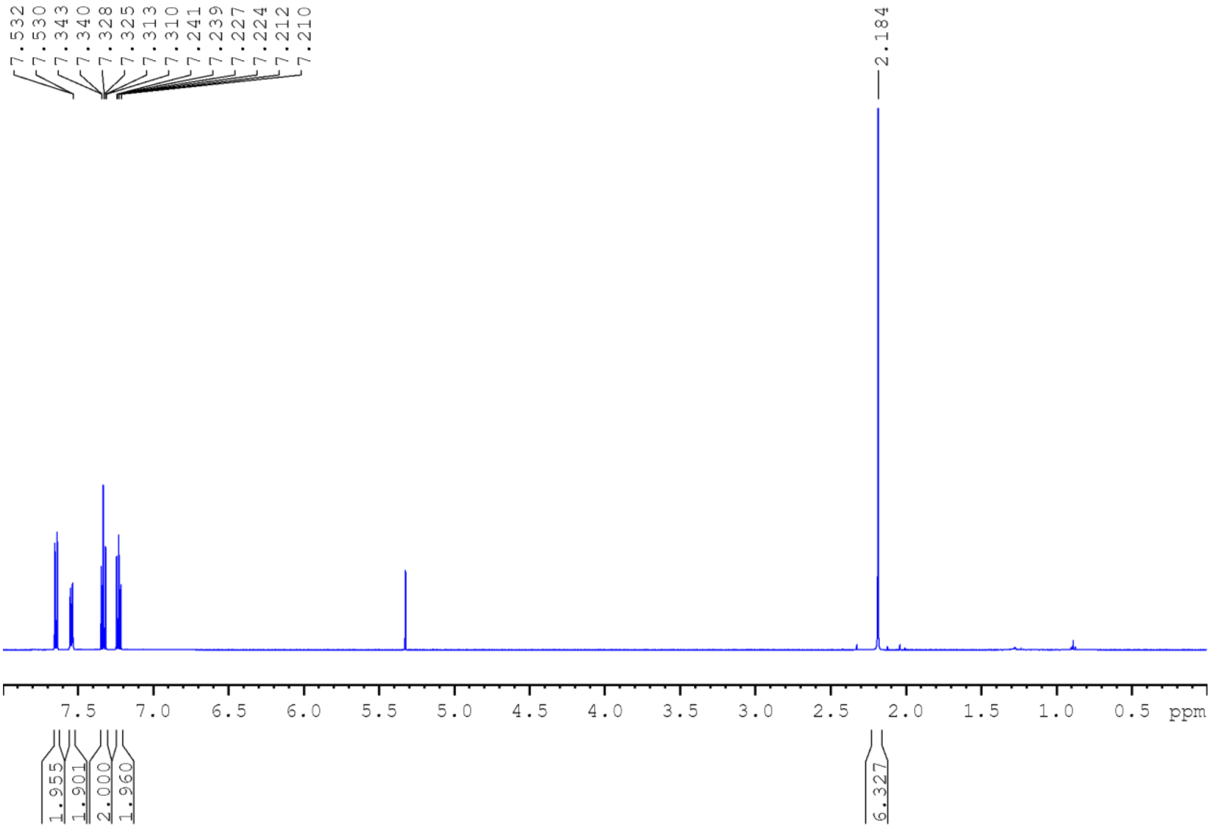


**$^{11}$B NMR spectrum of the 9-($Me_2S$)-9-Br-9-borafluorene in $CD_2Cl_2$**

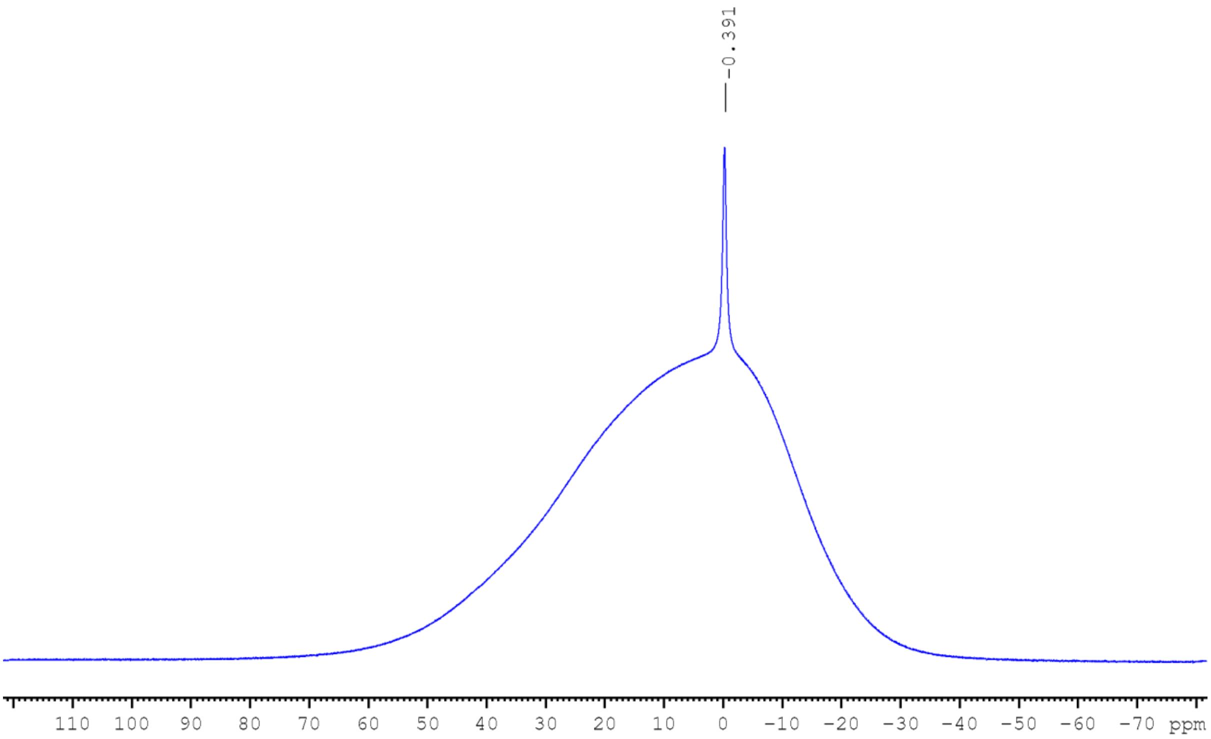

**$^{13}C\{^1H\}$ NMR spectrum of the 9-($Me_2S$)-9-Br-9-borafluorene in $CD_2Cl_2$**

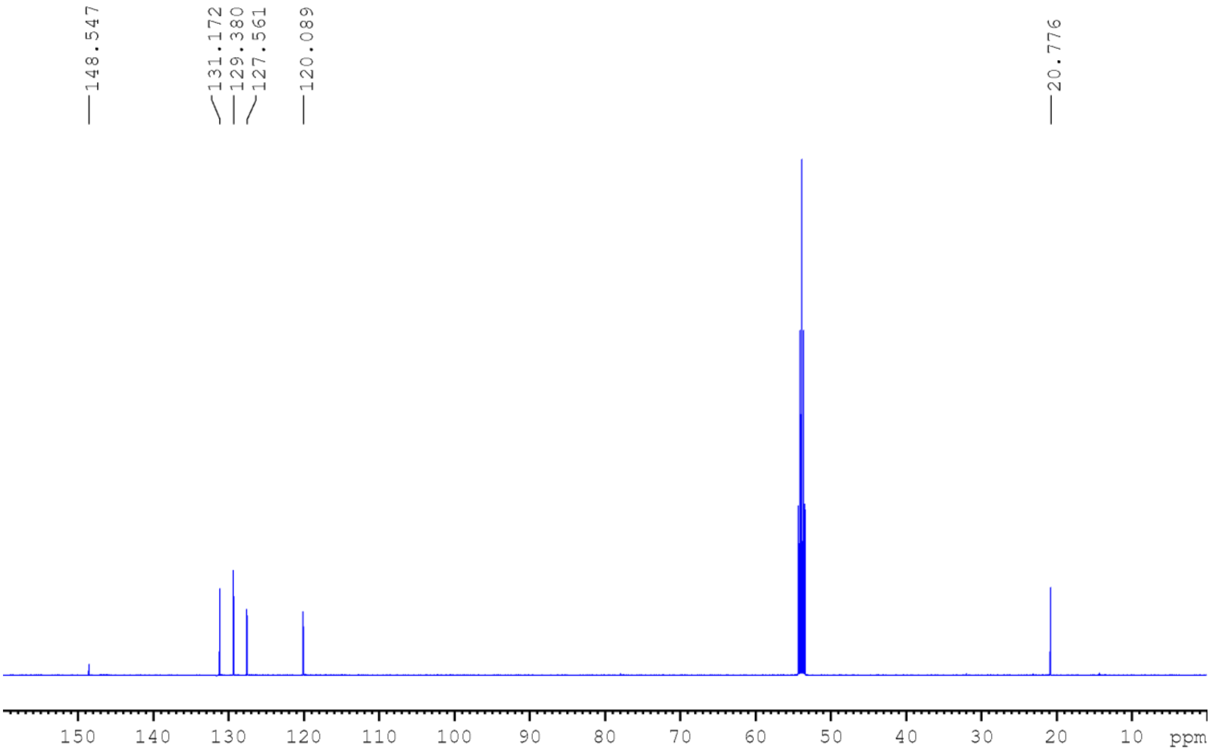

**Additional reactions**

A clean reaction was observed for the synthesis of **5** when the reaction was carried out in THF-$d_8$ as a one pot synthesis with an excess of **9-Br-9-borafluorene**, with the formation of a single new species by NMR spectroscopy (see below). As the ring-opened species arises from the deuterated THF solvent, the alkyl chain was not observed in the $^{1}H$ and $^{13}C$ NMR spectrum. Compound **5** was not detected by HRMS (LIFDI) as it is an anion.

When this reaction was repeated in non-deuterated THF, removal of all volatiles *in vacuo* after the formation of 9-(4-bromobutoxy)-9-borafluorene led to the formation of some impurities. Continuing the reaction in THF-$d_8$ resulted in a mixture of compounds in the $^{1}H$ and $^{11}B$ NMR spectra.

The following NMR spectra show a comparison of the deuterated and non-deuterated 9-(4-bromobutoxy)-9-borafluorene and the reaction of the deuterated 9-(4-bromobutoxy)-9-borafluorene monitored by $^{1}H$ and $^{11}B$ NMR spectra.

**$^{1}$H NMR spectrum (300.2 MHz, THF-$d_8$):** Comparison of the deuterated (blue) and non-deuterated (red) **9-(4-bromobutoxy)-9-borafluorene**.

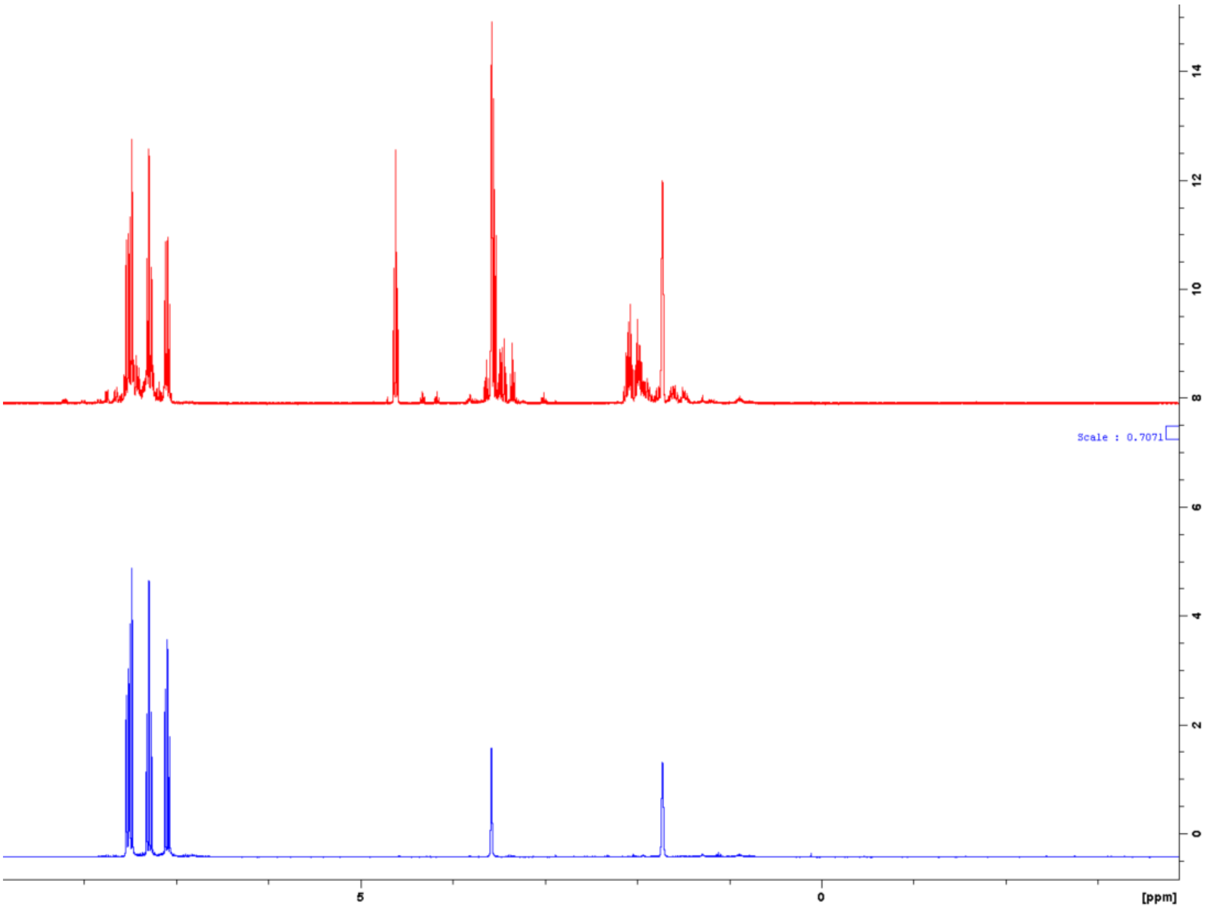


**$^{1}$H NMR spectrum (300.2 MHz, THF-$d_8$):** Reaction before (blue) and after (red) the addition of 1,2-$Li_2$-1,2-$C_2B_{10}H_{10}$·$(Et_2O)_2$.

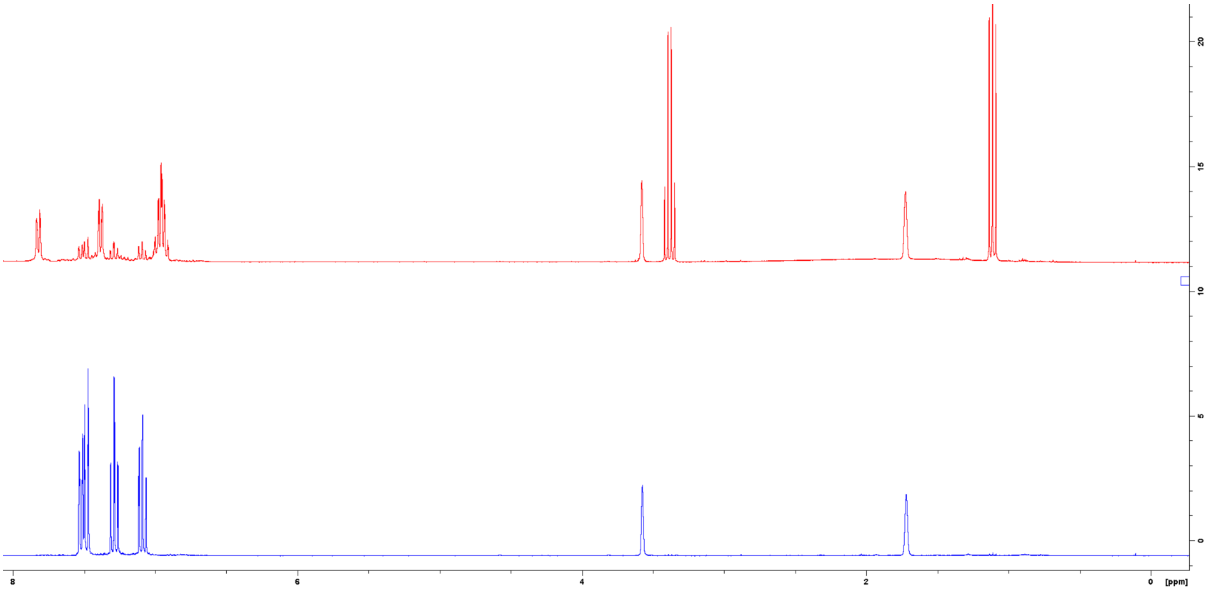

**$^{13}C\{^1H\}$ NMR spectrum (75.5 MHz, THF-d$_8$):** Reaction before (blue) and after (red) the addition of 1,2-$Li_2$-1,2-$C_2B_{10}H_{10}$·$(Et_2O)_2$.

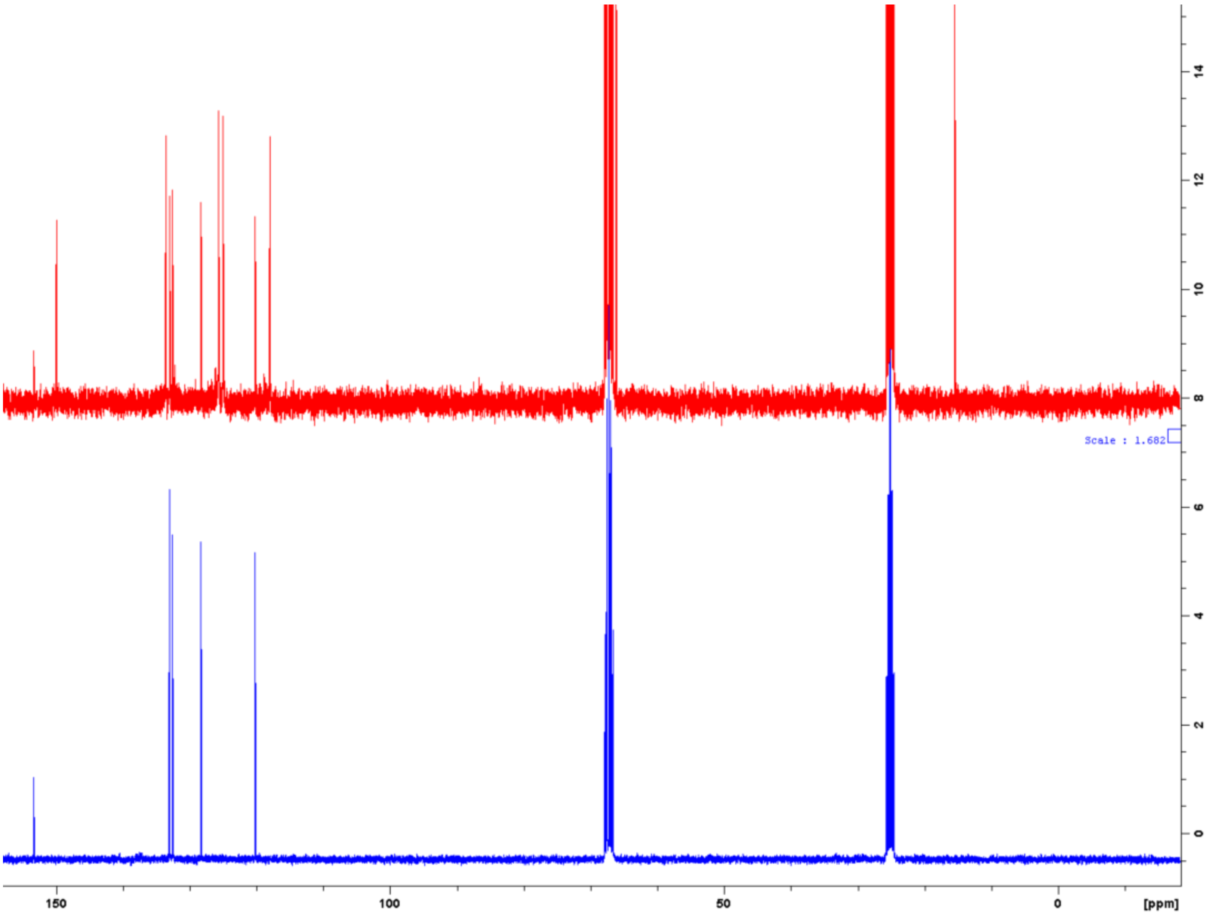


**$^{11}B\{^1H\}$ NMR spectrum (96.3 MHz, THF-d$_8$):** Reaction before (blue) and after (red) the addition of 1,2-$Li_2$-1,2-$C_2B_{10}H_{10}$·$(Et_2O)_2$.

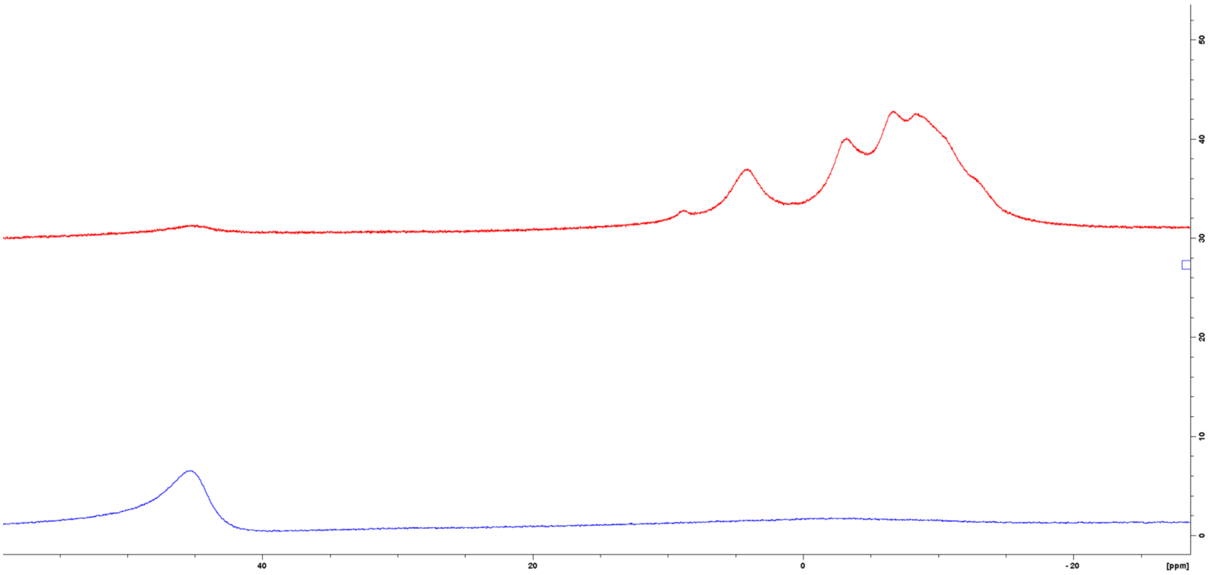

**$^{1}$H NMR spectrum (300.2 MHz, THF-d$_8$):** Difference between the reaction of (impure) non-deuterated **9-(4-bromobutoxy)-9-borafluorene** (red) and deuterated **9-(4-bromobutoxy)-9-borafluorene** (blue) after the addition of 1,2-$Li_2$-1,2-$C_2B_{10}H_{10}$·$(Et_2O)_2$.

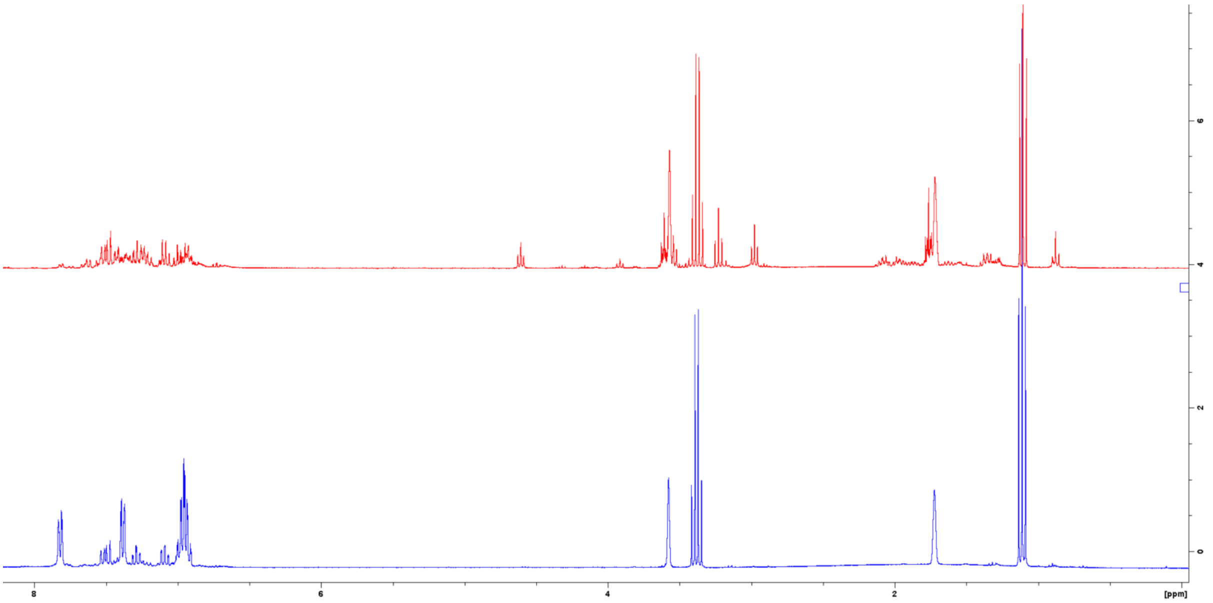

When **9-($Me_2S$)-9-Br-9-borafluorene** was reacted with 1,2-$Li_2$-1,2-$C_2B_{10}H_{10}$·($Et_2O$)$_2$ in $C_6D_6$, solubility was low and the reaction led to a complex mixture, as observed by NMR spectroscopy and HRMS. When the same reaction was carried out in $Me_2S$, reaction between the solvent and 1,2-$Li_2$-1,2-$C_2B_{10}H_{10}$·($Et_2O$)$_2$ formed a complex mixture from which a crystal of **1-MeS-2-($Me_2S$-9-borafluorene)-1,2-$C_2B_{10}H_{10}$** was isolated and characterized by single-crystal X-ray diffraction. To confirm the reaction between the solvent and dilithium salt, as similar reactions are known for *n*-butyllithium,[25] 1,2-$Li_2$-1,2-$C_2B_{10}H_{10}$·($Et_2O$)$_2$ was dissolved in $Me_2S$. In this experiment **1-MeS-1,2-$C_2B_{10}H_{11}$** (calc. for [$C_3H_{14}B_{10}S$] = [M]: 190.2, found 190.1), **1,2-(MeS)$_2$-1,2-$C_2B_{10}H_{10}$** (calc. for [$C_4H_{16}B_{10}S_2$] = [M]: 236.2, found 236.1) were visible in GCMS, and **μ-1,2-$CH_2$-(1,2-$C_2B_{10}H_{11}$)** (calc. for [$C_5H_{24}B_{20}$]$^-$ = [M]$^-$: 300.3884, found 300.3889), as well as compounds of higher mass were visible in the HRMS.

HRMS (APCI neg) of the reaction of $Me_2S$ with 1,2-$Li_2$-1,2-$C_2B_{10}H_{10}$($Et_2O$)$_2$

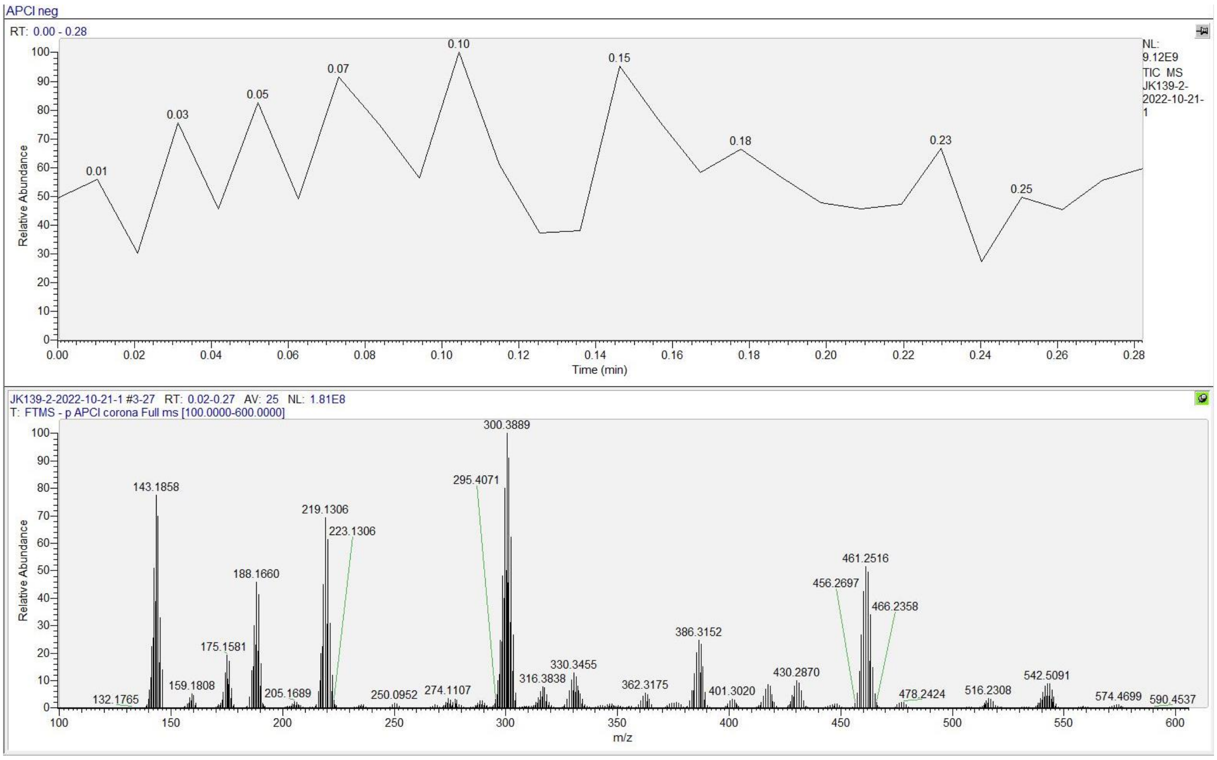

GCMS of the reaction of $Me_2S$ with 1,2-$Li_2$-1,2-$C_2B_{10}H_{10}(Et_2O)_2$

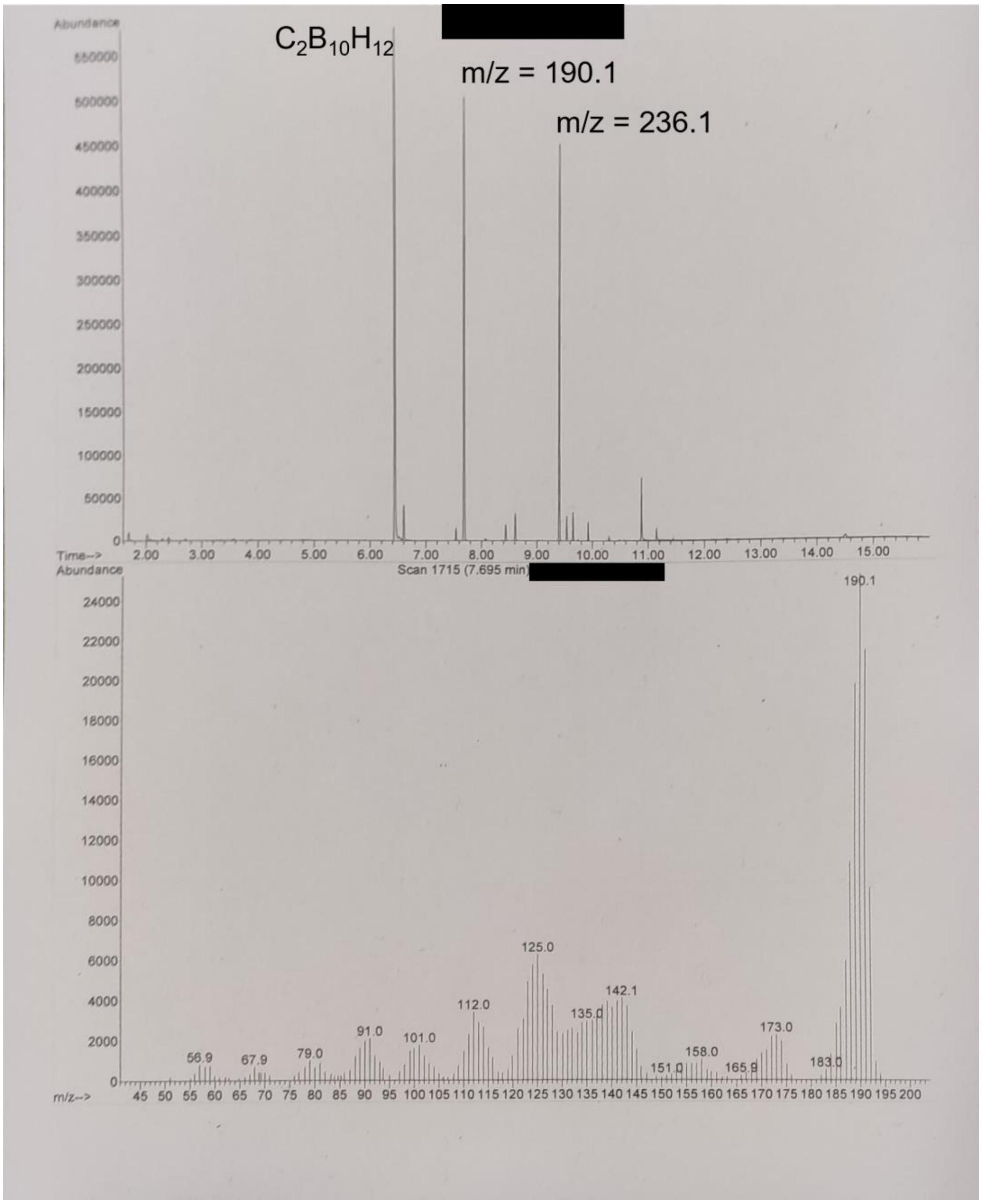

# Single-crystal X-ray diffraction

Table S1. Single-crystal X-ray diffraction data and structure refinements of **2b**, **3a**, **3b**, **3a·THF**, **5**, **9-($Me_2S$)-9-Br-9-borafluorene**, **1-MeS-2-($Me_2S$-9-borafluorene)-1,2-$C_2B_{10}H_{10}$**, and **9-(4-bromobutoxy)-9-borafluorene**.

| | **2b** | **3a** | **3b** | **3a·THF** |
|---|---|---|---|---|
| CCDC | 2174245 | 2174247 | 2174246 | 2174248 |
| Empirical formula | $C_{30}H_{20}B_2$ | $C_{26}H_{26}B_{12}$ | $C_{30}H_{20}B_2$ [+solvent] | 2($C_{30}$ $H_{34}$ $B_{12}$ O), $C_7$ $H_8$ |
| $\rho_{calc}$/g·cm$^{-3}$ | 1.260 | 1.213 | 1.065 | 1.207 |
| $F$(000) | 840 | 968 | 1680 | 614 |
| Crystal size/mm$^3$ | 0.22×0.20×0.16 | 0.68×0.32×0.27 | 0.15×0.04×0.02 | 0.31×0.18×0.13 |
| Crystal colour, habit | yellow block | orange block | orange block | colourless block |
| $\mu$/mm$^{-1}$ | 0.070 | 0.062 | 0.445 | 0.476 |
| $M_r$/g·mol$^{-1}$ | 402.08 | 468.19 | 402.08 | 1172.71 |
| Temperature/K | 100(2) | 100(2) | 100(2) | 100(2) |
| Radiation, λ/Å | $MoK_\alpha$, 0.71073 | $MoK_\alpha$, 0.71073 | $CuK_\alpha$, 1.54184 | $CuK_\alpha$, 1.54184 |
| Crystal system | monoclinic | monoclinic | monoclinic | triclinic |
| Space group | $P2_1$/n | $P2_1$/c | $P2_1$ | $P\bar{1}$ |
| $a$/Å | 10.040(3) | 11.6705(5) | 9.81472(6) | 10.0646(3) |
| $b$/Å | 14.612(6) | 11.4351(5) | 30.3107(2) | 11.5240(4) |
| $c$/Å | 14.451(4) | 19.4944(8) | 16.86116(12) | 15.6916(5) |
| $\alpha$/° | 90 | 90 | 90 | 105.086(3) |
| $\beta$/° | 90.177(9) | 99.7240(10) | 90.0056(6) | 98.763(3) |
| $\gamma$/° | 90 | 90 | 90 | 108.320(3) |
| Volume/Å$^3$ | 2120.0(12) | 2564.21(19) | 5016.04(6) | 1612.85(9) |
| $Z$ | 4 | 4 | 8 | 4 |
| 2Θ/° | 3.96–53.46 | 3.54–52.08 | 5.24–150.63 | 6.03–153.86 |
| Reflections collected | 40200 | 32404 | 255080 | 32353 |
| Unique reflections | 4510 | 5057 | 20250 | 12402 |
| Parameters / restraints | 289/0 | 383/0 | 1154/1 | 463/122 |
| GooF on $F^2$ | 1.076 | 1.032 | 1.039 | 1.046 |
| $R_1$ [I>=2σ (I)] | 0.0443 | 0.0432 | 0.0575 | 0.0654 |
| $wR_2$ [all data] | 0.1072 | 0.1134 | 0.1629 | 0.1955 |
| Max./min. res. electron density/ e Å$^{-3}$ | 0.33 / –0.26 | 0.30 / –0.26 | 0.39 / –0.28 | 0.30 / –0.35 |

Table S1. Continued.

| | **5** | **9-($Me_2S$)-9-Br-9-borafluorene** | **1-MeS-2-($Me_2S$-9-borafluorene)-1,2-$C_2B_{10}H_{10}$** | **9-(4-bromobutoxy)-9-borafluorene** |
|---|---|---|---|---|
| CCDC | 2174249 | 2216647 | 2216648 | 2216649 |
| Empirical formula | $C_{38}H_{50}B_{12}Br_2LiO_3$, $C_{16}H_{32}LiO_4$ | $C_{14}H_{14}BBrS$ | $C_{17}H_{27}B_{11}S_2$ | $C_{16}H_{16}BBrO$ |
| $\rho_{calc}$/g·cm$^{-3}$ | 1.132 | 1.525 | 1.256 | 1.513 |
| $F$(000) | 2392 | 616 | 864 | 640 |
| Crystal size/mm$^3$ | 0.24×0.13×0.12 | 0.28×0.20×0.16 | 0.34×0.04×0.02 | 0.21×0.03×0.03 |
| Crystal colour, habit | yellow block | colourless block | colourless needle | colourless needle |
| $\mu$/mm$^{-1}$ | 2.082 | 5.445 | 2.180 | 3.933 |
| $M_r$/g·mol$^{-1}$ | 1146.60 | 305.03 | 414.41 | 315.01 |
| Temperature/K | 173.01(10) | 100(2) | 100(2) | 100(2) |
| Radiation, λ/Å | CuK$_\alpha$, 1.54184 | CuK$_\alpha$, 1.54184 | CuK$_\alpha$, 1.54184 | CuK$_\alpha$, 1.54184 |
| Crystal system | orthorhombic | monoclinic | monoclinic | monoclinic |
| Space group | $P2_12_12_1$ | $P2_1$/n | $P2_1$/c | $P2_1/c$ |
| $a$/Å | 10.14540(10) | 11.8821(4) | 13.0013(3) | 16.3598(3) |
| $b$/Å | 15.55010(10) | 6.7210(2) | 19.0378(4) | 5.11900(10) |
| $c$/Å | 37.9243(2) | 17.0968(5) | 8.8640(2) | 16.5080(3) |
| $\alpha$/° | 90 | 90 | 90 | 90 |
| $\beta$/° | 90 | 103.399(3) | 93.062(2) | 90.050(2) |
| $\gamma$/° | 90 | 90 | 90 | 90 |
| Volume/Å$^3$ | 5983.02(8) | 1328.18(7) | 2190.85(9) | 1382.48(4) |
| $Z$ | 4 | 4 | 4 | 4 |
| 2Θ/° | 4.66–151.03 | 4.12–75.19 | 3.40–74.50 | 2.70–74.94 |
| Reflections collected | 131709 | 11292 | 35742 | 8247 |
| Unique reflections | 12305 | 2694 | 8080 | 2619 |
| Parameters / restraints | 919 / 624 | 156 / 0 | 275 / 0 | 172 / 0 |
| GooF on $F^2$ | 1.020 | 1.083 | 1.089 | 1.084 |
| $R_1$ [I>=2σ (I)] | 0.0519 | 0.0268 | 0.0574 | 0.0633 |
| $wR_2$ [all data] | 0.1512 | 0.0718 | 0.1634 | 0.0643 |
| Max./min. res. electron density/ e Å$^{-3}$ | 0.65 / –0.60 | 0.75 / –0.66 | 0.93 / –0.60 | 0.36 / –0.28 |

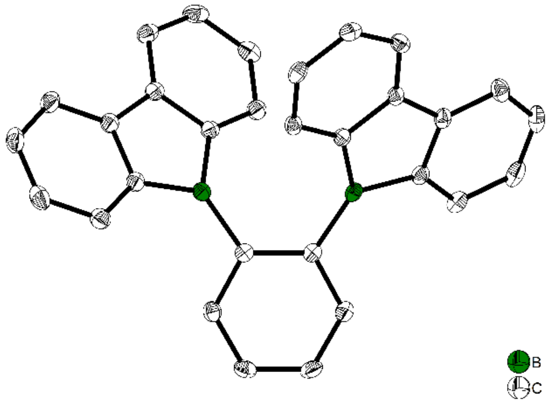


Figure S1. Solid state molecular structure of **2b** from single-crystal X-ray diffraction at 100 K. Atomic displacement ellipsoids are drawn at the 50% probability level and hydrogen atoms are omitted for clarity.

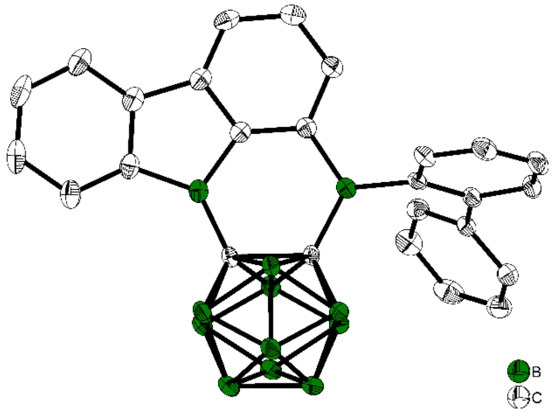


Figure S2. Solid state molecular structure of **3a** from single-crystal X-ray diffraction at 100 K. Atomic displacement ellipsoids are drawn at the 50% probability level and hydrogen atoms are omitted for clarity.

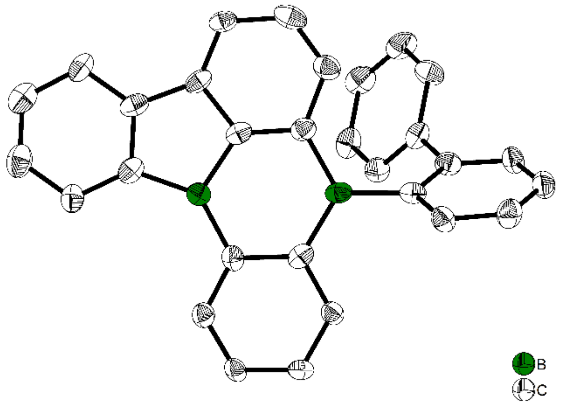


Figure S3. Solid state molecular structure of **3b** from single-crystal X-ray diffraction at 100 K. Atomic displacement ellipsoids are drawn at the 50% probability level and hydrogen atoms and solvent molecules are omitted for clarity. Only one of four symmetry-independent molecules is shown.

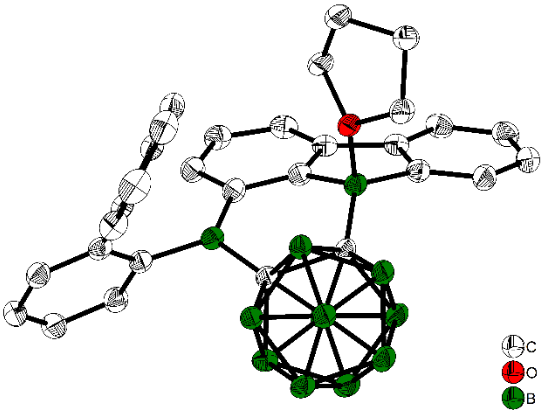


Figure S4. Solid state molecular structure of **3a·THF** from single-crystal X-ray diffraction at 100 K. Atomic displacement ellipsoids are drawn at the 50% probability level and solvent molecules and hydrogen atoms are omitted for clarity.

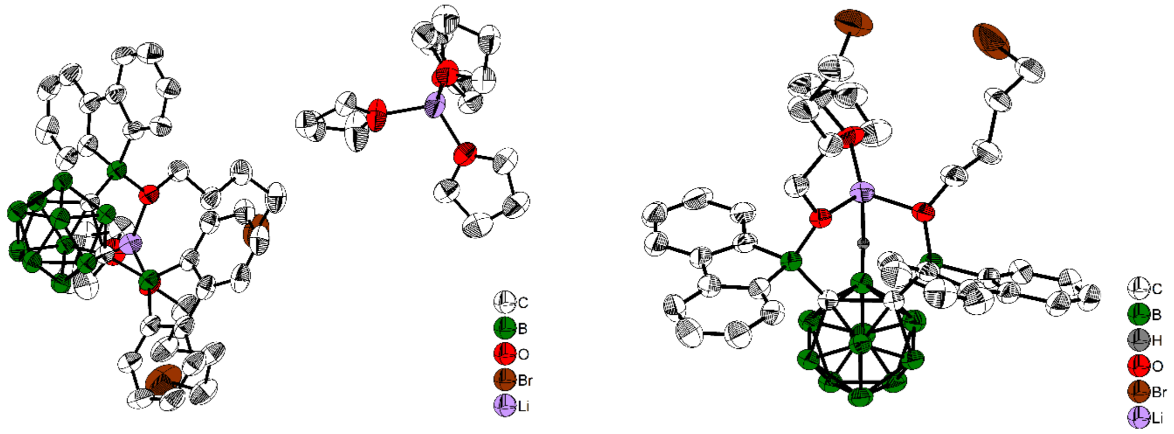


Figure S5. Solid state molecular structure of **5** from single-crystal X-ray diffraction at 100 K, on the left with the lithium counterion and on the right from a different angle. Atomic displacement ellipsoids are drawn at the 50% probability level, and hydrogen atoms and the minor occupied components of disordered THF and alkyl groups are omitted for clarity. Only one of two symmetry-independent anions and cations are shown.

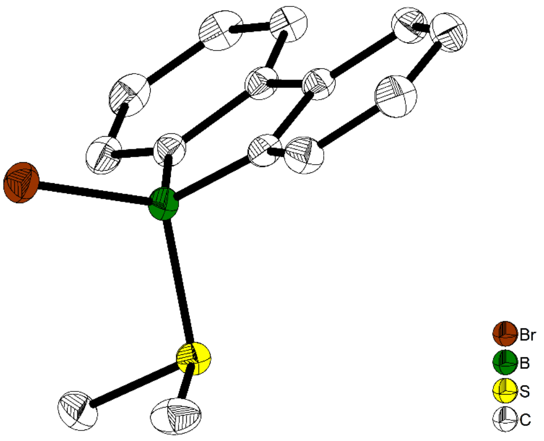


Figure S6. Solid state molecular structure of the dimethyl sulfide adduct of 9-Br-9-borafluorene, **9-($Me_2S$)-9-Br-9-borafluorene**, from single-crystal X-ray diffraction at 100 K. Atomic displacement ellipsoids are drawn at the 50% probability level and hydrogen atoms are omitted for clarity.

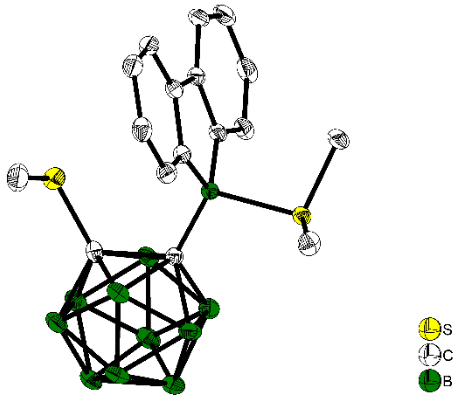


Figure S7. Solid state molecular structure of an isolated product **1-MeS-2-(Me$_2$S-9-borafluorene)-1,2-C$_2$B$_{10}$H$_{10}$** from the reaction in SMe$_2$ described above from single-crystal X-ray diffraction at 100 K. Atomic displacement ellipsoids are drawn at the 50% probability level and hydrogen atoms are omitted for clarity.

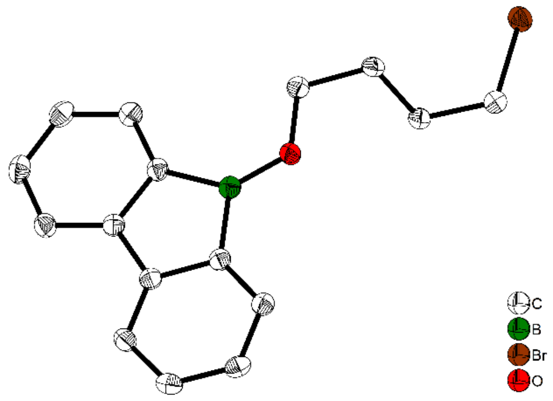


Figure S8. Solid state molecular structure of **9-(4-bromobutoxy)-9-borafluorene** from single-crystal X-ray diffraction at 100 K. Atomic displacement ellipsoids are drawn at the 50% probability level and hydrogen atoms are omitted for clarity.

# Geometry tables

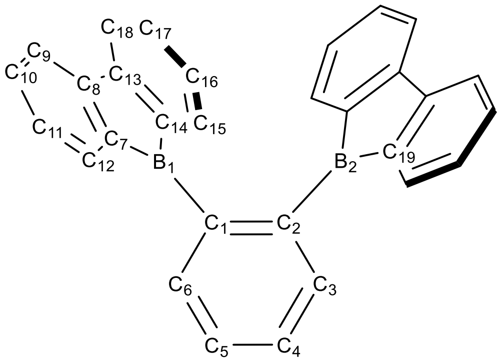


Scheme S1. Atom labeling in compound **2b**.

Table S2. Selected bond lengths [Å], distances [Å], and angles [°] in the crystal and calculated at the B3LYP-d3bj and ωB97X-D level of theory (starting molecule).

| | **2a** calc. B3LYP-d3bj | **2a** calc. ωB97X-D | **2b** crystal | **2b** calc. B3LYP-d3bj | **2b** calc. ωB97X-D |
|---|---|---|---|---|---|
| $C_1$–$C_2$ | 1.674 | 1.674 | 1.426(2) | 1.426 | 1.420 |
| $C_1$–$C_6$ / $C_2$–$C_3$ | – | – | 1.402(2) / 1.405(2) | 1.404 | 1.402 |
| $C_5$–$C_6$ / $C_3$–$C_4$ | – | – | 1.388(2) / 1.389(2) | 1.396 | 1.393 |
| $C_4$–$C_5$ | – | – | 1.386(2) | 1.395 | 1.393 |
| $B_1$–$C_1$[a] | 1.591 | 1.600 | 1.554(2) / 1.551(2) | 1.550 | 1.556 |
| $B_1$–$C_{14}$[a] | 1.566 | 1.570 | 1.571(2) / 1.571(2) | 1.564 | 1.568 |
| $B_1$–$C_7$[a] | 1.562 | 1.566 | 1.568(2) / 1.571(2) | 1.565 | 1.570 |
| $B_1$–$B_2$ | 3.025 | 3.054 | 3.186(2) | 3.005 | 3.083 |
| $B_2$–$C_{15}$[a] | 3.428 | 3.487 | 3.252(2) / 3.354(2) | 3.153 | 3.231 |
| $C_{14}$–$C_{13}$[a] | 1.426 | 1.418 | 1.417(2) / 1.415(2) | 1.421 | 1.414 |
| $C_7$–$C_8$[a] | 1.421 | 1.413 | 1.416(2) / 1.419(2) | 1.422 | 1.413 |
| $C_8$–$C_{13}$ [a] | 1.480 | 1.483 | 1.487(2) / 1.489(2) | 1.485 | 1.489 |
| $C_1$–$B_1$–$C_{14}$ [a] | 129.0 | 128.8 | 128.1(1) / 128.5(1) | 126.7 | 127.9 |
| $C_{14}$–$B_1$–$C_7$ [a] | 105.1 | 105.0 | 103.9(1) / 103.7(1) | 104.2 | 103.9 |
| $C_7$–$B_1$–$C_1$ [a] | 125.1 | 125.1 | 127.5(1) / 127.5(1) | 128.9 | 128.2 |
| ΣC–$B_1$–C[a] | 359.2 | 358.9 | 359.5(3) / 359.8(3) | 359.8 | 359.8 |
| $C_2$–$C_1$–$B_1$, $C_1$–$C_2$–$B_2$ | 115.0 | 115.6 | 124.9(1) / 123.6(1) | 120.4 | 122.1 |
| $B_1$–$C_1$–$C_2$–$B_2$ | 5.8 | 6.3 | 13.2(2) | 8.5 | 9.4 |

| | | | | | |
|---|---|---|---|---|---|
| $C_2$–$C_1$–$B_1$–$C_{14}$[a] | 58.8 | 59.5 | 43.6(2) / 39.2(2) | 43.3 | 43.5 |
| $C_{15}$–$C_{14}$–$B_1$–$C_1$[a] | 11.2 | 12.8 | 4.7(2) / 2.7(2) | 8.1 | 2.6 |

[a] Values are given for both borafluorene moieties of B1 and B2, respectively, for the crystal of **2b**.

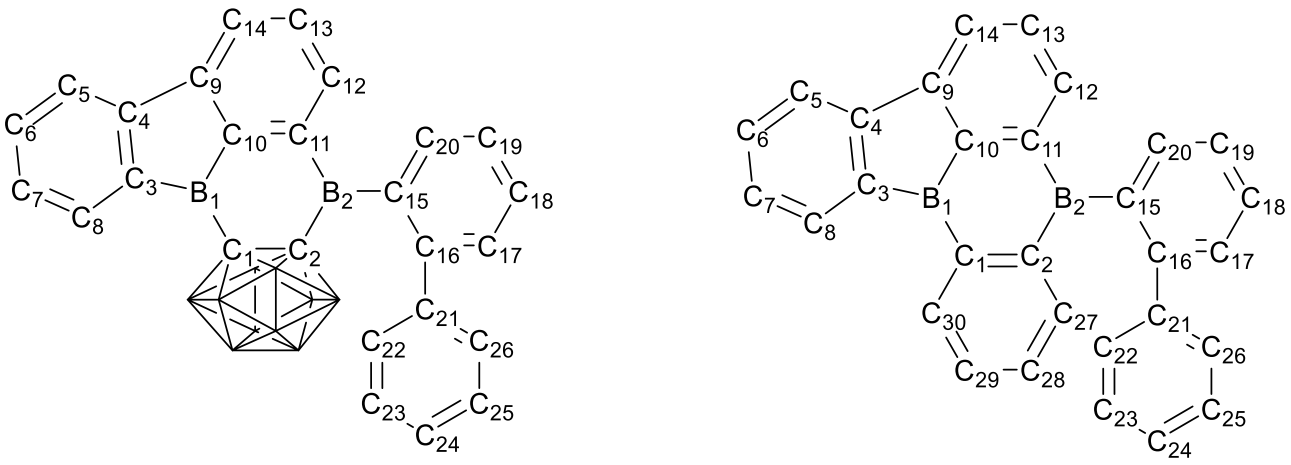

Scheme S2. Atom labeling in compounds **3a** and **3b**.

Table S3. Selected bond lengths [Å], distances [Å], and angles [°] in the crystals and calculated structures of **3a** and **3b** at the B3LYP-d3bj and ωB97X-D level of theory.

| | **3a** crystal | **3a** B3LYP-d3bj | **3a** ωB97X-D | **3b** crystal[a] | **3b** B3LYP-d3bj | **3b** ωB97X-D | **3a·THF** crystal |
|---|---|---|---|---|---|---|---|
| $C_1$–$C_2$ | 1.718(2) | 1.712 | 1.693 | 1.460(8), 1.409(7), 1.425(7), 1.425(7) | 1.435 | 1.429 | 1.717(2) |
| $C_2$–$C_{27}$ | – | – | – | 1.399(7), 1.401(7), 1.423(7), 1.402(7) | 1.402 | 1.399 | – |
| $C_{27}$–$C_{28}$ | – | – | – | 1.393(7), 1.389(8), 1.380(8), 1.396(7) | 1.397 | 1.394 | – |
| $C_{28}$–$C_{29}$ | – | – | – | 1.403(7), 1.360(9), 1.403(9), 1.390(8) | 1.394 | 1.390 | – |
| $C_{29}$–$C_{30}$ | – | – | – | 1.364(8), 1.402(9), 1.417(8), 1.376(8) | 1.396 | 1.393 | – |
| $C_{30}$–$C_1$ | – | – | – | 1.379(7), 1.407(8), 1.384(8), 1.391(8) | 1.403 | 1.398 | – |
| $B_1$–$C_1$ | 1.551(2) | 1.549 | 1.555 | 1.555(8), 1.548(8), 1.565(8), 1.554(8) | 1.541 | 1.547 | 1.624(3) |
| $B_1$–$C_3$ | 1.546(2) | 1.549 | 1.551 | 1.545(9), 1.591(9), | 1.570 | 1.574 | 1.622(2) |

| | | | | | | | |
|---|---|---|---|---|---|---|---|
| | | | | 1.553(9), 1.604(9) | | | |
| $B_1$–$C_{10}$ | 1.538(2) | 1.541 | 1.544 | 1.567(9), 1.554(9), 1.561(9), 1.533(9) | 1.549 | 1.553 | 1.595(3) |
| $B_2$–$C_2$ | 1.607(2) | 1.599 | 1.604 | 1.587(9), 1.597(8), 1.570(8), 1.573(8) | 1.583 | 1.588 | 1.603(3) |
| $B_2$–$C_{11}$ | 1.557(2) | 1.557 | 1.562 | 1.558(8), 1.577(8), 1.557(8), 1.564(8) | 1.563 | 1.567 | 1.541(2) |
| $B_2$–$C_{15}$ | 1.572(2) | 1.562 | 1.568 | 1.593(8), 1.577(8), 1.556(9), 1.604(8) | 1.567 | 1.574 | 1.572(2) |
| $C_3$–$C_4$ | 1.424(2) | 1.430 | 1.422 | 1.401(9), 1.431(9), 1.457(9), 1.423(8) | 1.432 | 1.424 | 1.419(3) |
| $C_9$–$C_{10}$ | 1.406(2) | 1.410 | 1.403 | 1.415(9), 1.421(9), 1.414(8), 1.396(8) | 1.405 | 1.399 | 1.399(2) |
| $C_4$–$C_9$ | 1.492(2) | 1.492 | 1.495 | 1.470(10), 1.450(10), 1.440(10), 1.545(8) | 1.491 | 1.493 | 1.485(2) |
| $B_1$–$C_1$–$C_2$ | 114.4(1) | 114.2 | 114.3 | 116.6(5), 117.9(5), 117.5(4), 116.9(5) | 117.3 | 117.2 | 114.0(1) |
| $B_2$–$C_2$–$C_1$ | 118.5(1) | 118.5 | 118.8 | 122.3(5), 122.9(4), 124.0(5), 122.3(5) | 122.7 | 122.7 | 119.6(1) |
| $C_1$–$B_1$–$C_{10}$ / $C_{10}$–$B_1$–$C_3$ / $C_3$–$B_1$–$C_1$ | 119.5(1) / 105.5(1) / 135.0(1) | 120.0 / 105.2 / 134.8 | 119.9 / 105.1 / 134.9 | 118.4(5) / 104.0(5) / 137.6(5), 117.5(5) / 103.8(5) / 138.7(5), 116.9(5) / 102.4(5) / 140.6(5), 118.8(5) / 103.7(5) / 137.5(5) | 118.5 / 103.5 / 138.0 | 118.5 / 103.3 / 138.2 | 112.1(1) / 100.0(1) / 123.9(1) |
| ΣC–$B_1$–C | 360.0 | 360.0 | 359.9 | 360.0 | 360.0 | 360.0 | 336.0 |
| $C_2$–$B_2$–$C_{15}$ / $C_{15}$–$B_2$–$C_{11}$ / $C_{15}$–$B_2$–$C_2$ | 119.6(1) / 120.0(1) / 119.2(1) | 120.1 / 119.6 / 119.7 | 119.9 / 119.9 / 119.6 | 120.6(5) / 119.3(5) / 120.1(5), 117.8(5) / 120.9(5) / 121.3(5), 119.1(5) / 119.2(5) / 121.7(5), | 120.9 / 119.9 / 119.2 | 120.9 / 119.8 / 119.2 | 118.3(1) / 122.2(2) / 118.1(1) |

| | | | | | | | |
|---|---|---|---|---|---|---|---|
| | | | | 120.2(5) / 119.5(5) / 120.3(5) | | | |
| ΣC–$B_2$–C | 358.8 | 359.4 | 359.4 | 360.0 | 360.0 | 359.9 | 358.6 |
| $B_1$–$C_1$–$C_2$–$B_2$ | 2.6(2) | 5.5 | 3.6 | 3.3(8), 3.3(7), 1.9(7), 3.4(7) | 2.2 | 2.7 | 0.9(2) |
| $C_2$–$B_2$–$C_{15}$–$C_{16}$ | 82.4(1) | 76.2 | 79.4 | 58.2(6), 60.2(6), 60.7(6), 58.2(6) | 61.1 | 63.3 | 78.6(2) |
| $C_2$–$C_1$–$B_1$–$C_3$ | 176.1(1) | 176.6 | 177.9 | 175.4(6), 176.7(6), 176.0(7), 176.1(6) | 179.4 | 179.5 | 142.5(2) |
| $C_1$–$C_2$–$B_2$–$C_{15}$ | 159.7(2) | 166.3 | 170.4 | 178.0(5), 178.1(5), 177.1(5), 179.4(5) | 175.3 | 176.1 | 176.0(1) |
| $C_{15}$–$C_{16}$–$C_{21}$–$C_{26}$ | 140.8(2) | 141.6 | 136.6 | 126.0(6), 128.6(6), 128.9(6), 126.0(6) | 136.7 | 133.4 | 133.3(2) |

[a] Four symmetry-independent molecules are present in the crystal structure of **3b**. Values are given for all four molecules.

Table S4. Selected bond lengths [Å], distances (B–B, B–$C_{15}$ [Å]), and angles [°] of the calculated transition state structures at the ωB97X-D level of theory.

| | **2a-3a** | **2b-3b** |
|---|---|---|
| $C_1$–$C_1$* | 1.689 | 1.422 |
| $B_1$–$B_1$ | 3.229 | 3.101 |
| $B_1$–$C_1$ | 1.551 | 1.534 |
| $B_1$–$C_3$ | 1.551 | 1.577 |
| $B_1$–$C_{10}$ | 1.548 | 1.554 |
| $B_1$–$C_2$ | 1.638 | 1.610 |
| $B_1$–$C_{15}$ | 1.596 | 1.597 |
| $B_1$–$C_{22}$ | 1.723 | 1.733 |
| $B_1$–$C_{11}$ | 1.771 | 1.750 |
| $B_1$–$C_1$–$C_2$ | 114.6 | 118.1 |
| $B_2$–$C_2$–$C_1$ | 123.0 | 126.4 |
| $B_1$–$C_1$–$C_2$–$B_2$ | 2.6 | 4.7 |
| $C_2$–$C_1$–$B_1$–$C_3$ | 175.0 | 172.6 |
| $C_{15}$–$C_{16}$–$C_{21}$–$C_{22}$ | 176.2 | 176.6 |

# Photophysical data

## 2b

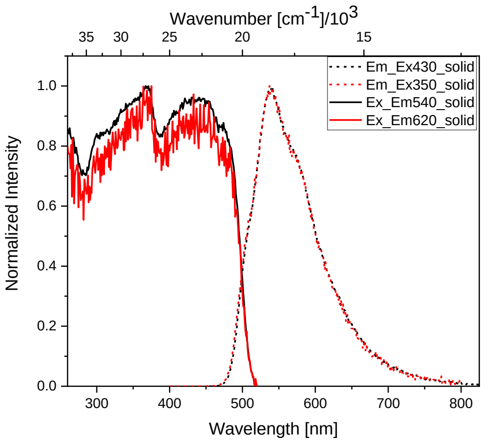


Figure S9. Excitation (solid) and emission (dashed) spectra of **2b** in the solid state.

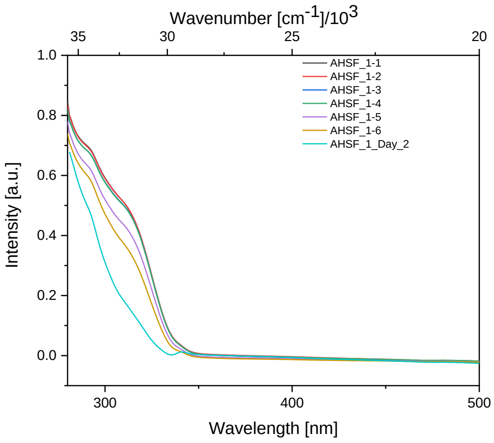


Figure S10. Absorption spectra of compound **2b** in toluene.

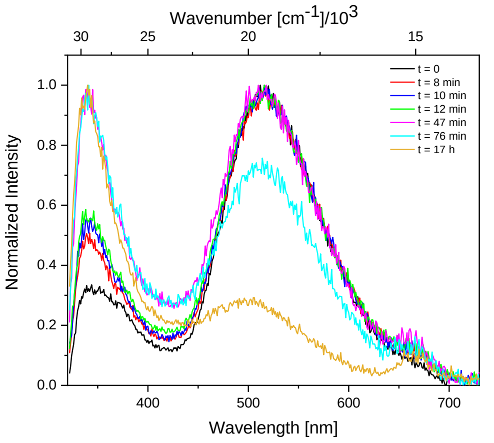


Figure S11. Normalized emission spectra of compound **2b** in toluene.

**3a**

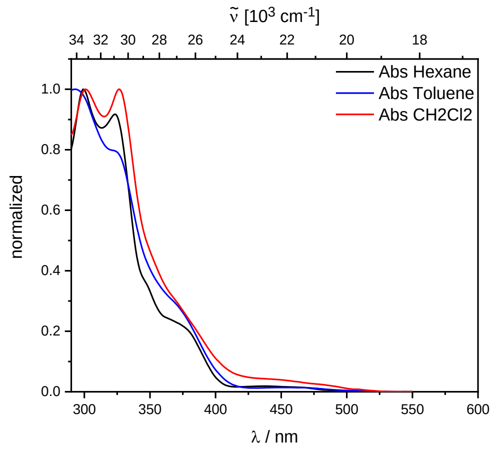


Figure S12. Absorption spectra of **3a** in hexane (black), toluene (blue) and $CH_2Cl_2$ (red).

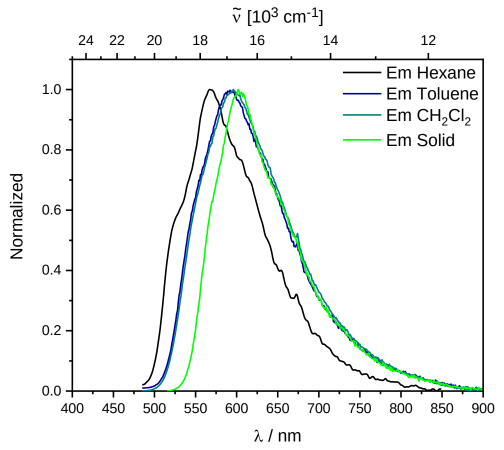


Figure S13. Emission spectra of **3a** in hexane (black), toluene (blue), $CH_2Cl_2$ (turquoise) and in the solid state (green).

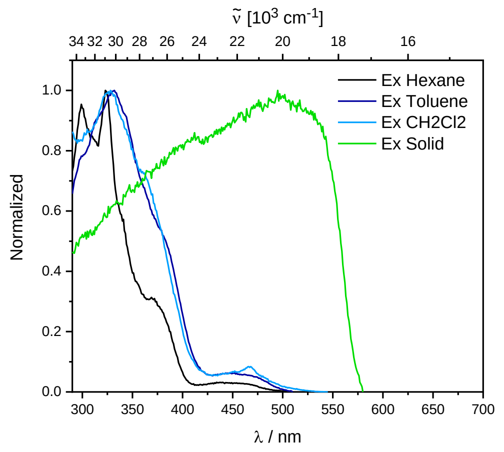


Figure S14. Excitation spectra of **3a** in hexane (black), toluene (blue), $CH_2Cl_2$ (turquoise) and in the solid state (green).

## 3b

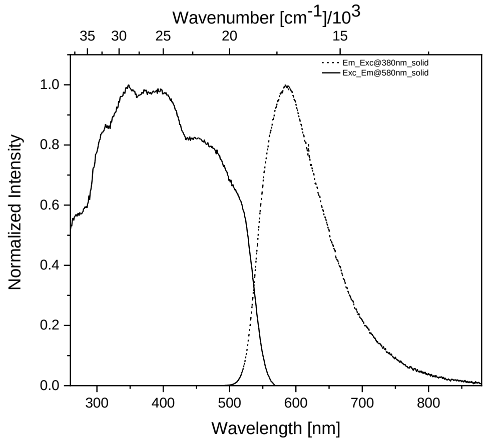


Figure S15. Excitation (solid) and emission (dashed) spectra of **3b** in the solid state.

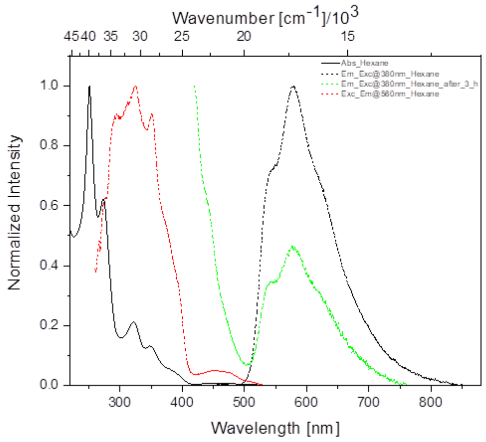


Figure S16. Absorption (black, solid), emission (black, dashed) and excitation (red, dashed) spectra of **3b** in hexane initially and after 3 h (green, dashed).

# Cyclic voltammetry

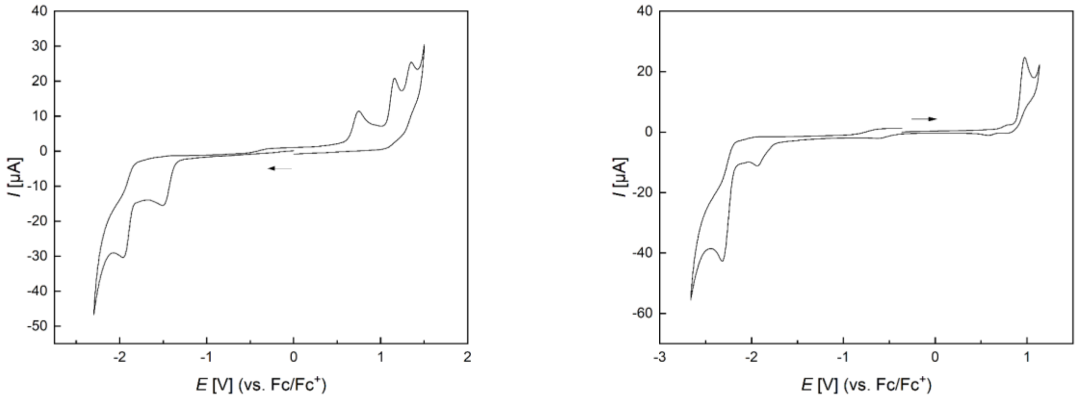


Figure S17. Cyclic voltammograms of **2b** measured in $CH_2Cl_2$ with $[nBu_4N][PF_6]$ as the electrolyte with a scan rate of 250 $mVs^{-1}$. All measurements are referenced to the $Fc/Fc^+$ ion couple.

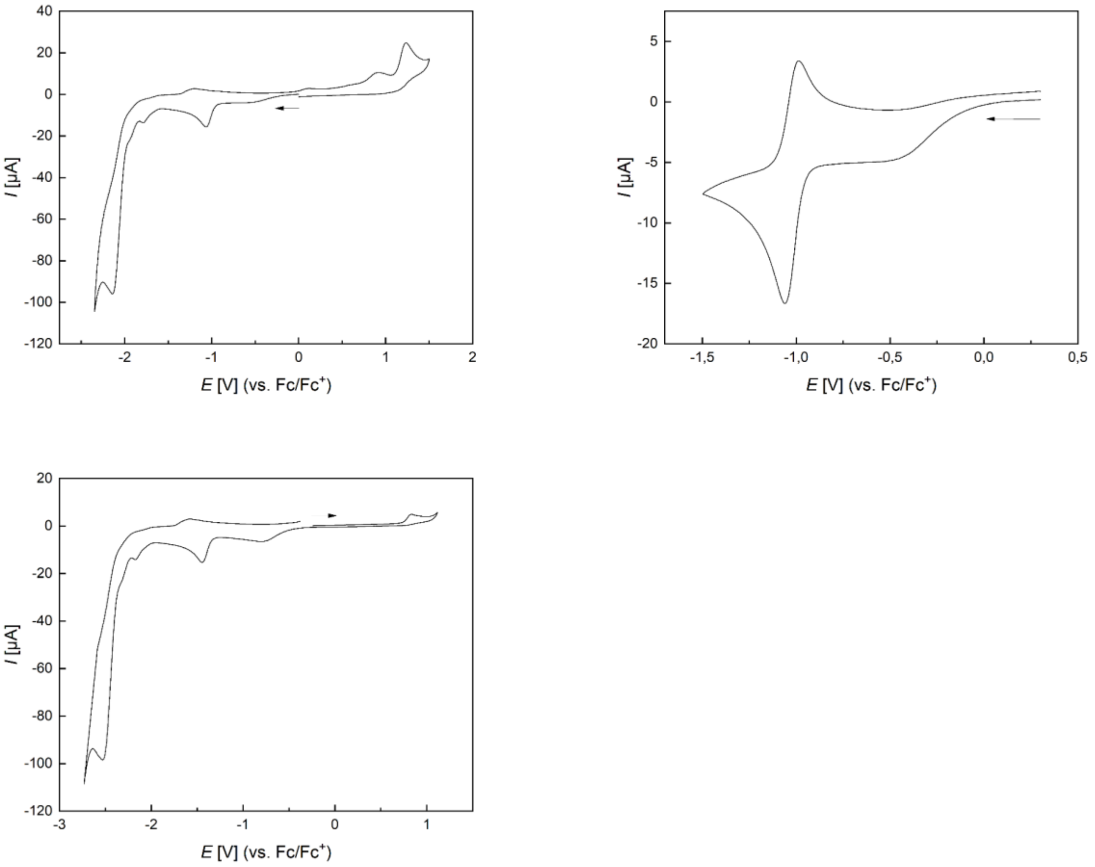


Figure S18. Cyclic voltammograms of **3a** measured in $CH_2Cl_2$ with $[nBu_4N][PF_6]$ as the electrolyte with a scan rate of 250 $mVs^{-1}$. All measurements are referenced to the $Fc/Fc^+$ ion couple.

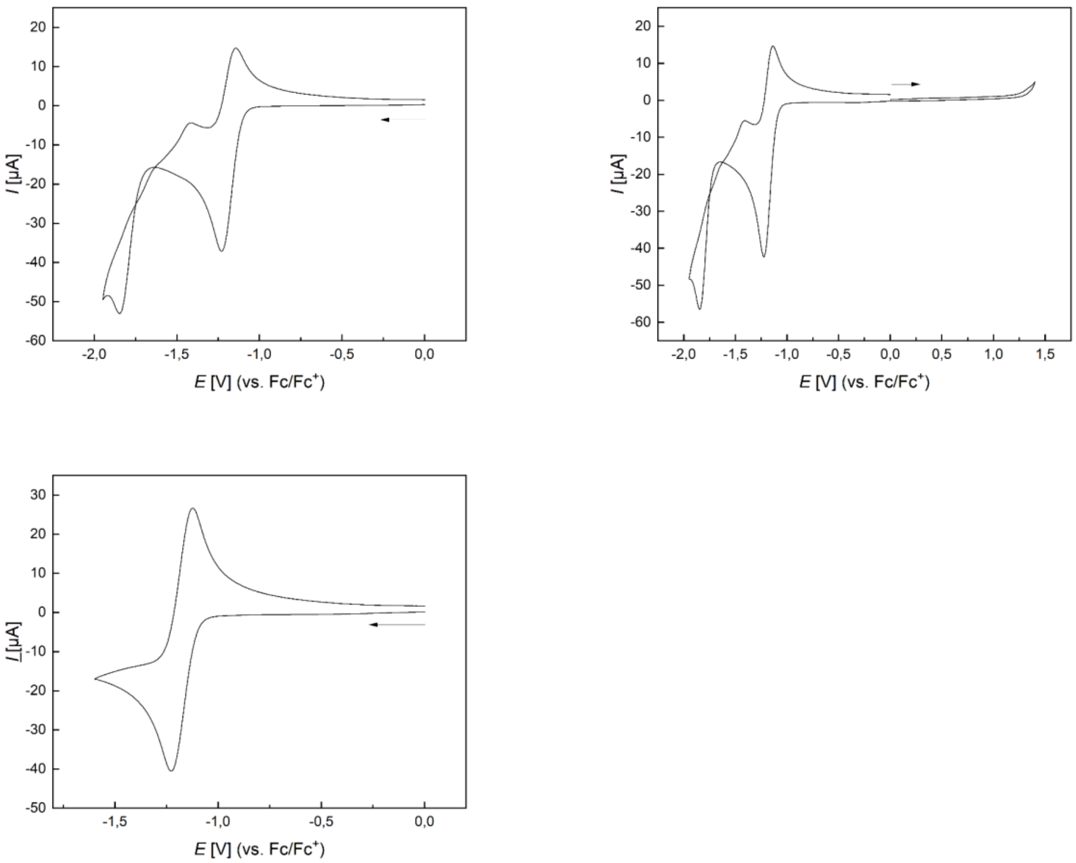


Figure S19. Cyclic voltammograms of **3b** measured in $CH_2Cl_2$ with $[nBu_4N][PF_6]$ as the electrolyte with a scan rate of 250 $mVs^{-1}$. All measurements are referenced to the $Fc/Fc^+$ ion couple.

Table S5. Reduction and oxidation potentials of **2b**, **3a** and **3b**.

| **compound** | **1st reduction** | **2nd reduction** | **1st oxidation** |
|---|---|---|---|
| **2a** | $E_{pc}$ = –1.50 V | $E_{pc}$ = –1.96 V | $E_{pa}$ = 0.98 V |
| **3a** | $E_{1/2}$[a] = –1.03 V | $E_{pc}$ = –2.14 V | $E_{pa}$ = 0.84 V |
| **3b** | $E_{1/2}$ = –1.17 V | $E_{pc}$ = –1.85 V | |

[a] partially reversible.

# DFT and TD-DFT results

## Cartesian coordinates of the optimized structures of 2a, 2b, 3a and 3b in the transition state calculations

64

M062X_**2a** (E = –1304.381314 a.u.)

| | | | |
|---|---|---|---|
| H | -1.674587000 | -1.681467000 | 1.646903000 |
| B | -2.396994000 | 1.018008000 | -1.001561000 |
| C | 1.869236000 | -1.188931000 | -1.413385000 |
| B | -0.488890000 | 1.138247000 | 1.004461000 |
| H | -1.674526000 | 1.681308000 | -1.646761000 |
| H | -3.097740000 | -0.663864000 | -2.776306000 |
| B | -0.488843000 | -1.138328000 | -1.004368000 |
| C | -0.470187000 | 3.699129000 | 0.057367000 |
| H | -1.532345000 | 3.760452000 | -0.152272000 |
| C | -1.930180000 | -0.588996000 | -0.581832000 |
| B | -3.273957000 | -1.630229000 | -0.394163000 |
| H | -4.727725000 | 1.750487000 | -1.743227000 |
| C | 0.076294000 | -2.554611000 | -0.634751000 |
| B | -3.274014000 | 1.630023000 | 0.394213000 |
| H | -5.689258000 | 1.081175000 | 1.091802000 |
| H | -5.689201000 | -1.081406000 | -1.091865000 |
| C | -1.930200000 | 0.588828000 | 0.581919000 |
| B | -2.397014000 | -1.018184000 | 1.001649000 |
| C | 0.353989000 | -4.780703000 | 0.268711000 |
| H | -0.075436000 | -5.673148000 | 0.711697000 |
| H | -4.727743000 | -1.750707000 | 1.743230000 |
| B | -4.151941000 | 1.018463000 | -1.012159000 |
| H | -3.097837000 | 0.663680000 | 2.776354000 |
| B | -4.699826000 | -0.623079000 | -0.630222000 |
| C | 1.723278000 | -4.714991000 | 0.031173000 |
| H | 2.355234000 | -5.558486000 | 0.292214000 |
| B | -3.255077000 | -0.380962000 | -1.638639000 |
| C | 2.295324000 | -3.570392000 | -0.532886000 |
| H | 3.367848000 | -3.520197000 | -0.696435000 |
| B | -4.151956000 | -1.018677000 | 1.012172000 |
| H | -3.143274000 | 2.760189000 | 0.711041000 |
| C | 1.470733000 | -2.507193000 | -0.861689000 |
| B | -3.255139000 | 0.380768000 | 1.638686000 |
| H | -3.143175000 | -2.760385000 | -0.710982000 |
| C | 0.738957000 | -0.349423000 | -1.592295000 |
| B | -4.699859000 | 0.622850000 | 0.630207000 |
| C | 3.150651000 | -0.752242000 | -1.696508000 |
| H | 4.009764000 | -1.396208000 | -1.532270000 |
| C | 3.329029000 | 0.548045000 | -2.183464000 |
| H | 4.330528000 | 0.907349000 | -2.400518000 |
| C | 2.236141000 | 1.377337000 | -2.397943000 |
| H | 2.381679000 | 2.382910000 | -2.778738000 |
| C | 0.942537000 | 0.929599000 | -2.108852000 |
| H | 0.114487000 | 1.605678000 | -2.280019000 |
| C | 0.076197000 | 2.554520000 | 0.634723000 |
| C | 0.353610000 | 4.780656000 | -0.268714000 |
| H | -0.075928000 | 5.673035000 | -0.711724000 |
| C | 1.722891000 | 4.715156000 | -0.031075000 |
| H | 2.354737000 | 5.558743000 | -0.292087000 |
| C | 2.295083000 | 3.570647000 | 0.533017000 |
| H | 3.367604000 | 3.520624000 | 0.696623000 |
| C | 1.470634000 | 2.507321000 | 0.861769000 |
| C | 1.869285000 | 1.189098000 | 1.413428000 |
| C | 3.150758000 | 0.752438000 | 1.696353000 |
| H | 4.009827000 | 1.396457000 | 1.532069000 |
| C | 3.329236000 | -0.547861000 | 2.183246000 |
| H | 4.330770000 | -0.907135000 | 2.400187000 |
| C | 2.236402000 | -1.377217000 | 2.397737000 |
| H | 2.381991000 | -2.382801000 | 2.778483000 |
| C | 0.942748000 | -0.929528000 | 2.108772000 |
| H | 0.114756000 | -1.605647000 | 2.280092000 |
| C | 0.739044000 | 0.349571000 | 1.592463000 |

64

M062X_TS1-**2a/3a** (E = -1304.336144 a.u.)

| | | | |
|---|---|---|---|
| H | -0.407581000 | 1.437831000 | 2.240836000 |
| B | -0.519596000 | 1.543845000 | -1.575714000 |
| C | -3.686268000 | -1.628311000 | 0.248783000 |
| C | 0.004446000 | -1.521727000 | 0.288362000 |
| B | 1.125593000 | -0.174233000 | -0.007547000 |
| H | -0.662877000 | 0.641241000 | -2.324598000 |
| H | -2.894468000 | 2.458684000 | -1.659471000 |
| B | -2.083181000 | 0.138270000 | 0.178967000 |
| C | 1.825746000 | -0.998305000 | -2.490076000 |
| H | 0.815854000 | -0.903676000 | -2.877115000 |
| C | -1.294936000 | 1.451894000 | -0.070048000 |
| B | -1.744010000 | 2.941521000 | 0.592188000 |
| H | -0.398761000 | 3.665765000 | -3.022050000 |
| C | -1.349904000 | -1.221432000 | 0.310396000 |
| B | 1.004912000 | 2.382210000 | -1.261320000 |
| H | 1.342505000 | 4.904448000 | -0.821578000 |
| H | -1.700638000 | 5.183233000 | -0.691664000 |
| C | 0.369241000 | 1.272795000 | -0.135796000 |
| B | -0.353586000 | 2.026678000 | 1.217381000 |
| C | 0.393805000 | -2.874638000 | 0.274566000 |
| H | 1.449955000 | -3.132851000 | 0.267471000 |
| H | -0.115609000 | 4.509082000 | 1.845563000 |
| B | -0.386647000 | 3.276088000 | -1.904186000 |
| H | 2.131714000 | 2.569271000 | 1.045538000 |
| B | -1.138165000 | 4.150789000 | -0.548007000 |
| C | -0.570873000 | -3.874530000 | 0.268312000 |
| H | -0.257261000 | -4.913626000 | 0.254717000 |
| B | -1.846269000 | 2.643815000 | -1.141426000 |
| C | -1.941413000 | -3.570071000 | 0.270891000 |
| H | -2.674379000 | -4.371511000 | 0.251418000 |
| B | -0.220940000 | 3.766967000 | 0.928819000 |
| H | 1.959389000 | 2.052741000 | -1.874271000 |
| C | -2.324852000 | -2.239507000 | 0.285473000 |
| B | 1.106242000 | 2.683938000 | 0.470689000 |
| H | -2.721684000 | 2.961231000 | 1.260079000 |
| C | -3.597266000 | -0.212907000 | 0.194468000 |
| B | 0.620439000 | 3.986793000 | -0.622600000 |
| C | -4.914631000 | -2.264422000 | 0.249784000 |
| H | -4.989351000 | -3.347471000 | 0.285902000 |
| C | -6.075425000 | -1.480409000 | 0.202386000 |
| H | -7.046154000 | -1.966783000 | 0.204738000 |
| C | -6.003887000 | -0.092312000 | 0.149497000 |
| H | -6.916415000 | 0.493106000 | 0.111856000 |
| C | -4.759530000 | 0.547258000 | 0.141258000 |
| H | -4.701353000 | 1.631237000 | 0.092939000 |
| C | 2.103014000 | -0.689436000 | -1.159400000 |
| C | 2.848790000 | -1.425382000 | -3.334062000 |
| H | 2.633197000 | -1.648187000 | -4.374304000 |
| C | 4.150561000 | -1.571409000 | -2.851066000 |
| H | 4.938988000 | -1.907153000 | -3.517210000 |
| C | 4.440083000 | -1.298532000 | -1.518490000 |
| H | 5.449780000 | -1.429648000 | -1.139958000 |
| C | 3.414496000 | -0.859143000 | -0.681768000 |
| C | 3.502922000 | -0.545519000 | 0.751904000 |
| C | 4.645550000 | -0.522237000 | 1.548123000 |
| H | 5.616117000 | -0.759498000 | 1.122732000 |
| C | 4.537233000 | -0.180999000 | 2.893869000 |
| H | 5.427229000 | -0.159974000 | 3.514874000 |
| C | 3.298731000 | 0.131363000 | 3.450890000 |
| H | 3.224583000 | 0.388808000 | 4.502040000 |
| C | 2.152156000 | 0.111609000 | 2.658275000 |
| H | 1.189869000 | 0.340253000 | 3.104792000 |

| | | | |
|---|---|---|---|
| H | -1.532117000 | -3.760823000 | 0.152091000 |
| C | -0.469954000 | -3.699310000 | -0.057445000 |

| | | | |
|---|---|---|---|
| C | 2.248987000 | -0.207208000 | 1.303946000 |
| H | 0.982758000 | -0.948858000 | 1.057637000 |

64
M062X_**3a** (E = -1304.39502822 a.u.)

| | | | |
|---|---|---|---|
| H | -0.247664000 | 1.206098000 | 1.836781000 |
| B | -0.474233000 | 1.325253000 | -1.982113000 |
| C | -4.024259000 | -1.229137000 | 0.407632000 |
| C | -0.385690000 | -1.667725000 | -0.118726000 |
| B | 0.743728000 | -0.643482000 | -0.469964000 |
| H | -0.774805000 | 0.448397000 | -2.718164000 |
| H | -2.657528000 | 2.644387000 | -2.006354000 |
| B | -2.214437000 | 0.238233000 | -0.119020000 |
| C | 2.111577000 | -1.850692000 | -2.191904000 |
| H | 1.165526000 | -1.973492000 | -2.715520000 |
| C | -1.220843000 | 1.389605000 | -0.448705000 |
| B | -1.375890000 | 2.934602000 | 0.207296000 |
| H | -0.028559000 | 3.387521000 | -3.451227000 |
| C | -1.689838000 | -1.206490000 | 0.030900000 |
| B | 1.183880000 | 1.894463000 | -1.717123000 |
| H | 1.963567000 | 4.320016000 | -1.312826000 |
| H | -0.972805000 | 5.123273000 | -1.104078000 |
| C | 0.391335000 | 0.922182000 | -0.562573000 |
| B | -0.150843000 | 1.788563000 | 0.812039000 |
| C | -0.175148000 | -3.051901000 | 0.055107000 |
| H | 0.823602000 | -3.466300000 | -0.055052000 |
| H | 0.544447000 | 4.193540000 | 1.397836000 |
| B | -0.050509000 | 3.007077000 | -2.330489000 |
| H | 2.386320000 | 1.900670000 | 0.562422000 |
| B | -0.598563000 | 4.008626000 | -0.961893000 |
| C | -1.240897000 | -3.891256000 | 0.367524000 |
| H | -1.059333000 | -4.953222000 | 0.502206000 |
| B | -1.578863000 | 2.645794000 | -1.518341000 |
| C | -2.550980000 | -3.402167000 | 0.509829000 |
| H | -3.359888000 | -4.087154000 | 0.749009000 |
| B | 0.280328000 | 3.474887000 | 0.494934000 |
| H | 2.042812000 | 1.386228000 | -2.350651000 |
| C | -2.773702000 | -2.045040000 | 0.335717000 |
| B | 1.385509000 | 2.179934000 | 0.007695000 |
| H | -2.315386000 | 3.133376000 | 0.899600000 |
| C | -3.745623000 | 0.140153000 | 0.139290000 |
| B | 1.099575000 | 3.542902000 | -1.083954000 |
| C | -5.311783000 | -1.648905000 | 0.687106000 |
| H | -5.531295000 | -2.692379000 | 0.894094000 |
| C | -6.341464000 | -0.697538000 | 0.700556000 |
| H | -7.356367000 | -1.014970000 | 0.919811000 |
| C | -6.084782000 | 0.643602000 | 0.438646000 |
| H | -6.898176000 | 1.361297000 | 0.454802000 |
| C | -4.780657000 | 1.066707000 | 0.156151000 |
| H | -4.579288000 | 2.114882000 | -0.048220000 |
| C | 2.131691000 | -1.188304000 | -0.953924000 |
| C | 3.276819000 | -2.335463000 | -2.776249000 |
| H | 3.236080000 | -2.826178000 | -3.743215000 |
| C | 4.488557000 | -2.198725000 | -2.104738000 |
| H | 5.402747000 | -2.589333000 | -2.540069000 |
| C | 4.523245000 | -1.581291000 | -0.860931000 |
| H | 5.461069000 | -1.513776000 | -0.317216000 |
| C | 3.360044000 | -1.069438000 | -0.275622000 |
| C | 3.426195000 | -0.426606000 | 1.058499000 |
| C | 4.494953000 | 0.416238000 | 1.386987000 |
| H | 5.282514000 | 0.583526000 | 0.658432000 |
| C | 4.518396000 | 1.090666000 | 2.601152000 |

52
M062X_**2b** (E = -1204.509805 a.u.)

| | | | |
|---|---|---|---|
| C | 2.067705000 | -1.333805000 | 1.333483000 |
| B | -1.480458000 | 0.838310000 | -0.467083000 |
| B | 1.480458000 | 0.838242000 | 0.466672000 |
| C | -3.880835000 | 1.090549000 | 0.780815000 |
| H | -3.668830000 | 2.069722000 | 1.204696000 |
| C | 2.937324000 | 0.455194000 | 0.017885000 |
| C | 5.105068000 | 0.464650000 | -1.046108000 |
| H | 5.848036000 | 0.958299000 | -1.664308000 |
| C | 5.370104000 | -0.795912000 | -0.520886000 |
| H | 6.320912000 | -1.276035000 | -0.732393000 |
| C | 4.425801000 | -1.458971000 | 0.272168000 |
| H | 4.643742000 | -2.447427000 | 0.667077000 |
| C | 3.219352000 | -0.831133000 | 0.535232000 |
| C | 1.021143000 | -0.383950000 | 1.338606000 |
| C | 1.945793000 | -2.537411000 | 2.007885000 |
| H | 2.747397000 | -3.270879000 | 2.001455000 |
| C | 0.760145000 | -2.799215000 | 2.705100000 |
| H | 0.649485000 | -3.740637000 | 3.234901000 |
| C | -0.273768000 | -1.868921000 | 2.733310000 |
| H | -1.182442000 | -2.087666000 | 3.284576000 |
| C | -0.140168000 | -0.652616000 | 2.054155000 |
| H | -0.948360000 | 0.075669000 | 2.091405000 |
| C | -2.937256000 | 0.455296000 | -0.018026000 |
| C | -5.104807000 | 0.464858000 | 1.046339000 |
| H | -5.847701000 | 0.958606000 | 1.664549000 |
| C | -5.369853000 | -0.795843000 | 0.521460000 |
| H | -6.320591000 | -1.275981000 | 0.733249000 |
| C | -4.425652000 | -1.459025000 | -0.271617000 |
| H | -4.643603000 | -2.447588000 | -0.666249000 |
| C | -3.219291000 | -0.831165000 | -0.535034000 |
| C | -2.067743000 | -1.333923000 | -1.333380000 |
| C | -1.945910000 | -2.537593000 | -2.007689000 |
| H | -2.747488000 | -3.271088000 | -2.001038000 |
| C | -0.760389000 | -2.799419000 | -2.705105000 |
| H | -0.649812000 | -3.740874000 | -3.234866000 |
| C | 0.273481000 | -1.869083000 | -2.733626000 |
| H | 1.182053000 | -2.087862000 | -3.285047000 |
| C | 0.139949000 | -0.652701000 | -2.054603000 |
| H | 0.948111000 | 0.075609000 | -2.092033000 |
| C | -1.021232000 | -0.384014000 | -1.338851000 |
| H | 3.669012000 | 2.069364000 | -1.205118000 |
| C | 3.880998000 | 1.090306000 | -0.780964000 |
| C | 1.354787000 | 3.369438000 | 0.298231000 |
| C | 0.692500000 | 2.141997000 | 0.157228000 |
| C | -0.692536000 | 2.142054000 | -0.157605000 |
| C | -1.354773000 | 3.369531000 | -0.298352000 |
| C | -0.678942000 | 4.579300000 | -0.150709000 |
| C | 0.679006000 | 4.579258000 | 0.150818000 |
| H | 2.413921000 | 3.376429000 | 0.545841000 |
| H | -2.413918000 | 3.376612000 | -0.545905000 |
| H | -1.210775000 | 5.518331000 | -0.269463000 |
| H | 1.210848000 | 5.518260000 | 0.269764000 |

| | | | | |
|---|---|---|---|---|
| H | 5.342206000 | 1.759171000 | 2.829741000 | |
| C | 3.475352000 | 0.932181000 | 3.512285000 | |
| H | 3.487539000 | 1.470954000 | 4.453890000 | |
| C | 2.421768000 | 0.075346000 | 3.209774000 | |
| H | 1.615097000 | -0.069181000 | 3.921353000 | |
| C | 2.402963000 | -0.606815000 | 1.996016000 | |
| H | 1.595766000 | -1.306740000 | 1.788480000 | |

52

M062X_TS1-**2b**/**3b** (E = -1204.45650 a.u.)

| | | | |
|---|---|---|---|
| C | -3.785849000 | -1.019905000 | 0.247945000 |
| C | -0.107126000 | -1.195513000 | 0.210714000 |
| B | 1.027562000 | 0.080829000 | -0.201693000 |
| B | -2.031335000 | 0.607994000 | -0.069465000 |
| C | 2.152414000 | -1.113076000 | -2.342356000 |
| H | 1.220726000 | -1.092008000 | -2.902049000 |
| C | -1.436604000 | -0.801293000 | 0.214552000 |
| C | 0.192209000 | -2.564075000 | 0.344989000 |
| H | 1.226874000 | -2.897207000 | 0.356096000 |
| C | -0.842440000 | -3.487014000 | 0.454383000 |
| H | -0.605092000 | -4.542279000 | 0.548745000 |
| C | -2.188828000 | -3.086839000 | 0.442151000 |
| H | -2.974010000 | -3.833487000 | 0.524184000 |
| C | -2.480303000 | -1.736772000 | 0.317342000 |
| C | -3.587564000 | 0.373134000 | 0.044825000 |
| C | -5.056770000 | -1.560505000 | 0.355961000 |
| H | -5.200854000 | -2.626927000 | 0.506189000 |
| C | -6.162475000 | -0.707454000 | 0.273310000 |
| H | -7.163829000 | -1.117867000 | 0.361150000 |
| C | -5.991340000 | 0.659255000 | 0.082520000 |
| H | -6.859119000 | 1.308435000 | 0.024595000 |
| C | -4.704931000 | 1.197611000 | -0.034542000 |
| H | -4.591091000 | 2.268130000 | -0.181781000 |
| C | 2.202306000 | -0.582562000 | -1.055016000 |
| C | 3.296951000 | -1.665134000 | -2.915058000 |
| H | 3.259540000 | -2.065932000 | -3.923398000 |
| C | 4.494978000 | -1.708273000 | -2.198319000 |
| H | 5.379132000 | -2.145679000 | -2.651548000 |
| C | 4.561951000 | -1.196186000 | -0.906456000 |
| H | 5.493968000 | -1.237802000 | -0.349360000 |
| C | 3.416644000 | -0.632730000 | -0.343584000 |
| C | 3.274346000 | -0.024268000 | 0.989546000 |
| C | 4.267130000 | 0.238642000 | 1.931551000 |
| H | 5.296854000 | -0.051838000 | 1.743522000 |
| C | 3.931404000 | 0.896304000 | 3.113177000 |
| H | 4.703419000 | 1.102557000 | 3.848096000 |
| C | 2.619368000 | 1.302399000 | 3.359806000 |
| H | 2.377279000 | 1.822202000 | 4.280911000 |
| C | 1.622250000 | 1.040962000 | 2.421720000 |
| H | 0.595856000 | 1.351045000 | 2.605656000 |
| C | 1.948073000 | 0.378956000 | 1.241165000 |
| H | 0.816636000 | -0.511642000 | 0.967057000 |
| C | -1.566629000 | 2.998184000 | -0.926334000 |
| C | -1.092895000 | 1.732224000 | -0.530699000 |
| C | 0.297191000 | 1.449849000 | -0.620096000 |
| C | 1.146783000 | 2.459546000 | -1.096025000 |
| C | 0.657096000 | 3.700919000 | -1.482015000 |
| C | -0.709012000 | 3.979306000 | -1.396272000 |
| H | -2.632343000 | 3.203336000 | -0.867940000 |
| H | 2.214251000 | 2.262615000 | -1.170810000 |
| H | 1.341148000 | 4.457135000 | -1.856579000 |
| H | -1.090429000 | 4.948931000 | -1.700266000 |

52

M062X_**3b** (E = -1204.514213 a.u.)

| | | | |
|---|---|---|---|
| C | -3.991220000 | -0.668217000 | 0.316456000 |
| C | -0.430327000 | -1.569282000 | -0.081726000 |
| B | 0.745748000 | -0.656694000 | -0.575238000 |
| B | -2.025074000 | 0.488753000 | -0.475153000 |
| C | 2.442952000 | -2.323225000 | -1.491441000 |
| H | 1.606118000 | -2.769344000 | -2.024948000 |
| C | -1.683226000 | -0.965387000 | -0.047856000 |
| C | -0.349446000 | -2.896671000 | 0.382027000 |
| H | 0.606983000 | -3.413914000 | 0.395907000 |
| C | -1.493730000 | -3.554526000 | 0.831794000 |
| H | -1.414843000 | -4.579137000 | 1.183243000 |
| C | -2.748867000 | -2.925327000 | 0.851718000 |
| H | -3.618902000 | -3.467452000 | 1.213395000 |
| C | -2.837366000 | -1.611876000 | 0.409389000 |
| C | -3.572866000 | 0.593452000 | -0.195714000 |
| C | -5.312063000 | -0.901603000 | 0.659146000 |
| H | -5.627117000 | -1.865933000 | 1.048109000 |
| C | -6.246300000 | 0.128659000 | 0.496486000 |
| H | -7.285152000 | -0.044233000 | 0.761201000 |
| C | -5.858889000 | 1.368087000 | 0.001033000 |
| H | -6.595477000 | 2.155970000 | -0.118462000 |
| C | -4.521916000 | 1.598837000 | -0.343876000 |
| H | -4.235696000 | 2.574204000 | -0.728052000 |
| C | 2.207142000 | -1.234022000 | -0.639327000 |
| C | 3.723182000 | -2.832682000 | -1.688295000 |
| H | 3.879318000 | -3.666423000 | -2.365524000 |
| C | 4.798950000 | -2.271879000 | -1.006064000 |
| H | 5.799537000 | -2.670538000 | -1.140663000 |
| C | 4.585979000 | -1.212741000 | -0.130407000 |
| H | 5.416967000 | -0.800296000 | 0.435419000 |
| C | 3.303624000 | -0.688883000 | 0.061747000 |
| C | 3.092248000 | 0.418918000 | 1.027451000 |
| C | 3.969770000 | 1.507398000 | 1.083412000 |
| H | 4.815733000 | 1.543512000 | 0.402413000 |
| C | 3.746432000 | 2.553751000 | 1.971763000 |
| H | 4.429758000 | 3.396845000 | 1.994077000 |
| C | 2.642462000 | 2.529000000 | 2.821527000 |
| H | 2.467269000 | 3.347508000 | 3.512088000 |
| C | 1.766018000 | 1.448764000 | 2.779876000 |
| H | 0.907614000 | 1.416457000 | 3.443017000 |
| C | 1.992843000 | 0.400560000 | 1.893914000 |
| H | 1.324384000 | -0.457853000 | 1.892326000 |
| C | -1.096243000 | 2.676959000 | -1.508239000 |
| C | -0.887244000 | 1.379701000 | -1.029747000 |
| C | 0.424334000 | 0.814362000 | -1.080168000 |
| C | 1.452978000 | 1.588674000 | -1.628194000 |
| C | 1.223751000 | 2.880434000 | -2.102332000 |
| C | -0.051894000 | 3.430058000 | -2.041124000 |
| H | -2.094440000 | 3.105107000 | -1.468955000 |
| H | 2.456797000 | 1.176593000 | -1.690342000 |
| H | 2.043855000 | 3.455700000 | -2.521702000 |
| H | -0.232467000 | 4.435166000 | -2.409968000 |

64

ωB97XD_**2a** (E = -1304.491995 a.u.)

| | | | |
|---|---|---|---|
| H | -1.685704000 | -1.708345000 | 1.635607000 |
| B | -2.402087000 | 1.034531000 | -0.994320000 |
| C | 1.867605000 | -1.152075000 | -1.443993000 |
| B | -0.485910000 | 1.130211000 | 1.027132000 |
| H | -1.684770000 | 1.709594000 | -1.635704000 |
| H | -3.103054000 | -0.631434000 | -2.791309000 |
| B | -0.486951000 | -1.129900000 | -1.027296000 |
| C | -0.438285000 | 3.692735000 | 0.096527000 |
| H | -1.501847000 | 3.772193000 | -0.097389000 |
| C | -1.927513000 | -0.586496000 | -0.592066000 |
| B | -3.279381000 | -1.629566000 | -0.411749000 |
| H | -4.736667000 | 1.774373000 | -1.728857000 |
| C | 0.094167000 | -2.537591000 | -0.661380000 |
| B | -3.278086000 | 1.632045000 | 0.412026000 |
| H | -5.699640000 | 1.075539000 | 1.108030000 |
| H | -5.700569000 | -1.071284000 | -1.107445000 |
| C | -1.926991000 | 0.588015000 | 0.592106000 |
| B | -2.402661000 | -1.032714000 | 0.994419000 |
| C | 0.393703000 | -4.763041000 | 0.233250000 |
| H | -0.027905000 | -5.663416000 | 0.668129000 |
| H | -4.737718000 | -1.770925000 | 1.729330000 |
| B | -4.157709000 | 1.032813000 | -1.003566000 |
| H | -3.102092000 | 0.633881000 | 2.791522000 |
| B | -4.707932000 | -0.616447000 | -0.638751000 |
| C | 1.763853000 | -4.675481000 | 0.010975000 |
| H | 2.407214000 | -5.509026000 | 0.276790000 |
| B | -3.258160000 | -0.363476000 | -1.646312000 |
| C | 2.323541000 | -3.522471000 | -0.547048000 |
| H | 3.396215000 | -3.457705000 | -0.702699000 |
| B | -4.158291000 | -1.029731000 | 1.004051000 |
| H | -3.148024000 | 2.763626000 | 0.735065000 |
| C | 1.488089000 | -2.470924000 | -0.881565000 |
| B | -3.257653000 | 0.365915000 | 1.646591000 |
| H | -3.150160000 | -2.761239000 | -0.734824000 |
| C | 0.725011000 | -0.333343000 | -1.627263000 |
| B | -4.707380000 | 0.619952000 | 0.639274000 |
| C | 3.138962000 | -0.696397000 | -1.738938000 |
| H | 4.011187000 | -1.320494000 | -1.569606000 |
| C | 3.291812000 | 0.596116000 | -2.251893000 |
| H | 4.285917000 | 0.968796000 | -2.480115000 |
| C | 2.184120000 | 1.402700000 | -2.475672000 |
| H | 2.310924000 | 2.402319000 | -2.877657000 |
| C | 0.902735000 | 0.939167000 | -2.165570000 |
| H | 0.059305000 | 1.594267000 | -2.338620000 |
| C | 0.096251000 | 2.537479000 | 0.661182000 |
| C | 0.397561000 | 4.762492000 | -0.233970000 |
| H | -0.023333000 | 5.663095000 | -0.669068000 |
| C | 1.767635000 | 4.673896000 | -0.011682000 |
| H | 2.411679000 | 5.506846000 | -0.277711000 |
| C | 2.326405000 | 3.520576000 | 0.546620000 |
| H | 3.399029000 | 3.454988000 | 0.702260000 |
| C | 1.490124000 | 2.469765000 | 0.881379000 |
| C | 1.868651000 | 1.150711000 | 1.443975000 |
| C | 3.139651000 | 0.694313000 | 1.739394000 |
| H | 4.012290000 | 1.317902000 | 1.570305000 |
| C | 3.291553000 | -0.598254000 | 2.252483000 |
| H | 4.285368000 | -0.971543000 | 2.480966000 |
| C | 2.183303000 | -1.404131000 | 2.476014000 |
| H | 2.309397000 | -2.403778000 | 2.878157000 |
| C | 0.902272000 | -0.939809000 | 2.165604000 |
| H | 0.058375000 | -1.594339000 | 2.338523000 |
| C | 0.725494000 | 0.332758000 | 1.627105000 |
| H | -1.504908000 | -3.771125000 | 0.097036000 |
| C | -0.441291000 | -3.692530000 | -0.096945000 |

64

ωB97XD_TS1-**2a/3a** (E = -1304.445008 a.u.)

| | | | |
|---|---|---|---|
| H | -0.427245000 | 1.404341000 | 2.246144000 |
| B | -0.503023000 | 1.564474000 | -1.584556000 |
| C | -3.690406000 | -1.631677000 | 0.219113000 |
| C | 0.000199000 | -1.532538000 | 0.239302000 |
| B | 1.125183000 | -0.188401000 | -0.015577000 |
| H | -0.638125000 | 0.679179000 | -2.358202000 |
| H | -2.883212000 | 2.490896000 | -1.674968000 |
| B | -2.083919000 | 0.128084000 | 0.152548000 |
| C | 1.879000000 | -0.944516000 | -2.503283000 |
| H | 0.877996000 | -0.837066000 | -2.908686000 |
| C | -1.299522000 | 1.445225000 | -0.081546000 |
| B | -1.750654000 | 2.931693000 | 0.601437000 |
| H | -0.365240000 | 3.715980000 | -2.995496000 |
| C | -1.353983000 | -1.232105000 | 0.268618000 |
| B | 1.023354000 | 2.397787000 | -1.238382000 |
| H | 1.360753000 | 4.917489000 | -0.753976000 |
| H | -1.690097000 | 5.199940000 | -0.649359000 |
| C | 0.378912000 | 1.264716000 | -0.134382000 |
| B | -0.363886000 | 2.004960000 | 1.228165000 |
| C | 0.385322000 | -2.885578000 | 0.203905000 |
| H | 1.439953000 | -3.145956000 | 0.189553000 |
| H | -0.129102000 | 4.482000000 | 1.899129000 |
| B | -0.364473000 | 3.303942000 | -1.881171000 |
| H | 2.132207000 | 2.549991000 | 1.085212000 |
| B | -1.130338000 | 4.159944000 | -0.515088000 |
| C | -0.580158000 | -3.883796000 | 0.187239000 |
| H | -0.268457000 | -4.923149000 | 0.156199000 |
| B | -1.836495000 | 2.661085000 | -1.141964000 |
| C | -1.949027000 | -3.577338000 | 0.202935000 |
| H | -2.683785000 | -4.376555000 | 0.179069000 |
| B | -0.225575000 | 3.750056000 | 0.968144000 |
| H | 1.986742000 | 2.082466000 | -1.849623000 |
| C | -2.330170000 | -2.246829000 | 0.237157000 |
| B | 1.108519000 | 2.671988000 | 0.503699000 |
| H | -2.737856000 | 2.946814000 | 1.260991000 |
| C | -3.595519000 | -0.217124000 | 0.175475000 |
| B | 0.633102000 | 3.994916000 | -0.575183000 |
| C | -4.921987000 | -2.260196000 | 0.231243000 |
| H | -5.004366000 | -3.342771000 | 0.261990000 |
| C | -6.078793000 | -1.469865000 | 0.204701000 |
| H | -7.051819000 | -1.951400000 | 0.217121000 |
| C | -6.000586000 | -0.082047000 | 0.160224000 |
| H | -6.910721000 | 0.508369000 | 0.137755000 |
| C | -4.753192000 | 0.550092000 | 0.141525000 |
| H | -4.687474000 | 1.633457000 | 0.100191000 |
| C | 2.127448000 | -0.670313000 | -1.160424000 |
| C | 2.919264000 | -1.348083000 | -3.336556000 |
| H | 2.726177000 | -1.543181000 | -4.386864000 |
| C | 4.210446000 | -1.504107000 | -2.829967000 |
| H | 5.013317000 | -1.822104000 | -3.487816000 |
| C | 4.471392000 | -1.261048000 | -1.486293000 |
| H | 5.473873000 | -1.398621000 | -1.091303000 |
| C | 3.428450000 | -0.844716000 | -0.660404000 |
| C | 3.487342000 | -0.559946000 | 0.779986000 |
| C | 4.612736000 | -0.540467000 | 1.599009000 |
| H | 5.594280000 | -0.757660000 | 1.188975000 |
| C | 4.474305000 | -0.228036000 | 2.948824000 |
| H | 5.351739000 | -0.210671000 | 3.587978000 |
| C | 3.222171000 | 0.059889000 | 3.487150000 |
| H | 3.122948000 | 0.292728000 | 4.542058000 |
| C | 2.093715000 | 0.047363000 | 2.670685000 |
| H | 1.120543000 | 0.259604000 | 3.100025000 |
| C | 2.220804000 | -0.243279000 | 1.313282000 |
| H | 0.969012000 | -0.978849000 | 1.028422000 |

| | | | |
|---|---|---|---|
| 64 | | | |
| ωB97XD_**3a** (E = -1304.502156 a.u.) | | | |
| H | -0.127483000 | 1.114217000 | 1.830602000 |
| B | -0.534850000 | 1.462531000 | -1.976628000 |
| C | -3.950859000 | -1.309275000 | 0.300669000 |
| C | -0.326823000 | -1.648966000 | -0.376622000 |
| B | 0.787474000 | -0.579518000 | -0.611548000 |
| H | -0.860463000 | 0.632036000 | -2.757458000 |
| H | -2.732807000 | 2.766358000 | -1.820027000 |
| B | -2.175101000 | 0.224435000 | -0.129999000 |
| C | 2.358694000 | -1.517804000 | -2.338804000 |
| H | 1.479725000 | -1.576722000 | -2.977014000 |
| C | -1.216998000 | 1.418214000 | -0.402918000 |
| B | -1.351009000 | 2.923026000 | 0.356277000 |
| H | -0.169808000 | 3.621605000 | -3.332344000 |
| C | -1.629788000 | -1.219480000 | -0.143451000 |
| B | 1.134944000 | 2.030834000 | -1.747005000 |
| H | 1.912384000 | 4.443593000 | -1.227492000 |
| H | -1.023725000 | 5.200096000 | -0.836742000 |
| C | 0.403408000 | 0.977537000 | -0.616668000 |
| B | -0.086262000 | 1.756080000 | 0.837246000 |
| C | -0.100983000 | -3.040574000 | -0.356424000 |
| H | 0.897770000 | -3.430690000 | -0.531709000 |
| H | 0.616487000 | 4.133212000 | 1.538994000 |
| B | -0.139189000 | 3.167179000 | -2.235218000 |
| H | 2.441089000 | 1.903402000 | 0.471277000 |
| B | -0.633901000 | 4.078905000 | -0.779890000 |
| C | -1.150300000 | -3.920188000 | -0.109884000 |
| H | -0.957430000 | -4.988675000 | -0.096739000 |
| B | -1.631657000 | 2.741959000 | -1.378381000 |
| C | -2.458012000 | -3.462441000 | 0.123027000 |
| H | -3.253846000 | -4.177852000 | 0.310655000 |
| B | 0.317043000 | 3.465038000 | 0.603603000 |
| H | 1.973278000 | 1.576694000 | -2.450121000 |
| C | -2.695736000 | -2.097768000 | 0.103979000 |
| B | 1.411611000 | 2.212177000 | -0.015429000 |
| H | -2.260717000 | 3.073904000 | 1.103432000 |
| C | -3.691277000 | 0.081991000 | 0.166000000 |
| B | 1.065119000 | 3.639799000 | -1.007395000 |
| C | -5.226151000 | -1.766990000 | 0.574638000 |
| H | -5.433445000 | -2.827721000 | 0.681487000 |
| C | -6.260451000 | -0.831306000 | 0.716539000 |
| H | -7.266281000 | -1.179740000 | 0.931030000 |
| C | -6.021099000 | 0.531714000 | 0.585129000 |
| H | -6.837695000 | 1.237377000 | 0.697514000 |
| C | -4.729529000 | 0.993139000 | 0.308323000 |
| H | -4.539268000 | 2.057403000 | 0.203448000 |
| C | 2.229488000 | -1.031031000 | -1.029445000 |
| C | 3.592020000 | -1.904798000 | -2.850490000 |
| H | 3.669028000 | -2.262403000 | -3.872290000 |
| C | 4.721616000 | -1.839198000 | -2.040762000 |
| H | 5.687743000 | -2.152250000 | -2.423853000 |
| C | 4.607567000 | -1.388831000 | -0.731504000 |
| H | 5.483156000 | -1.371625000 | -0.088956000 |
| C | 3.374727000 | -0.980121000 | -0.212557000 |
| C | 3.282075000 | -0.511324000 | 1.193375000 |
| C | 4.241937000 | 0.363561000 | 1.716639000 |
| H | 5.061925000 | 0.692340000 | 1.085272000 |
| C | 4.118410000 | 0.864097000 | 3.006242000 |
| H | 4.858822000 | 1.561394000 | 3.385300000 |
| C | 3.035524000 | 0.494643000 | 3.802146000 |
| H | 2.932060000 | 0.897915000 | 4.804275000 |
| C | 2.089858000 | -0.394086000 | 3.303815000 |
| H | 1.251327000 | -0.702089000 | 3.920538000 |
| C | 2.216956000 | -0.899265000 | 2.012472000 |
| H | 1.491423000 | -1.622690000 | 1.651968000 |

| | | | |
|---|---|---|---|
| 52 | | | |
| ωB97XD_**2b** (E = -1204.612990 a.u.) | | | |
| C | 1.932513000 | -1.341403000 | 1.355123000 |
| B | -1.442675000 | 0.874441000 | -0.543661000 |
| B | 1.442348000 | 0.873712000 | 0.543440000 |
| C | -3.862693000 | 1.069906000 | 0.667450000 |
| H | -3.692783000 | 2.069309000 | 1.060832000 |
| C | 2.888605000 | 0.448968000 | 0.105474000 |
| C | 5.061191000 | 0.403607000 | -0.945680000 |
| H | 5.828174000 | 0.886426000 | -1.543230000 |
| C | 5.269478000 | -0.883330000 | -0.460587000 |
| H | 6.201123000 | -1.395731000 | -0.682167000 |
| C | 4.292761000 | -1.531278000 | 0.305066000 |
| H | 4.467454000 | -2.540318000 | 0.667753000 |
| C | 3.112283000 | -0.863193000 | 0.583422000 |
| C | 0.931835000 | -0.344533000 | 1.388287000 |
| C | 1.742949000 | -2.563919000 | 1.976214000 |
| H | 2.506088000 | -3.336397000 | 1.946340000 |
| C | 0.536704000 | -2.796821000 | 2.647976000 |
| H | 0.373606000 | -3.754034000 | 3.134434000 |
| C | -0.450685000 | -1.819369000 | 2.705241000 |
| H | -1.378573000 | -2.016026000 | 3.232704000 |
| C | -0.249026000 | -0.584837000 | 2.080170000 |
| H | -1.022501000 | 0.177107000 | 2.131209000 |
| C | -2.888807000 | 0.449527000 | -0.105481000 |
| C | -5.060998000 | 0.403621000 | 0.946470000 |
| H | -5.827843000 | 0.886218000 | 1.544378000 |
| C | -5.269201000 | -0.883317000 | 0.461342000 |
| H | -6.200659000 | -1.395923000 | 0.683237000 |
| C | -4.292634000 | -1.530993000 | -0.304735000 |
| H | -4.467246000 | -2.540031000 | -0.667464000 |
| C | -3.112399000 | -0.862639000 | -0.583472000 |
| C | -1.932779000 | -1.340613000 | -1.355541000 |
| C | -1.743161000 | -2.563061000 | -1.976736000 |
| H | -2.506170000 | -3.335663000 | -1.946752000 |
| C | -0.537013000 | -2.795737000 | -2.648756000 |
| H | -0.373860000 | -3.752914000 | -3.135268000 |
| C | 0.450219000 | -1.818139000 | -2.706183000 |
| H | 1.378026000 | -2.014655000 | -3.233841000 |
| C | 0.248512000 | -0.583670000 | -2.080998000 |
| H | 1.021856000 | 0.178402000 | -2.132117000 |
| C | -0.932246000 | -0.343598000 | -1.388857000 |
| H | 3.692642000 | 2.069019000 | -1.060399000 |
| C | 3.862645000 | 1.069619000 | -0.667046000 |
| C | 1.331900000 | 3.414835000 | 0.385746000 |
| C | 0.680570000 | 2.187332000 | 0.203070000 |
| C | -0.680548000 | 2.187801000 | -0.203115000 |
| C | -1.331259000 | 3.415688000 | -0.385149000 |
| C | -0.667051000 | 4.624771000 | -0.193684000 |
| C | 0.668324000 | 4.624344000 | 0.194833000 |
| H | 2.373651000 | 3.424116000 | 0.697909000 |
| H | -2.373030000 | 3.425692000 | -0.697223000 |
| H | -1.190238000 | 5.563990000 | -0.346051000 |
| H | 1.191963000 | 5.563242000 | 0.347625000 |

52

ωB97XD_TS1-**2b**/**3b** (E = -1204.558240 a.u.)

| | | | |
|---|---|---|---|
| C | -3.784105000 | -1.019414000 | 0.227679000 |
| C | -0.107061000 | -1.211044000 | 0.178587000 |
| B | 1.028634000 | 0.062209000 | -0.210383000 |
| B | -2.022785000 | 0.599729000 | -0.088230000 |
| C | 2.209730000 | -1.118985000 | -2.328961000 |
| H | 1.287694000 | -1.115080000 | -2.904152000 |
| C | -1.435475000 | -0.813611000 | 0.182665000 |
| C | 0.185328000 | -2.581193000 | 0.307235000 |
| H | 1.217894000 | -2.918498000 | 0.320805000 |
| C | -0.852061000 | -3.500755000 | 0.410120000 |
| H | -0.619454000 | -4.557429000 | 0.501129000 |
| C | -2.196155000 | -3.094838000 | 0.399685000 |
| H | -2.984076000 | -3.838847000 | 0.477606000 |
| C | -2.482042000 | -1.743559000 | 0.283094000 |
| C | -3.578105000 | 0.373525000 | 0.037343000 |
| C | -5.057959000 | -1.550900000 | 0.340985000 |
| H | -5.209821000 | -2.617297000 | 0.482203000 |
| C | -6.157835000 | -0.688991000 | 0.277585000 |
| H | -7.161235000 | -1.093693000 | 0.370794000 |
| C | -5.978042000 | 0.678148000 | 0.100369000 |
| H | -6.841048000 | 1.335338000 | 0.057176000 |
| C | -4.689239000 | 1.207553000 | -0.022978000 |
| H | -4.566736000 | 2.278140000 | -0.160830000 |
| C | 2.227950000 | -0.584305000 | -1.042980000 |
| C | 3.372166000 | -1.654920000 | -2.878883000 |
| H | 3.358718000 | -2.063166000 | -3.885092000 |
| C | 4.557606000 | -1.674406000 | -2.141275000 |
| H | 5.456828000 | -2.099362000 | -2.576891000 |
| C | 4.592892000 | -1.156272000 | -0.850861000 |
| H | 5.516425000 | -1.180630000 | -0.278838000 |
| C | 3.428630000 | -0.611976000 | -0.309637000 |
| C | 3.250223000 | -0.008576000 | 1.021234000 |
| C | 4.218396000 | 0.271202000 | 1.982833000 |
| H | 5.257914000 | 0.005412000 | 1.814227000 |
| C | 3.846816000 | 0.916032000 | 3.160745000 |
| H | 4.600646000 | 1.136157000 | 3.910540000 |
| C | 2.522385000 | 1.292102000 | 3.385037000 |
| H | 2.251667000 | 1.800282000 | 4.304943000 |
| C | 1.550446000 | 1.016333000 | 2.426081000 |
| H | 0.514680000 | 1.302254000 | 2.591310000 |
| C | 1.912153000 | 0.366212000 | 1.249624000 |
| H | 0.801901000 | -0.531815000 | 0.950221000 |
| C | -1.552132000 | 2.989171000 | -0.946336000 |
| C | -1.082986000 | 1.721212000 | -0.550663000 |
| C | 0.306251000 | 1.433886000 | -0.644068000 |
| C | 1.157897000 | 2.439551000 | -1.123653000 |
| C | 0.671944000 | 3.681975000 | -1.509144000 |
| C | -0.692003000 | 3.965828000 | -1.419423000 |
| H | -2.616006000 | 3.201013000 | -0.884606000 |
| H | 2.223901000 | 2.241118000 | -1.203227000 |
| H | 1.358722000 | 4.435437000 | -1.884793000 |
| H | -1.071532000 | 4.937065000 | -1.721701000 |

52

ωB97XD_**3b** (E = -1204.614720 a.u.)

| | | | |
|---|---|---|---|
| C | -3.958707000 | -0.693975000 | 0.299040000 |
| C | -0.401778000 | -1.593150000 | -0.124292000 |
| B | 0.770918000 | -0.672476000 | -0.607882000 |
| B | -1.995690000 | 0.470444000 | -0.485136000 |
| C | 2.537532000 | -2.305671000 | -1.480571000 |
| H | 1.735662000 | -2.760190000 | -2.057995000 |
| C | -1.653777000 | -0.988686000 | -0.078714000 |
| C | -0.320014000 | -2.924255000 | 0.326792000 |
| H | 0.635608000 | -3.442122000 | 0.330589000 |
| C | -1.461796000 | -3.585585000 | 0.776025000 |
| H | -1.381261000 | -4.613764000 | 1.117172000 |
| C | -2.715437000 | -2.954702000 | 0.809039000 |
| H | -3.584041000 | -3.498079000 | 1.171729000 |
| C | -2.804823000 | -1.638170000 | 0.379143000 |
| C | -3.541254000 | 0.571518000 | -0.202443000 |
| C | -5.279625000 | -0.930425000 | 0.637685000 |
| H | -5.594800000 | -1.897437000 | 1.019785000 |
| C | -6.214778000 | 0.099272000 | 0.480187000 |
| H | -7.254518000 | -0.076986000 | 0.739522000 |
| C | -5.827751000 | 1.342055000 | -0.006897000 |
| H | -6.565570000 | 2.129697000 | -0.123601000 |
| C | -4.490798000 | 1.576562000 | -0.346787000 |
| H | -4.204736000 | 2.553313000 | -0.727374000 |
| C | 2.243455000 | -1.228078000 | -0.632306000 |
| C | 3.833041000 | -2.793704000 | -1.618347000 |
| H | 4.035278000 | -3.618424000 | -2.294792000 |
| C | 4.865352000 | -2.222277000 | -0.880552000 |
| H | 5.877787000 | -2.603796000 | -0.970860000 |
| C | 4.593525000 | -1.172977000 | -0.010013000 |
| H | 5.391232000 | -0.748715000 | 0.593515000 |
| C | 3.296070000 | -0.669714000 | 0.122853000 |
| C | 3.021987000 | 0.440527000 | 1.071065000 |
| C | 3.837111000 | 1.576808000 | 1.100417000 |
| H | 4.686214000 | 1.638885000 | 0.425458000 |
| C | 3.545916000 | 2.637321000 | 1.950992000 |
| H | 4.181234000 | 3.517856000 | 1.950982000 |
| C | 2.434967000 | 2.579089000 | 2.789380000 |
| H | 2.204162000 | 3.409742000 | 3.448829000 |
| C | 1.622845000 | 1.449292000 | 2.777111000 |
| H | 0.759731000 | 1.390519000 | 3.432743000 |
| C | 1.918016000 | 0.386791000 | 1.929120000 |
| H | 1.293764000 | -0.503138000 | 1.945386000 |
| C | -1.068308000 | 2.675297000 | -1.489449000 |
| C | -0.862100000 | 1.368858000 | -1.035192000 |
| C | 0.445179000 | 0.796820000 | -1.114564000 |
| C | 1.468189000 | 1.569456000 | -1.674547000 |
| C | 1.240569000 | 2.868109000 | -2.127618000 |
| C | -0.027683000 | 3.427674000 | -2.029841000 |
| H | -2.060043000 | 3.114256000 | -1.423735000 |
| H | 2.466875000 | 1.151186000 | -1.759891000 |
| H | 2.057368000 | 3.442174000 | -2.555126000 |
| H | -0.206820000 | 4.440698000 | -2.377553000 |

64

B3LYP(D3bj)_**2a** (E = -1305.062609 a.u.)

| | | | |
|---|---|---|---|
| H | -1.682754000 | -1.665185000 | 1.670280000 |
| B | -2.399629000 | 1.008795000 | -1.014170000 |
| C | 1.872121000 | -1.183944000 | -1.441671000 |
| B | -0.483638000 | 1.139021000 | 0.994873000 |
| H | -1.684064000 | 1.663502000 | -1.670101000 |
| H | -3.100396000 | -0.696848000 | -2.773636000 |
| B | -0.482348000 | -1.139507000 | -0.994681000 |
| C | -0.452458000 | 3.696325000 | 0.053238000 |
| H | -1.512950000 | 3.758136000 | -0.158379000 |
| C | -1.922319000 | -0.604042000 | -0.580396000 |
| B | -3.275633000 | -1.641496000 | -0.375726000 |
| H | -4.730469000 | 1.730979000 | -1.768125000 |
| C | 0.091775000 | -2.545623000 | -0.628921000 |
| B | -3.277274000 | 1.638218000 | 0.375573000 |
| H | -5.695113000 | 1.093046000 | 1.081415000 |
| H | -5.693871000 | -1.098635000 | -1.081995000 |
| C | -1.923001000 | 0.602048000 | 0.580453000 |
| B | -2.398831000 | -1.011229000 | 1.014166000 |
| C | 0.380457000 | -4.780453000 | 0.265474000 |
| H | -0.044875000 | -5.675054000 | 0.709465000 |
| H | -4.729103000 | -1.735618000 | 1.767718000 |
| B | -4.152887000 | 1.008301000 | -1.027995000 |
| H | -3.101551000 | 0.693753000 | 2.773497000 |
| B | -4.703388000 | -0.635071000 | -0.625847000 |
| C | 1.751036000 | -4.711401000 | 0.017508000 |
| H | 2.388269000 | -5.553411000 | 0.272207000 |
| B | -3.253021000 | -0.403169000 | -1.638152000 |
| C | 2.317491000 | -3.562953000 | -0.553953000 |
| H | 3.388194000 | -3.513419000 | -0.728381000 |
| B | -4.152096000 | -1.012395000 | 1.027671000 |
| H | -3.151629000 | 2.773528000 | 0.672130000 |
| C | 1.489914000 | -2.497010000 | -0.875686000 |
| B | -3.253687000 | 0.399925000 | 1.637987000 |
| H | -3.148819000 | -2.776679000 | -0.672251000 |
| C | 0.727916000 | -0.347413000 | -1.595313000 |
| B | -4.704110000 | 0.630437000 | 0.625431000 |
| C | 3.143448000 | -0.739046000 | -1.766225000 |
| H | 4.011275000 | -1.375788000 | -1.622712000 |
| C | 3.300392000 | 0.559528000 | -2.274325000 |
| H | 4.293595000 | 0.921056000 | -2.524714000 |
| C | 2.194507000 | 1.383413000 | -2.463330000 |
| H | 2.323413000 | 2.386471000 | -2.856455000 |
| C | 0.911419000 | 0.930115000 | -2.129362000 |
| H | 0.072968000 | 1.595978000 | -2.277005000 |
| C | 0.088972000 | 2.545714000 | 0.629014000 |
| C | 0.375330000 | 4.780950000 | -0.265076000 |
| H | -0.050936000 | 5.675158000 | -0.708965000 |
| C | 1.745971000 | 4.713315000 | -0.017063000 |
| H | 2.382325000 | 5.556029000 | -0.271633000 |
| C | 2.313612000 | 3.565371000 | 0.554248000 |
| H | 3.384359000 | 3.516921000 | 0.728710000 |
| C | 1.487140000 | 2.498522000 | 0.875802000 |
| C | 1.870703000 | 1.185799000 | 1.441699000 |
| C | 3.142525000 | 0.742149000 | 1.766016000 |
| H | 4.009699000 | 1.379753000 | 1.622377000 |
| C | 3.300872000 | -0.556317000 | 2.273956000 |
| H | 4.294488000 | -0.916850000 | 2.524144000 |
| C | 2.195867000 | -1.381359000 | 2.463042000 |
| H | 2.325859000 | -2.384314000 | 2.856073000 |
| C | 0.912273000 | -0.929325000 | 2.129335000 |
| H | 0.074513000 | -1.596031000 | 2.277076000 |
| C | 0.727365000 | 0.348062000 | 1.595416000 |
| H | -1.508896000 | -3.759674000 | 0.158523000 |
| C | -0.448464000 | -3.696751000 | -0.053031000 |

64

B3LYP(D3bj)_TS1-**2a/3a** (E = -1305.011695 a.u.)

| | | | |
|---|---|---|---|
| H | -0.402181000 | 1.455954000 | 2.233658000 |
| B | -0.531938000 | 1.535375000 | -1.583443000 |
| C | -3.686519000 | -1.644200000 | 0.256708000 |
| C | 0.007164000 | -1.516859000 | 0.263408000 |
| B | 1.125354000 | -0.171838000 | -0.008559000 |
| H | -0.674131000 | 0.637336000 | -2.334165000 |
| H | -2.911334000 | 2.457912000 | -1.671891000 |
| B | -2.089107000 | 0.129983000 | 0.178542000 |
| C | 1.840420000 | -0.976849000 | -2.488853000 |
| H | 0.835947000 | -0.859332000 | -2.881014000 |
| C | -1.317698000 | 1.446182000 | -0.073053000 |
| B | -1.756326000 | 2.946818000 | 0.582820000 |
| H | -0.421817000 | 3.650892000 | -3.047381000 |
| C | -1.351037000 | -1.221431000 | 0.296536000 |
| B | 0.995972000 | 2.382222000 | -1.281793000 |
| H | 1.328816000 | 4.910746000 | -0.854709000 |
| H | -1.717860000 | 5.185437000 | -0.715564000 |
| C | 0.375561000 | 1.270043000 | -0.145925000 |
| B | -0.357912000 | 2.031895000 | 1.205157000 |
| C | 0.403541000 | -2.871779000 | 0.243667000 |
| H | 1.458906000 | -3.125734000 | 0.228281000 |
| H | -0.121625000 | 4.521124000 | 1.826976000 |
| B | -0.404621000 | 3.265568000 | -1.927009000 |
| H | 2.128977000 | 2.589184000 | 1.023278000 |
| B | -1.154353000 | 4.153154000 | -0.567817000 |
| C | -0.557471000 | -3.878696000 | 0.242135000 |
| H | -0.238992000 | -4.916690000 | 0.222869000 |
| B | -1.864131000 | 2.639521000 | -1.152719000 |
| C | -1.933219000 | -3.580558000 | 0.257376000 |
| H | -2.661567000 | -4.386095000 | 0.242785000 |
| B | -0.229163000 | 3.772931000 | 0.914321000 |
| H | 1.947736000 | 2.059281000 | -1.899337000 |
| C | -2.325676000 | -2.249897000 | 0.278916000 |
| B | 1.102874000 | 2.692862000 | 0.451349000 |
| H | -2.729434000 | 2.976801000 | 1.255911000 |
| C | -3.597489000 | -0.221557000 | 0.207076000 |
| B | 0.609173000 | 3.991715000 | -0.648365000 |
| C | -4.918377000 | -2.279709000 | 0.264817000 |
| H | -4.993548000 | -3.362892000 | 0.295796000 |
| C | -6.083101000 | -1.494683000 | 0.229645000 |
| H | -7.053629000 | -1.982185000 | 0.237022000 |
| C | -6.011836000 | -0.102907000 | 0.181917000 |
| H | -6.924880000 | 0.483273000 | 0.153035000 |
| C | -4.764499000 | 0.537862000 | 0.166712000 |
| H | -4.708006000 | 1.621082000 | 0.122898000 |
| C | 2.111821000 | -0.679995000 | -1.152801000 |
| C | 2.864458000 | -1.415684000 | -3.330695000 |
| H | 2.653062000 | -1.626533000 | -4.374804000 |
| C | 4.162819000 | -1.586853000 | -2.837336000 |
| H | 4.951477000 | -1.931666000 | -3.499416000 |
| C | 4.447523000 | -1.323313000 | -1.498778000 |
| H | 5.452932000 | -1.470655000 | -1.115057000 |
| C | 3.422076000 | -0.871088000 | -0.662850000 |
| C | 3.508492000 | -0.554574000 | 0.766413000 |
| C | 4.651343000 | -0.533520000 | 1.569152000 |
| H | 5.619669000 | -0.788870000 | 1.149712000 |
| C | 4.545154000 | -0.169565000 | 2.912251000 |
| H | 5.435036000 | -0.150569000 | 3.534398000 |
| C | 3.307455000 | 0.171729000 | 3.463580000 |
| H | 3.235505000 | 0.450711000 | 4.509879000 |
| C | 2.159420000 | 0.152472000 | 2.667872000 |
| H | 1.199533000 | 0.404245000 | 3.104218000 |
| C | 2.254810000 | -0.191408000 | 1.318107000 |
| H | 0.991489000 | -0.932768000 | 1.064237000 |

64

B3LYP(D3bj)_**3a** (E = -1305.071413 a.u.)

| | | | |
|---|---|---|---|
| H | -0.206371000 | 1.165523000 | 1.828978000 |
| B | -0.484754000 | 1.388922000 | -1.982685000 |
| C | -3.987081000 | -1.274596000 | 0.345834000 |
| C | -0.351801000 | -1.659385000 | -0.270970000 |
| B | 0.770762000 | -0.616958000 | -0.546880000 |
| H | -0.791831000 | 0.541113000 | -2.745950000 |
| H | -2.674010000 | 2.714323000 | -1.946516000 |
| B | -2.195212000 | 0.232178000 | -0.137054000 |
| C | 2.251538000 | -1.687493000 | -2.275045000 |
| H | 1.340623000 | -1.798583000 | -2.857796000 |
| C | -1.228246000 | 1.405118000 | -0.436195000 |
| B | -1.366444000 | 2.935039000 | 0.266409000 |
| H | -0.064415000 | 3.496442000 | -3.401567000 |
| C | -1.659028000 | -1.210699000 | -0.075686000 |
| B | 1.180188000 | 1.958645000 | -1.725049000 |
| H | 1.956176000 | 4.377652000 | -1.256141000 |
| H | -0.981765000 | 5.165546000 | -0.986260000 |
| C | 0.412156000 | 0.940592000 | -0.587332000 |
| B | -0.128048000 | 1.769288000 | 0.819220000 |
| C | -0.133203000 | -3.054490000 | -0.180251000 |
| H | 0.865678000 | -3.454272000 | -0.325001000 |
| H | 0.571122000 | 4.160026000 | 1.473928000 |
| B | -0.071674000 | 3.080791000 | -2.292144000 |
| H | 2.408833000 | 1.899635000 | 0.537730000 |
| B | -0.604013000 | 4.047039000 | -0.881913000 |
| C | -1.191795000 | -3.918577000 | 0.097383000 |
| H | -1.004233000 | -4.986260000 | 0.165763000 |
| B | -1.592234000 | 2.696859000 | -1.467939000 |
| C | -2.503956000 | -3.443121000 | 0.291797000 |
| H | -3.305855000 | -4.144500000 | 0.505452000 |
| B | 0.296491000 | 3.466059000 | 0.553879000 |
| H | 2.031951000 | 1.479075000 | -2.386974000 |
| C | -2.736711000 | -2.076393000 | 0.201738000 |
| B | 1.402345000 | 2.191653000 | 0.007095000 |
| H | -2.293088000 | 3.119197000 | 0.978545000 |
| C | -3.713936000 | 0.114622000 | 0.148074000 |
| B | 1.097824000 | 3.589565000 | -1.039819000 |
| C | -5.271276000 | -1.710745000 | 0.628802000 |
| H | -5.486838000 | -2.764422000 | 0.781274000 |
| C | -6.304566000 | -0.761546000 | 0.717966000 |
| H | -7.314393000 | -1.094103000 | 0.940192000 |
| C | -6.053727000 | 0.596000000 | 0.525898000 |
| H | -6.867090000 | 1.310981000 | 0.599153000 |
| C | -4.753134000 | 1.037134000 | 0.239372000 |
| H | -4.557075000 | 2.094721000 | 0.090672000 |
| C | 2.184579000 | -1.107191000 | -0.995353000 |
| C | 3.464370000 | -2.094849000 | -2.827434000 |
| H | 3.491353000 | -2.519668000 | -3.826199000 |
| C | 4.637765000 | -1.960789000 | -2.084801000 |
| H | 5.586982000 | -2.289128000 | -2.497327000 |
| C | 4.587166000 | -1.427292000 | -0.799997000 |
| H | 5.493631000 | -1.366430000 | -0.205738000 |
| C | 3.374818000 | -0.996012000 | -0.241393000 |
| C | 3.340475000 | -0.461200000 | 1.138258000 |
| C | 4.361641000 | 0.378210000 | 1.612801000 |
| H | 5.186896000 | 0.633974000 | 0.956133000 |
| C | 4.289930000 | 0.936316000 | 2.885695000 |
| H | 5.077287000 | 1.602149000 | 3.225881000 |
| C | 3.198266000 | 0.664243000 | 3.714706000 |
| H | 3.138264000 | 1.110280000 | 4.702484000 |
| C | 2.189812000 | -0.186906000 | 3.265775000 |
| H | 1.345638000 | -0.420107000 | 3.907462000 |
| C | 2.266831000 | -0.753398000 | 1.993624000 |
| H | 1.501064000 | -1.453883000 | 1.676625000 |

52

B3LYP(D3bj)_**2b** (E = -1205.152114 a.u.)

| | | | |
|---|---|---|---|
| C | 1.580164000 | -1.353433000 | 1.358457000 |
| B | -1.341114000 | 0.946254000 | -0.677871000 |
| B | 1.340988000 | 0.946106000 | 0.677699000 |
| C | -3.848603000 | 1.009088000 | 0.354970000 |
| H | -3.799699000 | 2.049232000 | 0.667726000 |
| C | 2.773617000 | 0.430836000 | 0.314998000 |
| C | 4.991447000 | 0.246414000 | -0.636744000 |
| H | 5.835095000 | 0.695648000 | -1.152197000 |
| C | 5.041504000 | -1.096944000 | -0.263777000 |
| H | 5.925635000 | -1.685223000 | -0.493146000 |
| C | 3.962359000 | -1.703054000 | 0.398249000 |
| H | 4.012476000 | -2.753727000 | 0.670095000 |
| C | 2.840079000 | -0.939255000 | 0.689761000 |
| C | 0.682037000 | -0.255221000 | 1.431656000 |
| C | 1.221166000 | -2.599693000 | 1.851409000 |
| H | 1.894809000 | -3.449252000 | 1.780291000 |
| C | -0.042691000 | -2.754210000 | 2.444723000 |
| H | -0.335493000 | -3.727367000 | 2.828834000 |
| C | -0.921221000 | -1.677219000 | 2.553705000 |
| H | -1.892384000 | -1.814234000 | 3.018980000 |
| C | -0.553401000 | -0.420482000 | 2.052643000 |
| H | -1.242904000 | 0.414093000 | 2.132234000 |
| C | -2.773707000 | 0.430918000 | -0.315116000 |
| C | -4.991401000 | 0.246321000 | 0.636914000 |
| H | -5.835032000 | 0.695499000 | 1.152443000 |
| C | -5.041360000 | -1.097068000 | 0.264046000 |
| H | -5.925395000 | -1.685430000 | 0.493572000 |
| C | -3.962230000 | -1.703107000 | -0.398073000 |
| H | -4.012260000 | -2.753808000 | -0.669826000 |
| C | -2.840075000 | -0.939204000 | -0.689786000 |
| C | -1.580173000 | -1.353317000 | -1.358550000 |
| C | -1.221117000 | -2.599562000 | -1.851496000 |
| H | -1.894699000 | -3.449165000 | -1.780333000 |
| C | 0.042725000 | -2.754007000 | -2.444864000 |
| H | 0.335573000 | -3.727153000 | -2.828967000 |
| C | 0.921182000 | -1.676962000 | -2.553909000 |
| H | 1.892330000 | -1.813931000 | -3.019228000 |
| C | 0.553305000 | -0.420240000 | -2.052852000 |
| H | 1.242742000 | 0.414384000 | -2.132488000 |
| C | -0.682119000 | -0.255055000 | -1.431811000 |
| H | 3.799557000 | 2.049198000 | -0.667816000 |
| C | 3.848536000 | 1.009080000 | -0.354986000 |
| C | 1.287270000 | 3.508125000 | 0.528693000 |
| C | 0.656431000 | 2.278649000 | 0.278827000 |
| C | -0.656548000 | 2.278769000 | -0.278948000 |
| C | -1.287268000 | 3.508353000 | -0.528550000 |
| C | -0.644990000 | 4.719678000 | -0.265163000 |
| C | 0.645098000 | 4.719564000 | 0.265567000 |
| H | 2.288246000 | 3.517018000 | 0.953105000 |
| H | -2.288234000 | 3.517428000 | -0.952983000 |
| H | -1.149138000 | 5.659309000 | -0.472542000 |
| H | 1.149324000 | 5.659108000 | 0.473156000 |

52

B3LYP(D3bj)_TS1-**2b/3b** (E = -1205.095101 a.u.)

| | | | |
|---|---|---|---|
| C | -3.799739000 | -1.007244000 | 0.246068000 |
| C | -0.118978000 | -1.231184000 | 0.177492000 |
| B | 1.022846000 | 0.027051000 | -0.219990000 |
| B | -2.021200000 | 0.591618000 | -0.100551000 |
| C | 2.236836000 | -1.196115000 | -2.292591000 |
| H | 1.319806000 | -1.218916000 | -2.875378000 |
| C | -1.447089000 | -0.819804000 | 0.185484000 |
| C | 0.164015000 | -2.606064000 | 0.323647000 |
| H | 1.192836000 | -2.952842000 | 0.333374000 |
| C | -0.884504000 | -3.516441000 | 0.446326000 |
| H | -0.660315000 | -4.574552000 | 0.546909000 |
| C | -2.229303000 | -3.098235000 | 0.440959000 |
| H | -3.022736000 | -3.834699000 | 0.535194000 |
| C | -2.507259000 | -1.742697000 | 0.307223000 |
| C | -3.574913000 | 0.387666000 | 0.032213000 |
| C | -5.084409000 | -1.519515000 | 0.368152000 |
| H | -5.250514000 | -2.581971000 | 0.524026000 |
| C | -6.176032000 | -0.642132000 | 0.291345000 |
| H | -7.185138000 | -1.032116000 | 0.389480000 |
| C | -5.978227000 | 0.723520000 | 0.092442000 |
| H | -6.833333000 | 1.390681000 | 0.038422000 |
| C | -4.679156000 | 1.235087000 | -0.039843000 |
| H | -4.543490000 | 2.302028000 | -0.193816000 |
| C | 2.237088000 | -0.627335000 | -1.018795000 |
| C | 3.413633000 | -1.730599000 | -2.823618000 |
| H | 3.413026000 | -2.160698000 | -3.821140000 |
| C | 4.597047000 | -1.715756000 | -2.076146000 |
| H | 5.505693000 | -2.138103000 | -2.495339000 |
| C | 4.616147000 | -1.163740000 | -0.795910000 |
| H | 5.535293000 | -1.160411000 | -0.216468000 |
| C | 3.438762000 | -0.619860000 | -0.272964000 |
| C | 3.248866000 | 0.019644000 | 1.035960000 |
| C | 4.215459000 | 0.345511000 | 1.991532000 |
| H | 5.257262000 | 0.082929000 | 1.831905000 |
| C | 3.836635000 | 1.030627000 | 3.147919000 |
| H | 4.588650000 | 1.285746000 | 3.888938000 |
| C | 2.504883000 | 1.403420000 | 3.358797000 |
| H | 2.228639000 | 1.944110000 | 4.258775000 |
| C | 1.532362000 | 1.080566000 | 2.408902000 |
| H | 0.494510000 | 1.363904000 | 2.564124000 |
| C | 1.902236000 | 0.391448000 | 1.255466000 |
| H | 0.790761000 | -0.532393000 | 0.961680000 |
| C | -1.537220000 | 2.961543000 | -1.003286000 |
| C | -1.077173000 | 1.693018000 | -0.584524000 |
| C | 0.315284000 | 1.391476000 | -0.682508000 |
| C | 1.173185000 | 2.380847000 | -1.190690000 |
| C | 0.694661000 | 3.622959000 | -1.598723000 |
| C | -0.669668000 | 3.922321000 | -1.502998000 |
| H | -2.598636000 | 3.183300000 | -0.939892000 |
| H | 2.235802000 | 2.169142000 | -1.272949000 |
| H | 1.384547000 | 4.362145000 | -1.997455000 |
| H | -1.041470000 | 4.891762000 | -1.821668000 |

52

B3LYP(D3bj)_**3b** (E = -1205.154418 a.u.)

| | | | |
|---|---|---|---|
| C | -3.952661000 | -0.700893000 | 0.290209000 |
| C | -0.394073000 | -1.619271000 | -0.135573000 |
| B | 0.782374000 | -0.702322000 | -0.601000000 |
| B | -1.980916000 | 0.450522000 | -0.500079000 |
| C | 2.566158000 | -2.355076000 | -1.400508000 |
| H | 1.775108000 | -2.832425000 | -1.973389000 |
| C | -1.647586000 | -1.007048000 | -0.094472000 |
| C | -0.320872000 | -2.955418000 | 0.316764000 |
| H | 0.632458000 | -3.475971000 | 0.327252000 |
| C | -1.470873000 | -3.614361000 | 0.759274000 |
| H | -1.397739000 | -4.644400000 | 1.097566000 |
| C | -2.725436000 | -2.975870000 | 0.790223000 |
| H | -3.597396000 | -3.516800000 | 1.148963000 |
| C | -2.808107000 | -1.653570000 | 0.363597000 |
| C | -3.519783000 | 0.568439000 | -0.210927000 |
| C | -5.277804000 | -0.920130000 | 0.637237000 |
| H | -5.603978000 | -1.884879000 | 1.016109000 |
| C | -6.202104000 | 0.126946000 | 0.493804000 |
| H | -7.241660000 | -0.036167000 | 0.763836000 |
| C | -5.800671000 | 1.371047000 | 0.011205000 |
| H | -6.527649000 | 2.170985000 | -0.092362000 |
| C | -4.459936000 | 1.589005000 | -0.339871000 |
| H | -4.162291000 | 2.565131000 | -0.713350000 |
| C | 2.251044000 | -1.249791000 | -0.590024000 |
| C | 3.870987000 | -2.833959000 | -1.511309000 |
| H | 4.088223000 | -3.678690000 | -2.158440000 |
| C | 4.893157000 | -2.226227000 | -0.781019000 |
| H | 5.910717000 | -2.599805000 | -0.848323000 |
| C | 4.602043000 | -1.150225000 | 0.054661000 |
| H | 5.388818000 | -0.701359000 | 0.653990000 |
| C | 3.294658000 | -0.655223000 | 0.160979000 |
| C | 2.995760000 | 0.475725000 | 1.070381000 |
| C | 3.832597000 | 1.600491000 | 1.126262000 |
| H | 4.719880000 | 1.633395000 | 0.501082000 |
| C | 3.509927000 | 2.689126000 | 1.933403000 |
| H | 4.160864000 | 3.558405000 | 1.950486000 |
| C | 2.345465000 | 2.672947000 | 2.704597000 |
| H | 2.091331000 | 3.525104000 | 3.327550000 |
| C | 1.512706000 | 1.554535000 | 2.670215000 |
| H | 0.609977000 | 1.526456000 | 3.272979000 |
| C | 1.839635000 | 0.463671000 | 1.866741000 |
| H | 1.202928000 | -0.415932000 | 1.871280000 |
| C | -1.048929000 | 2.642142000 | -1.524275000 |
| C | -0.848248000 | 1.335848000 | -1.053910000 |
| C | 0.464462000 | 0.759697000 | -1.118493000 |
| C | 1.493612000 | 1.529437000 | -1.679939000 |
| C | 1.270347000 | 2.827180000 | -2.147204000 |
| C | -0.002240000 | 3.390331000 | -2.065500000 |
| H | -2.041755000 | 3.080081000 | -1.471761000 |
| H | 2.492107000 | 1.110572000 | -1.750498000 |
| H | 2.091320000 | 3.397710000 | -2.572605000 |
| H | -0.177958000 | 4.400576000 | -2.424157000 |

# Cartesian coordinates of optimized structures of 2a, 2b, 3a and 3b for the TD-DFT calculations, orbital energies, and transitions

## TD-DFT calculations 2a:

**Calculated absorption spectrum**

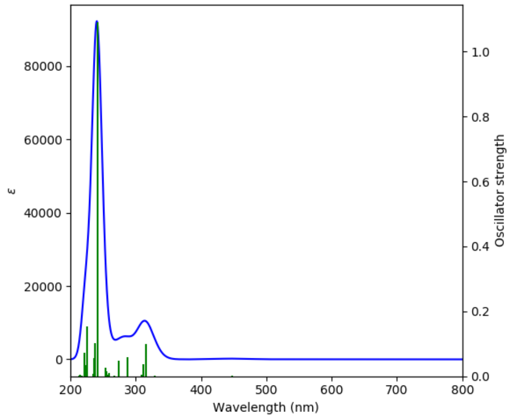


| Orbital | Energy [eV] | Symmetry |
|---|---|---|
| L+4 | -0.41 | A |
| L+3 | -0.09 | A |
| L+2 | -0.29 | A |
| L+1 | -1.72 | A |
| LUMO | -2.04 | A |
| HOMO | -7.53 | A |
| H-1 | -7.86 | A |
| H-2 | -8.57 | A |
| H-3 | -8.58 | A |
| H-4 | -8.94 | A |

**TD-DFT CAMB3LYP/6-31+G(d,p), gas phase**

Table S6: Lowest energy singlet electronic transition of **2a** (TD-DFT CAM-B3LYP/6-31+G(d,p), gas phase).

| State | E [eV] | λ [nm] | *f* | Symmetry | Major contributions | Λ |
|---|---|---|---|---|---|---|
| 1 | 2.77 | 448.08 | 0.0026 | Singlet-A | H-1→L+1 (22%), HOMO→LUMO (75%) | 0.60 |
| 2 | 2.88 | 430.84 | 0.0002 | Singlet-A | H-1→LUMO (51%), HOMO→L+1 (46%) | 0.60 |
| 3 | 3.77 | 329.00 | 0.0019 | Singlet-A | H-2→LUMO (12%), H-1→LUMO (35%), HOMO→L+1 (45%) | 0.60 |
| 4 | 3.93 | 315.36 | 0.1006 | Singlet-A | H-3→LUMO (14%), H-1→L+1 (54%), HOMO→LUMO (20%) | 0.60 |
| 5 | 3.97 | 312.18 | 0.0363 | Singlet-A | H-3→LUMO (54%), H-1→L+1 (21%) | 0.61 |
| 6 | 4.02 | 308.27 | 0.0042 | Singlet-A | H-3→L+1 (17%), H-2→LUMO (50%), H-1→LUMO (11%) | 0.62 |
| 7 | 4.31 | 287.89 | 0.0582 | Singlet-A | H-4→LUMO (50%), H-3→LUMO (11%), H-2→L+1 (22%) | 0.63 |
| 8 | 4.53 | 273.71 | 0.0468 | Singlet-A | H-5→LUMO (13%), H-4→L+1 (21%), H-3→L+1 (44%), H-2→LUMO (10%) | 0.62 |
| 9 | 4.65 | 266.87 | 0.0024 | Singlet-A | H-5→LUMO (44%), H-3→L+1 (25%), H-2→LUMO (10%) | 0.65 |
| 10 | 4.78 | 259.22 | 0.0104 | Singlet-A | H-5→L+1 (20%), H-4→LUMO (14%), H-3→LUMO (13%), H-2→L+1 (32%) | 0.63 |
| 11 | 4.85 | 255.87 | 0.0047 | Singlet-A | H-15→LUMO (17%), H-14→L+1 (17%), H-12→LUMO (22%), H-7→LUMO (11%) | 0.44 |
| 12 | 4.85 | 255.67 | 0.016 | Singlet-A | H-14→LUMO (22%), H-12→L+1 (11%), H-9→LUMO (10%), HOMO→L+2 (14%) | 0.50 |
| 13 | 4.90 | 253.02 | 0.0265 | Singlet-A | H-1→L+3 (10%), HOMO→L+2 (52%) | 0.68 |
| 14 | 5.15 | 240.97 | 1.0913 | Singlet-A | H-2→L+1 (18%), H-1→L+2 (26%), HOMO→L+3 (46%) | 0.71 |
| 15 | 5.21 | 237.82 | 0.1028 | Singlet-A | H-8→LUMO (16%), H-5→L+1 (13%), H-4→LUMO (20%) | 0.63 |
| 16 | 5.24 | 236.69 | 0.0552 | Singlet-A | H-6→LUMO (12%), H-5→LUMO (12%), H-4→L+1 (50%) | 0.68 |
| 17 | 5.29 | 234.50 | 0.0072 | Singlet-A | H-10→LUMO (30%) | 0.45 |
| 18 | 5.30 | 233.89 | 0.0003 | Singlet-A | H-16→LUMO (10%), H-11→LUMO (14%), H-10→L+1 (17%), H-9→LUMO (20%) | 0.44 |
| 19 | 5.49 | 225.84 | 0.1545 | Singlet-A | H-6→LUMO (59%) | 0.71 |
| 20 | 5.52 | 224.63 | 0.0341 | Singlet-A | H-8→LUMO (20%), H-6→L+1 (24%), H-5→L+1 (36%) | 0.68 |
| 21 | 5.53 | 224.13 | 0.0296 | Singlet-A | H-1→L+2 (44%), HOMO→L+3 (33%) | 0.71 |
| 22 | 5.59 | 221.98 | 0.0722 | Singlet-A | H-3→L+2 (18%), H-1→L+3 (39%), HOMO→L+2 (12%) | 0.68 |
| 23 | 5.71 | 216.98 | 0.0023 | Singlet-A | H-4→L+2 (12%), H-1→L+3 (13%), HOMO→L+9 (10%) | 0.64 |
| 24 | 5.78 | 214.34 | 0.0021 | Singlet-A | H-3→L+3 (17%), H-2→L+2 (16%), HOMO→L+6 (11%) | 0.59 |
| 25 | 5.79 | 214.05 | 0.0034 | Singlet-A | H-7→LUMO (71%) | 0.34 |

**Orbitals relevant to the $S_1 \leftarrow S_o$ and $S_2 \leftarrow S_o$ transitions**

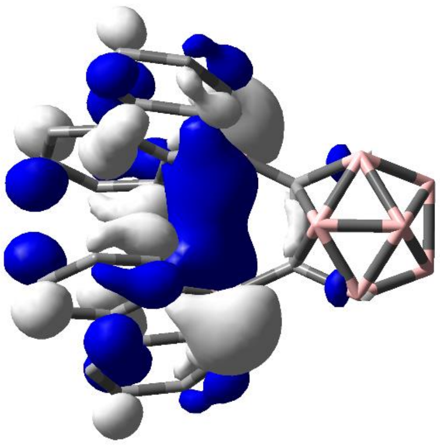

**LUMO:** -2.04 **eV**

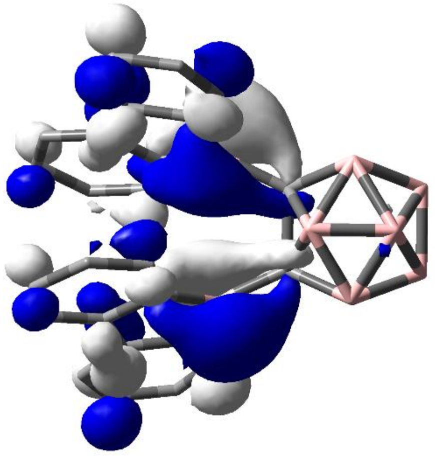

**LUMO+1:** -1.72 **eV**

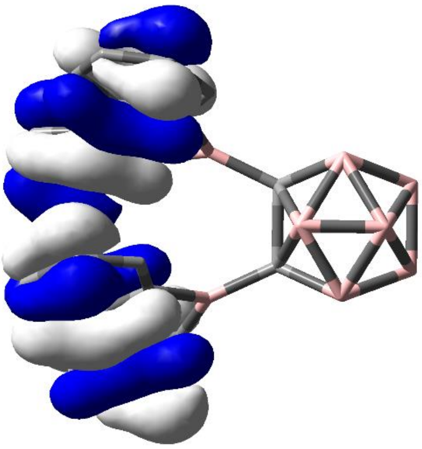

**HOMO:** -7.53 **eV**

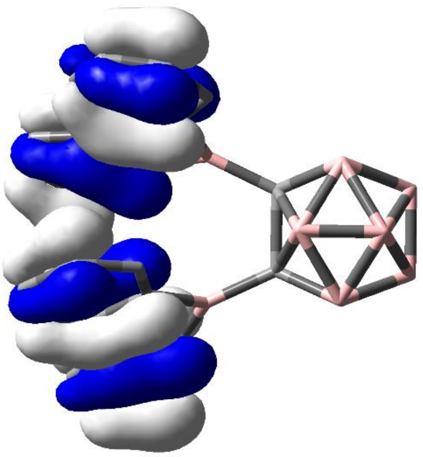

**HOMO-1:** -7.86 **eV**

Isovalue= 0.03

| | | | | |
|---|---|---|---|---|
| H | 1.67283600 | -1.59448300 | -1.73728900 | |
| B | 2.39100200 | 0.96788900 | 1.05534700 | |
| C | -1.86961000 | -1.27415200 | 1.45252200 | |
| B | 0.47775900 | 1.18248700 | -0.95459200 | |
| H | 1.67353600 | 1.59337700 | 1.73714400 | |
| H | 3.09116100 | -0.81109500 | 2.74327500 | |
| B | 0.47710700 | -1.18289000 | 0.95444800 | |
| C | 0.46092200 | 3.72384700 | 0.03663700 | |
| H | 1.51649800 | 3.76663000 | 0.27480700 | |
| C | 1.91379900 | -0.62775600 | 0.55460600 | |
| B | 3.26922600 | -1.65564400 | 0.30781500 | |
| H | 4.72219000 | 1.65807500 | 1.83973700 | |
| C | -0.08688500 | -2.59046100 | 0.57024500 | |
| B | 3.27007100 | 1.65374100 | -0.30788300 | |
| H | 5.68768900 | 1.13862100 | -1.03602400 | |
| H | 5.68707000 | -1.14173900 | 1.03606200 | |
| C | 1.91414200 | 0.62654000 | -0.55474200 | |
| B | 2.39056300 | -0.96935200 | -1.05545900 | |
| C | -0.35909300 | -4.81983800 | -0.34863200 | |
| H | 0.07130700 | -5.70032800 | -0.81544200 | |
| H | 4.72144000 | -1.66070900 | -1.83974000 | |
| B | 4.14515900 | 0.96649100 | 1.06989900 | |
| H | 3.09169400 | 0.80928200 | -2.74335500 | |
| B | 4.69642100 | -0.65964800 | 0.59946200 | |
| C | -1.72503400 | -4.77973000 | -0.06381300 | |
| H | -2.35331500 | -5.63012300 | -0.31252000 | |
| B | 3.24461500 | -0.47061000 | 1.62112100 | |
| C | -2.29640400 | -3.64805300 | 0.53775400 | |
| H | -3.36271200 | -3.62134200 | 0.74174300 | |
| B | 4.14472200 | -0.96883500 | -1.06992900 | |
| H | 3.14537200 | 2.80001300 | -0.55591500 | |
| C | -1.47930900 | -2.57037500 | 0.85220300 | |
| B | 3.24492400 | 0.46872000 | -1.62119400 | |
| H | 3.14395100 | -2.80184800 | 0.55585000 | |
| C | -0.73747400 | -0.41836000 | 1.58792600 | |
| B | 4.69677800 | 0.65702800 | -0.59946900 | |
| C | -3.13914000 | -0.86262500 | 1.82926600 | |
| H | -3.99803100 | -1.51529100 | 1.70471400 | |
| C | -3.30623400 | 0.42239000 | 2.37019000 | |
| H | -4.29712100 | 0.75693600 | 2.66335600 | |
| C | -2.21097700 | 1.26752900 | 2.53479000 | |
| H | -2.34637500 | 2.25962100 | 2.95313700 | |
| C | -0.93033400 | 0.84771500 | 2.14757200 | |
| H | -0.10179600 | 1.52895700 | 2.27820800 | |
| C | -0.08531200 | 2.59037300 | -0.57024900 | |
| C | -0.35598300 | 4.81983900 | 0.34885300 | |
| H | 0.07503600 | 5.70001900 | 0.81567800 | |
| C | -1.72199100 | 4.78060700 | 0.06423000 | |
| H | -2.34970300 | 5.63137400 | 0.31309600 | |
| C | -2.29415800 | 3.64933900 | -0.53735300 | |
| H | -3.36050900 | 3.62332100 | -0.74121100 | |
| C | -1.47778300 | 2.57117500 | -0.85200700 | |
| C | -1.86895700 | 1.27526900 | -1.45243700 | |
| C | -3.13878700 | 0.86460700 | -1.82911300 | |
| H | -3.99725100 | 1.51779700 | -1.70437000 | |
| C | -3.30675000 | -0.42020500 | -2.37024500 | |
| H | -4.29787800 | -0.75407500 | -2.66336900 | |
| C | -2.21203700 | -1.26598800 | -2.53513700 | |
| H | -2.34810100 | -2.25791400 | -2.95366000 | |
| C | -0.93108700 | -0.84704400 | -2.14799000 | |
| H | -0.10300700 | -1.52878200 | -2.27887500 | |
| C | -0.73736100 | 0.41879500 | -1.58810100 | |
| H | 1.51405500 | -3.76781100 | -0.27494300 | |
| C | 0.45854400 | -3.72433200 | -0.03662900 | |

## TD-DFT calculations 3a:

### Calculated absorption spectrum

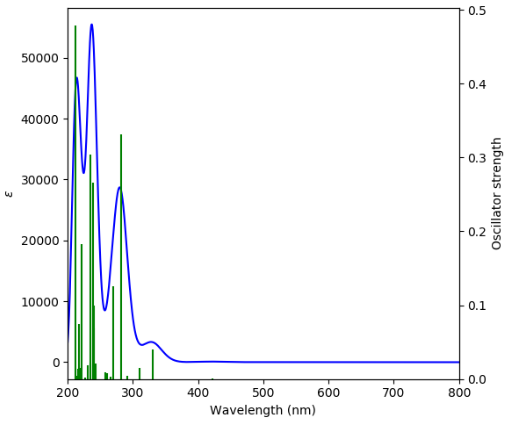


| Orbital | Energy [eV] | Symmetry |
|---|---|---|
| L+4 | 0.37 | A |
| L+3 | 0.15 | A |
| L+2 | -0.05 | A |
| L+1 | -1.19 | A |
| LUMO | -2.13 | A |
| HOMO | -7.83 | A |
| H-1 | -7.92 | A |
| H-2 | -8.52 | A |
| H-3 | -8.58 | A |
| H-4 | -8.87 | A |

### TD-DFT CAMB3LYP/6-31+G(d,p), gas phase

Table S7: Lowest energy singlet electronic transition of **3a** (TD-DFT CAM-B3LYP/6-31+G(d,p), gas phase).

| State | E [eV] | λ [nm] | *f* | Symmetry | Major contributions | Λ |
|---|---|---|---|---|---|---|
| 1 | 2.93 | 422.45 | 0.0014 | Singlet-A | H-1→LUMO (85%) | 0.55 |
| 2 | 3.74 | 331.07 | 0.0403 | Singlet-A | HOMO→LUMO (64%), HOMO→L+1 (13%) | 0.29 |
| 3 | 3.99 | 310.81 | 0.0153 | Singlet-A | H-4→LUMO (39%), H-1→L+1 (39%) | 0.65 |
| 4 | 4.25 | 291.41 | 0.004 | Singlet-A | H-2→LUMO (59%), H-2→L+1 (13%), HOMO→LUMO (12%) | 0.23 |
| 5 | 4.39 | 282.10 | 0.3311 | Singlet-A | H-4→LUMO (39%), H-1→L+1 (33%) | 0.65 |
| 6 | 4.61 | 269.21 | 0.1259 | Singlet-A | H-6→LUMO (63%), H-3→LUMO (11%) | 0.65 |
| 7 | 4.67 | 265.75 | 0.0038 | Singlet-A | H-15→LUMO (12%), H-11→LUMO (25%), H-7→LUMO (14%), HOMO→L+1 (17%) | 0.38 |
| 8 | 4.76 | 260.51 | 0.0084 | Singlet-A | H-3→LUMO (62%) | 0.28 |
| 9 | 4.81 | 257.75 | 0.0091 | Singlet-A | HOMO→L+1 (46%) | 0.36 |
| 10 | 5.09 | 243.52 | 0.0208 | Singlet-A | H-5→LUMO (16%), H-2→L+2 (11%), HOMO→L+5 (18%) | 0.47 |
| 11 | 5.12 | 242.11 | 0.0008 | Singlet-A | H-14→LUMO (21%), H-13→LUMO (29%) | 0.42 |
| 12 | 5.15 | 240.63 | 0.0688 | Singlet-A | H-5→LUMO (13%), H-2→L+2 (10%) | 0.46 |
| 13 | 5.17 | 239.65 | 0.0991 | Singlet-A | H-6→L+1 (14%), H-4→L+1 (13%) | 0.56 |
| 14 | 5.20 | 238.29 | 0.2662 | Singlet-A | H-5→LUMO (10%), HOMO→L+2 (57%) | 0.68 |
| 15 | 5.28 | 235.03 | 0.0206 | Singlet-A | H-9→LUMO (15%), H-8→LUMO (22%) | 0.50 |
| 16 | 5.28 | 234.80 | 0.3035 | Singlet-A | H-6→LUMO (12%), H-4→L+1 (38%) | 0.61 |
| 17 | 5.37 | 230.98 | 0.0188 | Singlet-A | H-9→LUMO (13%), H-5→LUMO (28%), H-3→L+2 (11%) | 0.39 |
| 18 | 5.39 | 230.19 | 0.0023 | Singlet-A | H-2→LUMO (13%), H-2→L+1 (44%) | 0.29 |
| 19 | 5.46 | 227.26 | 0.0021 | Singlet-A | H-11→LUMO (24%), H-7→LUMO (50%) | 0.35 |
| 20 | 5.62 | 220.79 | 0.1826 | Singlet-A | H-10→LUMO (16%), H-9→LUMO (15%), H-1→L+3 (26%) | 0.56 |
| 21 | 5.67 | 218.63 | 0.0146 | Singlet-A | H-15→LUMO (37%), H-9→LUMO (13%) | 0.48 |
| 22 | 5.69 | 217.86 | 0.011 | Singlet-A | H-10→LUMO (46%), H-8→LUMO (15%), H-3→L+1 (14%) | 0.50 |
| 23 | 5.70 | 217.35 | 0.0744 | Singlet-A | H-10→LUMO (20%), H-3→L+1 (49%) | 0.36 |
| 24 | 5.75 | 215.55 | 0.0142 | Singlet-A | H-12→LUMO (79%) | 0.42 |
| 25 | 5.81 | 213.56 | 0.0047 | Singlet-A | H-14→LUMO (23%), H-13→LUMO (38%) | 0.38 |

**Orbitals relevant to the $S_1 \leftarrow S_o$ and $S_2 \leftarrow S_o$ transitions**

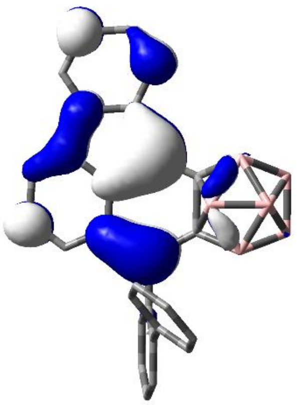

**LUMO:** -2.13 **eV**

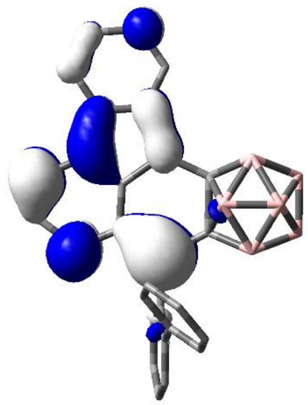

**LUMO+1:** -1.19 **eV**

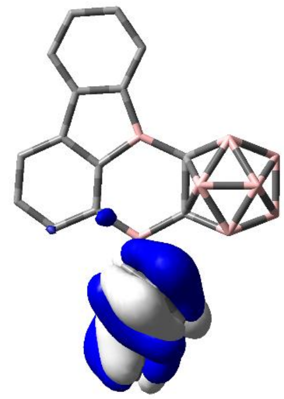

**HOMO:** -7.83 **eV**

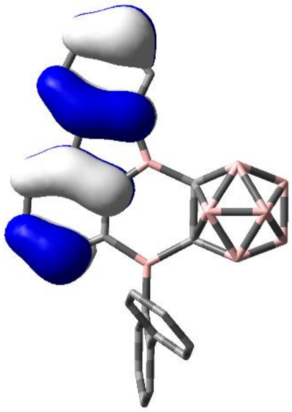

**HOMO-1:** -7.92 **eV**

Isovalue= 0.03

| | | | | |
|---|---|---|---|---|
| H | -0.11437800 | 1.00249100 | 1.90953000 | |
| B | -0.66272300 | 1.70243700 | -1.83068800 | |
| C | -3.94761000 | -1.40795800 | 0.21882800 | |
| C | -0.31763500 | -1.55962400 | -0.55747600 | |
| B | 0.77129200 | -0.44580200 | -0.69711300 | |
| H | -0.99693100 | 0.95728500 | -2.68533400 | |
| H | -2.87763800 | 2.94547900 | -1.48300900 | |
| B | -2.22158200 | 0.23059900 | -0.07412900 | |
| C | 2.36030800 | -1.11549000 | -2.52665200 | |
| H | 1.47682400 | -1.10399400 | -3.16134200 | |
| C | -1.30477100 | 1.48123600 | -0.24723600 | |
| B | -1.43151900 | 2.91268700 | 0.66071900 | |
| H | -0.38941200 | 3.99032900 | -2.98385800 | |
| C | -1.63424000 | -1.19017600 | -0.25562200 | |
| B | 1.00646300 | 2.29015700 | -1.60384400 | |
| H | 1.75081900 | 4.65951800 | -0.87036500 | |
| H | -1.18719000 | 5.30375200 | -0.31109700 | |
| C | 0.34311000 | 1.09949000 | -0.55994900 | |
| B | -0.12464600 | 1.72832300 | 0.97855400 | |
| C | -0.05962400 | -2.94633100 | -0.69729900 | |
| H | 0.94301300 | -3.29059800 | -0.93364200 | |
| H | 0.55371400 | 4.04042900 | 1.89621900 | |
| B | -0.31197800 | 3.43530900 | -1.93861800 | |
| H | 2.38989500 | 1.97381700 | 0.55092900 | |
| B | -0.77361700 | 4.19382500 | -0.37950100 | |
| C | -1.08415600 | -3.88041500 | -0.53543800 | |
| H | -0.86341700 | -4.93831500 | -0.64812700 | |
| B | -1.76706600 | 2.89584600 | -1.07576900 | |
| C | -2.40167900 | -3.48515800 | -0.22775500 | |
| H | -3.17552100 | -4.23910700 | -0.10877100 | |
| B | 0.23788500 | 3.46281900 | 0.90991500 | |
| H | 1.82589900 | 1.93845900 | -2.37892000 | |
| C | -2.67523700 | -2.12821700 | -0.08796600 | |
| B | 1.33994100 | 2.30494500 | 0.12986400 | |
| H | -2.31116500 | 2.97699800 | 1.45061100 | |
| C | -3.73055000 | 0.00507400 | 0.23887600 | |
| B | 0.92918500 | 3.81988200 | -0.70463000 | |
| C | -5.20787700 | -1.93710000 | 0.46170900 | |
| H | -5.38151100 | -3.00971200 | 0.44737900 | |
| C | -6.27409500 | -1.05939500 | 0.73076200 | |
| H | -7.26338500 | -1.46553000 | 0.92242500 | |
| C | -6.07860400 | 0.32204200 | 0.75395400 | |
| H | -6.91402000 | 0.98335100 | 0.96296500 | |
| C | -4.80371200 | 0.85646300 | 0.50677800 | |
| H | -4.65702700 | 1.93255400 | 0.52511000 | |
| C | 2.23206500 | -0.81651300 | -1.15402000 | |
| C | 3.59598900 | -1.40005300 | -3.10850100 | |
| H | 3.65963900 | -1.60986700 | -4.17223600 | |
| C | 4.74161600 | -1.41624500 | -2.31134800 | |
| H | 5.71043700 | -1.64713600 | -2.74476700 | |
| C | 4.63442400 | -1.15611500 | -0.94639400 | |
| H | 5.52058800 | -1.21100600 | -0.32090200 | |
| C | 3.39702800 | -0.85659300 | -0.34784400 | |
| C | 3.35372900 | -0.63108600 | 1.12467200 | |
| C | 4.32654900 | 0.16512400 | 1.75712700 | |
| H | 5.08720700 | 0.65507300 | 1.15651200 | |
| C | 4.30370600 | 0.36637000 | 3.13688100 | |
| H | 5.05664700 | 0.99776400 | 3.59997700 | |
| C | 3.30870600 | -0.22738700 | 3.92031500 | |
| H | 3.28978100 | -0.06838400 | 4.99443600 | |
| C | 2.34118300 | -1.02648500 | 3.30907500 | |
| H | 1.57023400 | -1.50417700 | 3.90707100 | |
| C | 2.36604100 | -1.22850400 | 1.92636000 | |
| H | 1.63072900 | -1.88633000 | 1.47437100 | |

## TD-DFT calculations 2b:

### Calculated absorption spectrum

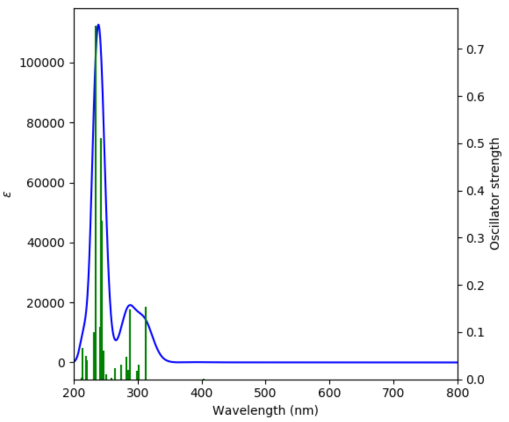


| Orbital | Energy [eV] | Symmetry |
|---|---|---|
| L+4 | 0.67 | A |
| L+3 | 0.18 | A |
| L+2 | 0.06 | A |
| L+1 | -1.10 | A |
| LUMO | -1.54 | A |
| HOMO | -7.32 | A |
| H-1 | -7.37 | A |
| H-2 | -8.11 | A |
| H-3 | -8.25 | A |
| H-4 | -8.29 | A |

### TD-DFT CAMB3LYP/6-31+G(d, p), gas phase

Table S8: Lowest energy singlet electronic transition of **2b** (TD-DFT CAM-B3LYP/6-31+G(d,p), gas phase).

| State | E [eV] | λ [nm] | *f* | Symmetry | Major contributions | Λ |
|---|---|---|---|---|---|---|
| 1 | 3.07 | 403.46 | 0.0016 | Singlet-A | H-1→L+1 (30%), HOMO→LUMO (66%) | 0.61 |
| 2 | 3.10 | 400.57 | 0.0001 | Singlet-A | H-1→LUMO (62%), HOMO→L+1 (35%) | 0.61 |
| 3 | 3.97 | 312.68 | 0.1529 | Singlet-A | H-2→LUMO (83%) | 0.61 |
| 4 | 4.11 | 301.61 | 0.0315 | Singlet-A | H-4→L+1 (10%), H-3→LUMO (46%), H-1→L+1 (14%) | 0.66 |
| 5 | 4.15 | 298.63 | 0.0173 | Singlet-A | H-4→LUMO (45%), H-3→L+1 (17%), HOMO→L+2 (11%) | 0.68 |
| 6 | 4.31 | 287.37 | 0.1489 | Singlet-A | H-5→LUMO (67%) | 0.56 |
| 7 | 4.34 | 285.63 | 0.0197 | Singlet-A | H-1→LUMO (34%), HOMO→L+1 (52%) | 0.62 |
| 8 | 4.39 | 282.70 | 0.0481 | Singlet-A | H-1→L+1 (51%), HOMO→LUMO (29%) | 0.63 |
| 9 | 4.53 | 273.78 | 0.0319 | Singlet-A | H-2→L+1 (70%) | 0.56 |
| 10 | 4.68 | 265.18 | 0.0236 | Singlet-A | H-7→L+1 (12%), H-6→LUMO (50%) | 0.72 |
| 11 | 4.79 | 258.88 | 0.0038 | Singlet-A | H-7→LUMO (41%), H-6→L+1 (25%) | 0.75 |
| 12 | 4.95 | 250.37 | 0.0103 | Singlet-A | H-10→LUMO (19%), H-5→L+1 (42%), H-4→L+1 (17%) | 0.56 |
| 13 | 5.03 | 246.43 | 0.0616 | Singlet-A | H-4→LUMO (28%), H-1→L+3 (21%), HOMO→L+2 (28%) | 0.69 |
| 14 | 5.08 | 243.91 | 0.3361 | Singlet-A | H-10→LUMO (39%), H-3→LUMO (12%), H-1→L+2 (12%), HOMO→L+3 (11%) | 0.60 |
| 15 | 5.10 | 242.93 | 0.5115 | Singlet-A | H-10→LUMO (17%), H-5→L+1 (15%), H-3→LUMO (17%), H-1→L+2 (17%), HOMO→L+3 (15%) | 0.64 |
| 16 | 5.14 | 241.28 | 0.1113 | Singlet-A | H-3→L+1 (46%), HOMO→L+2 (15%) | 0.69 |
| 17 | 5.29 | 234.49 | 0.0401 | Singlet-A | H-12→LUMO (36%), H-11→L+1 (24%), H-4→L+1 (11%) | 0.60 |
| 18 | 5.30 | 233.92 | 0.0296 | Singlet-A | H-13→LUMO (20%), H-12→L+1 (13%), H-11→LUMO (29%), H-10→L+1 (23%) | 0.53 |
| 19 | 5.30 | 233.91 | 0.7495 | Singlet-A | H-5→L+1 (20%), H-4→L+1 (31%) | 0.66 |
| 20 | 5.36 | 231.29 | 0.0998 | Singlet-A | H-13→LUMO (19%), H-11→LUMO (14%), H-10→L+1 (20%), H-3→L+1 (13%) | 0.58 |
| 21 | 5.62 | 220.63 | 0.0395 | Singlet-A | H-12→LUMO (11%), H-9→LUMO (26%), H-8→L+1 (11%), H-6→LUMO (19%) | 0.69 |
| 22 | 5.64 | 219.69 | 0.0496 | Singlet-A | H-11→LUMO (11%), H-9→L+1 (10%), H-8→LUMO (35%) | 0.70 |
| 23 | 5.78 | 214.32 | 0.0665 | Singlet-A | H-6→L+1 (13%), H-5→LUMO (11%), H-2→L+5 (22%) | 0.64 |
| 24 | 5.80 | 213.68 | 0.0049 | Singlet-A | H-7→LUMO (13%), H-4→L+3 (13%), H-3→L+2 (21%) | 0.69 |
| 25 | 5.82 | 212.99 | 0.0029 | Singlet-A | H-9→LUMO (23%), H-7→L+1 (11%), H-6→LUMO (17%) | 0.73 |

**Orbitals relevant to the $S_1 \leftarrow S_0$ and $S_2 \leftarrow S_0$ transitions**

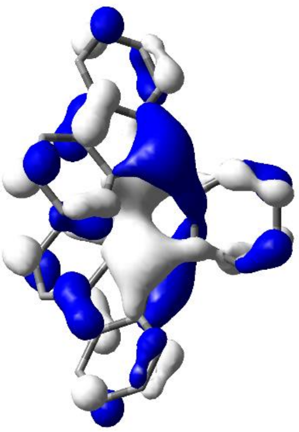

**LUMO:** -1.54 **eV**

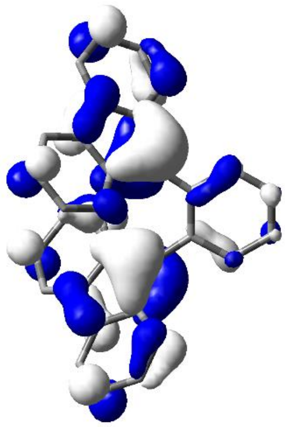

**LUMO+1:** -1.10 **eV**

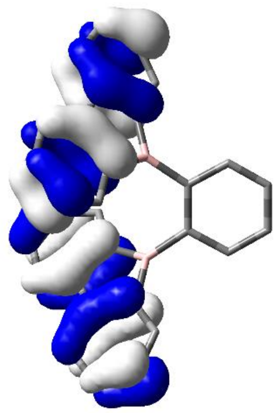

**HOMO:** -7.32 **eV**

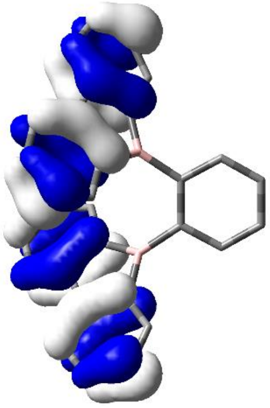

**HOMO-1:** -7.37 **eV**

Isovalue= 0.03

| | | | | |
|---|---|---|---|---|
| B | 1.54656300 | 0.77025200 | -0.41969800 | |
| B | -1.54649400 | 0.77027300 | 0.41962900 | |
| C | -1.22882400 | -0.41785100 | 1.39776300 | |
| C | -0.15304600 | -0.69239100 | 2.24415100 | |
| H | 0.69777000 | -0.01752600 | 2.28951800 | |
| C | -0.16236500 | -1.84501700 | 3.04618100 | |
| H | 0.67868800 | -2.06119900 | 3.69850700 | |
| C | -1.25508500 | -2.71284300 | 3.00801500 | |
| H | -1.25856500 | -3.60242600 | 3.63212200 | |
| C | -2.35713800 | -2.44647100 | 2.17736100 | |
| H | -3.20419500 | -3.12743600 | 2.16910000 | |
| C | -2.34039000 | -1.30543500 | 1.38302600 | |
| C | -3.38749800 | -0.80584900 | 0.45080200 | |
| C | -4.61142600 | -1.38151300 | 0.12620200 | |
| H | -4.92710300 | -2.32250700 | 0.56901800 | |
| C | -5.44732500 | -0.72855500 | -0.79564200 | |
| H | -6.40550100 | -1.17066700 | -1.05515500 | |
| C | -5.05932000 | 0.47661400 | -1.38447400 | |
| H | -5.71512600 | 0.96594600 | -2.09892000 | |
| C | -3.82090500 | 1.05130700 | -1.05464000 | |
| H | -3.52004100 | 1.98444400 | -1.52519700 | |
| C | -2.97966100 | 0.42513200 | -0.13307000 | |
| C | -0.70297100 | 2.04896900 | 0.13047400 | |
| C | -1.36575600 | 3.28918200 | 0.24157500 | |
| H | -2.43360800 | 3.30368800 | 0.44478800 | |
| C | -0.68642300 | 4.50447900 | 0.12372100 | |
| H | -1.22717400 | 5.44185300 | 0.22205500 | |
| C | 0.68647100 | 4.50451100 | -0.12366600 | |
| H | 1.22719200 | 5.44191100 | -0.22192300 | |
| C | 1.36584400 | 3.28924800 | -0.24161700 | |
| H | 2.43369300 | 3.30380400 | -0.44485100 | |
| C | 0.70311400 | 2.04900900 | -0.13060800 | |
| C | 1.22879800 | -0.41793300 | -1.39772400 | |
| C | 0.15298000 | -0.69248400 | -2.24405800 | |
| H | -0.69779500 | -0.01757000 | -2.28946400 | |
| C | 0.16219900 | -1.84518900 | -3.04597500 | |
| H | -0.67888700 | -2.06138000 | -3.69825500 | |
| C | 1.25486400 | -2.71308100 | -3.00775600 | |
| H | 1.25826700 | -3.60272700 | -3.63177500 | |
| C | 2.35696100 | -2.44669800 | -2.17716500 | |
| H | 3.20397400 | -3.12771800 | -2.16886300 | |
| C | 2.34030800 | -1.30558700 | -1.38293700 | |
| C | 3.38748500 | -0.80597000 | -0.45080500 | |
| C | 4.61140900 | -1.38165300 | -0.12623000 | |
| H | 4.92701900 | -2.32270400 | -0.56897200 | |
| C | 5.44739400 | -0.72863800 | 0.79549700 | |
| H | 6.40556600 | -1.17076700 | 1.05499700 | |
| C | 5.05947800 | 0.47660600 | 1.38423300 | |
| H | 5.71534900 | 0.96598300 | 2.09858900 | |
| C | 3.82106600 | 1.05132000 | 1.05442100 | |
| H | 3.52026600 | 1.98450800 | 1.52491500 | |
| C | 2.97973900 | 0.42509200 | 0.13296200 | |

## TD-DFT calculations 3b:

### Calculated absorption spectrum

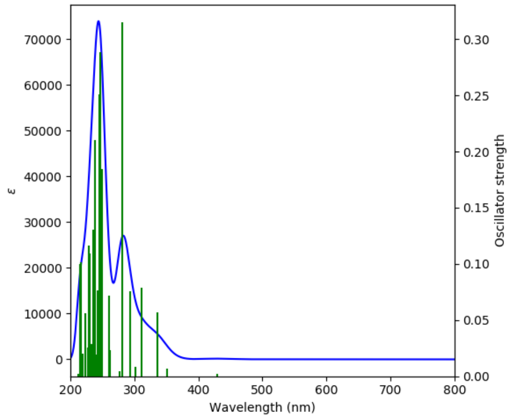


| Orbital | Energy [eV] | Symmetry |
|---|---|---|
| L+4 | 0.63 | A |
| L+3 | 0.37 | A |
| L+2 | 0.16 | A |
| L+1 | -0.67 | A |
| LUMO | -1.81 | A |
| HOMO | -7.38 | A |
| H-1 | -7.62 | A |
| H-2 | -8.11 | A |
| H-3 | -8.24 | A |
| H-4 | -8.32 | A |

### TD-DFT CAMB3LYP/6-31+G(d,p), gas phase

Table S9: Lowest energy singlet electronic transition of **3b** (TD-DFT CAM-B3LYP/6-31+G(d,p), gas phase).

| State | E [eV] | λ [nm] | *f* | Symmetry | Major contributions | Λ |
|---|---|---|---|---|---|---|
| 1 | 2.89 | 429.67 | 0.0022 | Singlet-A | HOMO→LUMO (91%) | 0.51 |
| 2 | 3.54 | 350.44 | 0.0066 | Singlet-A | H-2→LUMO (89%) | 0.60 |
| 3 | 3.70 | 335.18 | 0.0571 | Singlet-A | H-3→LUMO (16%), H-1→LUMO (62%) | 0.34 |
| 4 | 3.98 | 311.74 | 0.0791 | Singlet-A | H-4→LUMO (56%), HOMO→L+1 (23%) | 0.60 |
| 5 | 4.11 | 301.52 | 0.008 | Singlet-A | H-9→LUMO (28%), H-3→LUMO (26%), H-1→LUMO (14%) | 0.50 |
| 6 | 4.23 | 293.04 | 0.0753 | Singlet-A | H-6→LUMO (24%), H-3→LUMO (13%), HOMO→L+1 (23%) | 0.54 |
| 7 | 4.41 | 281.31 | 0.3153 | Singlet-A | H-6→LUMO (25%), H-4→LUMO (17%), HOMO→L+1 (24%) | 0.62 |
| 8 | 4.49 | 276.13 | 0.0047 | Singlet-A | H-9→LUMO (29%), H-3→LUMO (22%) | 0.53 |
| 9 | 4.74 | 261.55 | 0.0231 | Singlet-A | H-11→LUMO (46%), H-5→LUMO (27%) | 0.47 |
| 10 | 4.77 | 260.19 | 0.0716 | Singlet-A | H-11→LUMO (30%), H-5→LUMO (41%) | 0.43 |
| 11 | 4.96 | 249.72 | 0.1841 | Singlet-A | H-8→LUMO (16%), H-2→L+1 (10%), H-1→L+1 (18%), HOMO→L+1 (11%) | 0.55 |
| 12 | 5.03 | 246.61 | 0.2885 | Singlet-A | H-2→L+1 (45%), H-1→L+1 (23%) | 0.50 |
| 13 | 5.05 | 245.74 | 0.2508 | Singlet-A | H-12→LUMO (10%), H-8→LUMO (14%), H-1→L+1 (17%) | 0.55 |
| 14 | 5.10 | 243.01 | 0.0764 | Singlet-A | H-12→LUMO (20%), H-7→LUMO (12%), H-3→L+2 (10%), H-1→L+5 (12%) | 0.53 |
| 15 | 5.16 | 240.19 | 0.019 | Singlet-A | H-12→LUMO (37%), H-1→L+5 (10%) | 0.51 |
| 16 | 5.21 | 238.12 | 0.2103 | Singlet-A | H-1→L+2 (48%) | 0.67 |
| 17 | 5.26 | 235.51 | 0.1306 | Singlet-A | H-3→L+2 (12%), H-1→L+2 (26%) | 0.760 |
| 18 | 5.31 | 233.29 | 0.0288 | Singlet-A | H-10→LUMO (23%), H-9→LUMO (14%), H-8→LUMO (29%) | 0.67 |
| 19 | 5.38 | 230.27 | 0.1095 | Singlet-A | H-7→LUMO (16%), H-6→LUMO (13%), H-4→L+1 (17%) | 0.56 |
| 20 | 5.43 | 228.32 | 0.1167 | Singlet-A | H-9→L+1 (12%), H-7→LUMO (27%), H-3→L+1 (12%) | 0.47 |
| 21 | 5.46 | 227.00 | 0.0253 | Singlet-A | H-13→LUMO (16%), H-9→L+1 (12%), H-1→L+1 (11%) | 0.51 |
| 22 | 5.55 | 223.40 | 0.056 | Singlet-A | H-4→L+1 (36%), HOMO→L+3 (23%) | 0.58 |
| 23 | 5.66 | 219.15 | 0.0198 | Singlet-A | H-13→LUMO (36%), H-3→L+1 (36%) | 0.42 |
| 24 | 5.73 | 216.23 | 0.1017 | Singlet-A | H-6→L+1 (40%), H-5→L+1 (12%) | 0.59 |
| 25 | 5.77 | 214.90 | 0.0996 | Singlet-A | H-10→LUMO (11%), H-8→L+1 (10%), HOMO→L+3 (11%), HOMO→L+12 (14%) | 0.64 |

**Orbitals relevant to the $S_1 \leftarrow S_o$ and $S_2 \leftarrow S_o$ transitions**

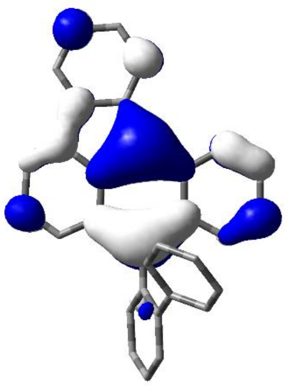

**LUMO:** -1.81 **eV**

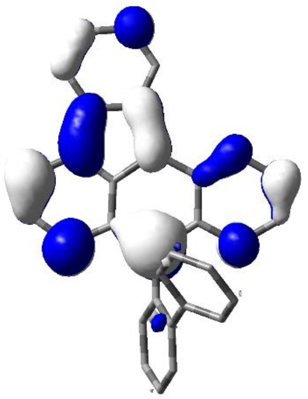

**LUMO+1:** -0.67 **eV**

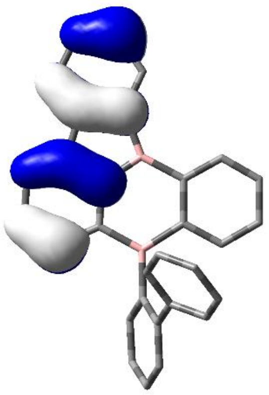

**HOMO:** -7.38 **eV**

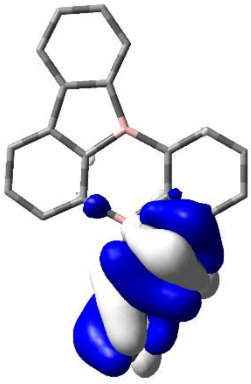

**HOMO-1:** -7.62 **eV**

Isovalue= 0.03

| | | | | |
|---|---|---|---|---|
| C | 4.01006900 | 0.68943100 | 0.36383000 | |
| C | 0.43005200 | 1.52432700 | -0.11335700 | |
| B | -0.72385700 | 0.59121100 | -0.62791500 | |
| B | 2.07660800 | -0.50699000 | -0.46751900 | |
| C | -2.42304500 | 2.16556000 | -1.68631600 | |
| H | -1.58168500 | 2.55512800 | -2.25524300 | |
| C | 1.69948800 | 0.94017200 | -0.05059200 | |
| C | 0.32325100 | 2.86154600 | 0.33657100 | |
| H | -0.63747700 | 3.36955700 | 0.32423400 | |
| C | 1.45075300 | 3.54495100 | 0.80503200 | |
| H | 1.34787400 | 4.57312300 | 1.14219000 | |
| C | 2.71901500 | 2.93337800 | 0.85787100 | |
| H | 3.57148600 | 3.49494100 | 1.23237100 | |
| C | 2.83832900 | 1.61316500 | 0.42747400 | |
| C | 3.61951900 | -0.58611600 | -0.15761500 | |
| C | 5.32570300 | 0.94015000 | 0.73557400 | |
| H | 5.62167500 | 1.90858600 | 1.13050600 | |
| C | 6.28265100 | -0.07982200 | 0.59657600 | |
| H | 7.31294900 | 0.10909800 | 0.88568100 | |
| C | 5.92283400 | -1.33020600 | 0.09283600 | |
| H | 6.67255900 | -2.10962700 | -0.00831600 | |
| C | 4.59264400 | -1.58016200 | -0.28289800 | |
| H | 4.33068700 | -2.56049800 | -0.67231200 | |
| C | -2.19730000 | 1.14785000 | -0.73612400 | |
| C | -3.69872200 | 2.67110800 | -1.94304400 | |
| H | -3.83693100 | 3.44418000 | -2.69375600 | |
| C | -4.78977800 | 2.18316900 | -1.22083000 | |
| H | -5.78660100 | 2.57807500 | -1.39582500 | |
| C | -4.59084600 | 1.19922100 | -0.25299100 | |
| H | -5.43396400 | 0.85026700 | 0.33672200 | |
| C | -3.31256700 | 0.67067100 | 0.00095000 | |
| C | -3.15444000 | -0.35827900 | 1.06633300 | |
| C | -4.06195200 | -1.42723200 | 1.17941700 | |
| H | -4.87238300 | -1.51724400 | 0.46154200 | |
| C | -3.91702800 | -2.39027000 | 2.17887500 | |
| H | -4.62354200 | -3.21351900 | 2.23877300 | |
| C | -2.86164800 | -2.30496500 | 3.09225100 | |
| H | -2.74799700 | -3.05520400 | 3.86932900 | |
| C | -1.95637400 | -1.24578000 | 2.99744000 | |
| H | -1.13822800 | -1.16167600 | 3.70713800 | |
| C | -2.10379200 | -0.28187900 | 1.99735400 | |
| H | -1.41305600 | 0.55524000 | 1.95850200 | |
| C | 1.20746100 | -2.71651800 | -1.52831800 | |
| C | 0.96756400 | -1.41801900 | -1.04798300 | |
| C | -0.36214800 | -0.87388400 | -1.12798300 | |
| C | -1.36286800 | -1.67178400 | -1.70884200 | |
| C | -1.09920700 | -2.96147600 | -2.18536800 | |
| C | 0.18806400 | -3.48987800 | -2.09209700 | |
| H | 2.21008000 | -3.13093600 | -1.46776500 | |
| H | -2.37211100 | -1.28086800 | -1.79502800 | |
| H | -1.89946500 | -3.54926900 | -2.62715700 | |
| H | 0.39830400 | -4.49103400 | -2.45857400 | |

## CAM-B3LYP-Hexane

### TD-DFT calculations 3a:

**Calculated absorption spectrum**

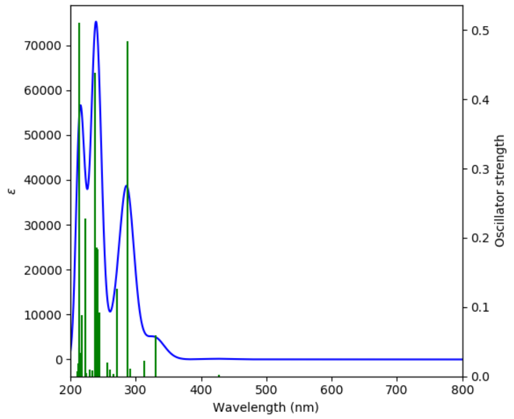


| Orbital | Energy [eV] | Symmetry |
|---|---|---|
| L+4 | 0.42 | A |
| L+3 | 0.20 | A |
| L+2 | -0.04 | A |
| L+1 | -1.15 | A |
| LUMO | -2.10 | A |
| HOMO | -7.81 | A |
| H-1 | -7.87 | A |
| H-2 | -8.51 | A |
| H-3 | -8.57 | A |
| H-4 | -8.81 | A |

**TD-DFT CAMB3LYP/6-31+G(d, p), n-Hexane**

Table S10: Lowest energy singlet electronic transition of **3a** (TD-DFT CAM-B3LYP/6-31+G(d, p), n-hexane).

| State | E [eV] | λ [nm] | *f* | Symmetry | Major contributions | Λ |
|---|---|---|---|---|---|---|
| 1 | 2.90 | 427.41 | 0.0021 | Singlet-A | H-1→LUMO (68%), HOMO→LUMO (25%) | 0.51 |
| 2 | 3.75 | 330.81 | 0.059 | Singlet-A | H-1→LUMO (16%), HOMO→LUMO (52%), HOMO→L+1 (12%) | 0.41 |
| 3 | 3.97 | 312.24 | 0.0231 | Singlet-A | H-4→LUMO (40%), H-1→L+1 (33%) | 0.62 |
| 4 | 4.26 | 291.15 | 0.0106 | Singlet-A | H-2→LUMO (59%), H-2→L+1 (13%), HOMO→LUMO (10%) | 0.25 |
| 5 | 4.32 | 286.97 | 0.4836 | Singlet-A | H-4→LUMO (39%), H-1→L+1 (30%), HOMO→L+1 (10%) | 0.63 |
| 6 | 4.57 | 271.09 | 0.1269 | Singlet-A | H-6→LUMO (69%) | 0.68 |
| 7 | 4.67 | 265.29 | 0.0036 | Singlet-A | H-15→LUMO (10%), H-11→LUMO (24%), H-8→LUMO (24%), HOMO→L+1 (12%) | 0.42 |
| 8 | 4.77 | 259.90 | 0.0097 | Singlet-A | H-3→LUMO (66%) | 0.29 |
| 9 | 4.83 | 256.73 | 0.02 | Singlet-A | H-1→L+1 (10%), HOMO→L+1 (41%) | 0.47 |
| 10 | 5.09 | 243.65 | 0.0928 | Singlet-A | H-5→LUMO (11%), H-2→L+2 (10%), HOMO→L+2 (19%), HOMO→L+5 (12%) | 0.53 |
| 11 | 5.12 | 242.17 | 0.1836 | Singlet-A | H-7→LUMO (29%), H-6→L+1 (21%), H-4→L+1 (14%) | 0.65 |
| 12 | 5.13 | 241.49 | 0.0104 | Singlet-A | H-14→LUMO (17%), H-13→LUMO (34%) | 0.42 |
| 13 | 5.16 | 240.37 | 0.1856 | Singlet-A | H-2→L+2 (11%), HOMO→L+2 (20%) | 0.61 |
| 14 | 5.17 | 239.72 | 0.1557 | Singlet-A | H-5→LUMO (28%), HOMO→L+2 (21%) | 0.47 |
| 15 | 5.23 | 236.85 | 0.4388 | Singlet-A | H-7→LUMO (13%), H-6→LUMO (11%), H-4→L+1 (35%) | 0.62 |
| 16 | 5.31 | 233.54 | 0.0083 | Singlet-A | H-9→LUMO (24%), H-5→LUMO (19%) | 0.34 |
| 17 | 5.39 | 230.01 | 0.0096 | Singlet-A | H-9→LUMO (16%), H-5→LUMO (22%), H-3→L+2 (11%) | 0.37 |
| 18 | 5.41 | 228.97 | 0.0015 | Singlet-A | H-9→LUMO (19%), H-2→LUMO (10%), H-2→L+1 (35%) | 0.30 |
| 19 | 5.54 | 223.80 | 0.005 | Singlet-A | H-11→LUMO (33%), H-8→LUMO (34%) | 0.41 |
| 20 | 5.56 | 222.95 | 0.2281 | Singlet-A | H-7→LUMO (39%), H-1→L+3 (22%) | 0.65 |
| 21 | 5.71 | 217.06 | 0.0881 | Singlet-A | H-3→L+1 (58%) | 0.31 |
| 22 | 5.74 | 216.09 | 0.0345 | Singlet-A | H-15→LUMO (34%), H-9→LUMO (24%) | 0.43 |
| 23 | 5.80 | 213.86 | 0.0415 | Singlet-A | H-10→LUMO (85%) | 0.40 |
| 24 | 5.80 | 213.66 | 0.5111 | Singlet-A | H-6→L+1 (36%), H-4→L+1 (10%) | 0.60 |
| 25 | 5.85 | 212.11 | 0.019 | Singlet-A | H-12→LUMO (82%) | 0.33 |

**Orbitals relevant to the $S_1 \leftarrow S_o$ and $S_2 \leftarrow S_o$ transitions**

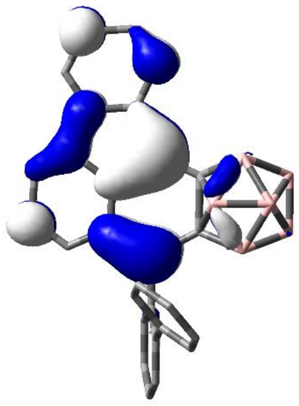

**LUMO:** -2.10 **eV**

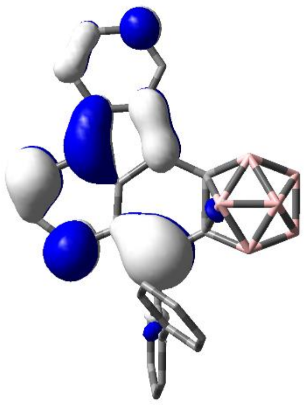

**LUMO+1:** -1.15 **eV**

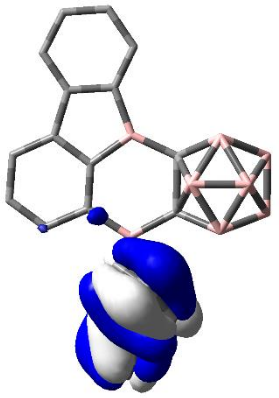

**HOMO:** -7.81 **eV**

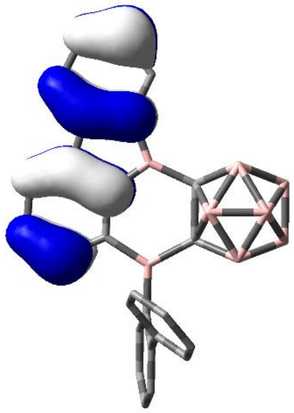

**HOMO-1:** -7.87 **eV**

Isovalue= 0.03

| | | | |
|---|---|---|---|
| H | -0.11437800 | 1.00249100 | 1.90953000 |
| B | -0.66272300 | 1.70243700 | -1.83068800 |
| C | -3.94761000 | -1.40795800 | 0.21882800 |
| C | -0.31763500 | -1.55962400 | -0.55747600 |
| B | 0.77129200 | -0.44580200 | -0.69711300 |
| H | -0.99693100 | 0.95728500 | -2.68533400 |
| H | -2.87763800 | 2.94547900 | -1.48300900 |
| B | -2.22158200 | 0.23059900 | -0.07412900 |
| C | 2.36030800 | -1.11549000 | -2.52665200 |
| H | 1.47682400 | -1.10399400 | -3.16134200 |
| C | -1.30477100 | 1.48123600 | -0.24723600 |
| B | -1.43151900 | 2.91268700 | 0.66071900 |
| H | -0.38941200 | 3.99032900 | -2.98385800 |
| C | -1.63424000 | -1.19017600 | -0.25562200 |
| B | 1.00646300 | 2.29015700 | -1.60384400 |
| H | 1.75081900 | 4.65951800 | -0.87036500 |
| H | -1.18719000 | 5.30375200 | -0.31109700 |
| C | 0.34311000 | 1.09949000 | -0.55994900 |
| B | -0.12464600 | 1.72832300 | 0.97855400 |
| C | -0.05962400 | -2.94633100 | -0.69729900 |
| H | 0.94301300 | -3.29059800 | -0.93364200 |
| H | 0.55371400 | 4.04042900 | 1.89621900 |
| B | -0.31197800 | 3.43530900 | -1.93861800 |
| H | 2.38989500 | 1.97381700 | 0.55092900 |
| B | -0.77361700 | 4.19382500 | -0.37950100 |
| C | -1.08415600 | -3.88041500 | -0.53543800 |
| H | -0.86341700 | -4.93831500 | -0.64812700 |
| B | -1.76706600 | 2.89584600 | -1.07576900 |
| C | -2.40167900 | -3.48515800 | -0.22775500 |
| H | -3.17552100 | -4.23910700 | -0.10877100 |
| B | 0.23788500 | 3.46281900 | 0.90991500 |
| H | 1.82589900 | 1.93845900 | -2.37892000 |
| C | -2.67523700 | -2.12821700 | -0.08796600 |
| B | 1.33994100 | 2.30494500 | 0.12986400 |
| H | -2.31116500 | 2.97699800 | 1.45061100 |
| C | -3.73055000 | 0.00507400 | 0.23887600 |
| B | 0.92918500 | 3.81988200 | -0.70463000 |
| C | -5.20787700 | -1.93710000 | 0.46170900 |
| H | -5.38151100 | -3.00971200 | 0.44737900 |
| C | -6.27409500 | -1.05939500 | 0.73076200 |
| H | -7.26338500 | -1.46553000 | 0.92242500 |
| C | -6.07860400 | 0.32204200 | 0.75395400 |
| H | -6.91402000 | 0.98335100 | 0.96296500 |
| C | -4.80371200 | 0.85646300 | 0.50677800 |
| H | -4.65702700 | 1.93255400 | 0.52511000 |
| C | 2.23206500 | -0.81651300 | -1.15402000 |
| C | 3.59598900 | -1.40005300 | -3.10850100 |
| H | 3.65963900 | -1.60986700 | -4.17223600 |
| C | 4.74161600 | -1.41624500 | -2.31134800 |
| H | 5.71043700 | -1.64713600 | -2.74476700 |
| C | 4.63442400 | -1.15611500 | -0.94639400 |
| H | 5.52058800 | -1.21100600 | -0.32090200 |
| C | 3.39702800 | -0.85659300 | -0.34784400 |
| C | 3.35372900 | -0.63108600 | 1.12467200 |
| C | 4.32654900 | 0.16512400 | 1.75712700 |
| H | 5.08720700 | 0.65507300 | 1.15651200 |
| C | 4.30370600 | 0.36637000 | 3.13688100 |
| H | 5.05664700 | 0.99776400 | 3.59997700 |
| C | 3.30870600 | -0.22738700 | 3.92031500 |
| H | 3.28978100 | -0.06838400 | 4.99443600 |
| C | 2.34118300 | -1.02648500 | 3.30907500 |
| H | 1.57023400 | -1.50417700 | 3.90707100 |
| C | 2.36604100 | -1.22850400 | 1.92636000 |
| H | 1.63072900 | -1.88633000 | 1.47437100 |

## TD-DFT calculations 3b:

### Calculated absorption spectrum

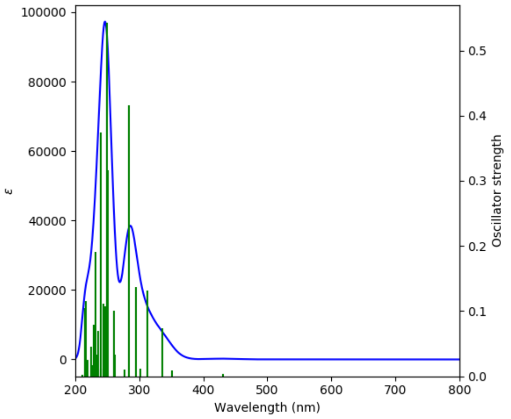


| Orbital | Energy [eV] | Symmetry |
|---|---|---|
| L+4 | 0.63 | A |
| L+3 | 0.34 | A |
| L+2 | 0.13 | A |
| L+1 | -0.70 | A |
| LUMO | -1.84 | A |
| HOMO | -7.41 | A |
| H-1 | -7.65 | A |
| H-2 | -8.15 | A |
| H-3 | -8.27 | A |
| H-4 | -8.35 | A |

### TD-DFT CAMB3LYP/6-31+G(d,p), n-Hexane

Table S11: Lowest energy singlet electronic transition of **3b** (TD-DFT CAM-B3LYP/6-31+G(d,p), *n*-Hexane).

| State | E [eV] | λ [nm] | *f* | Symmetry | Major contributions | Λ |
|---|---|---|---|---|---|---|
| 1 | 2.88 | 430.44 | 0.0031 | Singlet-A | HOMO→LUMO (91%) | 0.51 |
| 2 | 3.53 | 350.79 | 0.0091 | Singlet-A | H-2→LUMO (89%) | 0.60 |
| 3 | 3.69 | 335.95 | 0.0738 | Singlet-A | H-3→LUMO (16%), H-1→LUMO (62%) | 0.34 |
| 4 | 3.96 | 313.04 | 0.1311 | Singlet-A | H-4→LUMO (61%), HOMO→L+1 (19%) | 0.60 |
| 5 | 4.11 | 301.89 | 0.0114 | Singlet-A | H-9→LUMO (30%), H-3→LUMO (25%), H-1→LUMO (13%) | 0.50 |
| 6 | 4.21 | 294.42 | 0.1364 | Singlet-A | H-6→LUMO (17%), H-3→LUMO (12%), HOMO→L+1 (31%) | 0.54 |
| 7 | 4.37 | 283.97 | 0.4162 | Singlet-A | H-6→LUMO (32%), H-4→LUMO (14%), HOMO→L+1 (21%) | 0.62 |
| 8 | 4.48 | 276.50 | 0.0099 | Singlet-A | H-9→LUMO (30%), H-3→LUMO (22%) | 0.53 |
| 9 | 4.73 | 261.88 | 0.0332 | Singlet-A | H-11→LUMO (43%), H-5→LUMO (28%) | 0.47 |
| 10 | 4.76 | 260.65 | 0.1 | Singlet-A | H-11→LUMO (33%), H-5→LUMO (38%) | 0.45 |
| 11 | 4.93 | 251.34 | 0.3167 | Singlet-A | H-8→LUMO (13%), H-2→L+1 (31%), HOMO→L+1 (11%) | 0.59 |
| 12 | 4.98 | 249.08 | 0.5429 | Singlet-A | H-8→LUMO (10%), H-2→L+1 (39%), H-1→L+1 (12%) | 0.55 |
| 13 | 5.03 | 246.34 | 0.1075 | Singlet-A | H-1→L+1 (39%) | 0.46 |
| 14 | 5.09 | 243.47 | 0.1119 | Singlet-A | H-12→LUMO (24%), H-7→LUMO (10%), H-1→L+5 (10%) | 0.54 |
| 15 | 5.16 | 240.42 | 0.0123 | Singlet-A | H-12→LUMO (34%), H-1→L+5 (11%) | 0.51 |
| 16 | 5.16 | 240.04 | 0.3739 | Singlet-A | H-1→L+2 (62%) | 0.72 |
| 17 | 5.25 | 236.02 | 0.0694 | Singlet-A | H-5→L+2 (10%), H-3→L+2 (15%) | 0.53 |
| 18 | 5.29 | 234.53 | 0.0334 | Singlet-A | H-10→LUMO (25%), H-9→LUMO (12%), H-8→LUMO (31%) | 0.70 |
| 19 | 5.37 | 231.05 | 0.1904 | Singlet-A | H-6→LUMO (12%), H-4→L+1 (22%), HOMO→L+3 (11%) | 0.60 |
| 20 | 5.42 | 228.71 | 0.0785 | Singlet-A | H-9→L+1 (12%), H-7→LUMO (40%), H-3→L+1 (11%) | 0.43 |
| 21 | 5.46 | 227.27 | 0.0174 | Singlet-A | H-13→LUMO (18%), H-9→L+1 (14%), H-1→L+1 (11%) | 0.51 |
| 22 | 5.52 | 224.48 | 0.0452 | Singlet-A | H-10→LUMO (11%), H-4→L+1 (39%), HOMO→L+3 (24%) | 0.58 |
| 23 | 5.65 | 219.41 | 0.0255 | Singlet-A | H-13→LUMO (32%), H-3→L+1 (37%) | 0.41 |
| 24 | 5.71 | 217.24 | 0.1148 | Singlet-A | H-13→LUMO (12%), H-6→L+1 (43%), H-5→L+1 (12%) | 0.60 |
| 25 | 5.76 | 215.40 | 0.1041 | Singlet-A | H-10→LUMO (13%), H-8→L+1 (11%), HOMO→L+11 (16%) | 64 |

**Orbitals relevant to the $S_1 \leftarrow S_o$ and $S_2 \leftarrow S_o$ transitions**

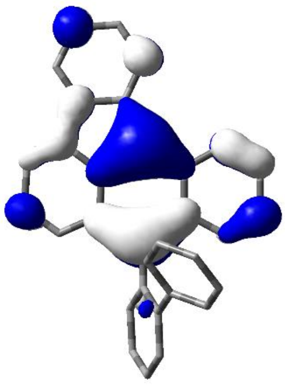

**LUMO:** -1.84 **eV**

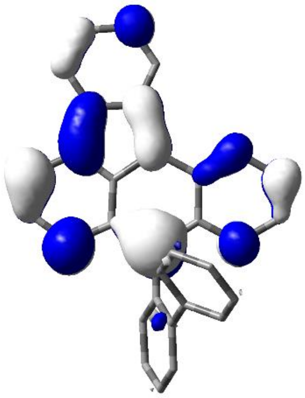

**LUMO+1:** -0.70**eV**

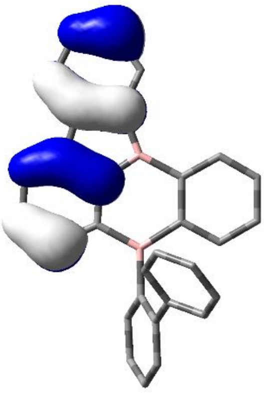

**HOMO:** -7.41 **eV**

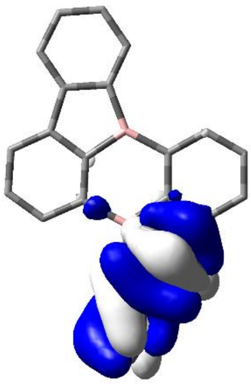

**HOMO-1:** -7.65 **eV**

Isovalue= 0.03

| | | | |
|---|---|---|---|
| C | 4.01006900 | 0.68943100 | 0.36383000 |
| C | 0.43005200 | 1.52432700 | -0.11335700 |
| B | -0.72385700 | 0.59121100 | -0.62791500 |
| B | 2.07660800 | -0.50699000 | -0.46751900 |
| C | -2.42304500 | 2.16556000 | -1.68631600 |
| H | -1.58168500 | 2.55512800 | -2.25524300 |
| C | 1.69948800 | 0.94017200 | -0.05059200 |
| C | 0.32325100 | 2.86154600 | 0.33657100 |
| H | -0.63747700 | 3.36955700 | 0.32423400 |
| C | 1.45075300 | 3.54495100 | 0.80503200 |
| H | 1.34787400 | 4.57312300 | 1.14219000 |
| C | 2.71901500 | 2.93337800 | 0.85787100 |
| H | 3.57148600 | 3.49494100 | 1.23237100 |
| C | 2.83832900 | 1.61316500 | 0.42747400 |
| C | 3.61951900 | -0.58611600 | -0.15761500 |
| C | 5.32570300 | 0.94015000 | 0.73557400 |
| H | 5.62167500 | 1.90858600 | 1.13050600 |
| C | 6.28265100 | -0.07982200 | 0.59657600 |
| H | 7.31294900 | 0.10909800 | 0.88568100 |
| C | 5.92283400 | -1.33020600 | 0.09283600 |
| H | 6.67255900 | -2.10962700 | -0.00831600 |
| C | 4.59264400 | -1.58016200 | -0.28289800 |
| H | 4.33068700 | -2.56049800 | -0.67231200 |
| C | -2.19730000 | 1.14785000 | -0.73612400 |
| C | -3.69872200 | 2.67110800 | -1.94304400 |
| H | -3.83693100 | 3.44418000 | -2.69375600 |
| C | -4.78977800 | 2.18316900 | -1.22083000 |
| H | -5.78660100 | 2.57807500 | -1.39582500 |
| C | -4.59084600 | 1.19922100 | -0.25299100 |
| H | -5.43396400 | 0.85026700 | 0.33672200 |
| C | -3.31256700 | 0.67067100 | 0.00095000 |
| C | -3.15444000 | -0.35827900 | 1.06633300 |
| C | -4.06195200 | -1.42723200 | 1.17941700 |
| H | -4.87238300 | -1.51724400 | 0.46154200 |
| C | -3.91702800 | -2.39027000 | 2.17887500 |
| H | -4.62354200 | -3.21351900 | 2.23877300 |
| C | -2.86164800 | -2.30496500 | 3.09225100 |
| H | -2.74799700 | -3.05520400 | 3.86932900 |
| C | -1.95637400 | -1.24578000 | 2.99744000 |
| H | -1.13822800 | -1.16167600 | 3.70713800 |
| C | -2.10379200 | -0.28187900 | 1.99735400 |
| H | -1.41305600 | 0.55524000 | 1.95850200 |
| C | 1.20746100 | -2.71651800 | -1.52831800 |
| C | 0.96756400 | -1.41801900 | -1.04798300 |
| C | -0.36214800 | -0.87388400 | -1.12798300 |
| C | -1.36286800 | -1.67178400 | -1.70884200 |
| C | -1.09920700 | -2.96147600 | -2.18536800 |
| C | 0.18806400 | -3.48987800 | -2.09209700 |
| H | 2.21008000 | -3.13093600 | -1.46776500 |
| H | -2.37211100 | -1.28086800 | -1.79502800 |
| H | -1.89946500 | -3.54926900 | -2.62715700 |
| H | 0.39830400 | -4.49103400 | -2.45857400 |

## References


1. C. J. Berger, G. He, C. Merten, R. McDonald, M. J. Ferguson and E. Rivard, *Inorg. Chem.*, 2014, **53**, 1475-1486.
2. D. Kaufmann, *Chem. Ber.*, 1987, **120**, 901–905.
3. R. N. Grimes, *Carboranes*, Elsevier, London, UK, 2nd edn., 2011.
4. B. Wrackmeyer, E. V. Klimkina and W. Milius, *Appl. Organometal. Chem.*, 2010, **24**, 25-32.
5. G. M. Sheldrick, *Acta Crystallogr.*, 2015, **A71**, 3–8.
6. G. M. Sheldrick, *Acta Crystallogr.*, 2008, **A64**, 112–122.
7. C. B. Hübschle, G. M. Sheldrick and B. Dittrich, *J. Appl. Crystallogr.*, 2011, **44**, 1281–1284.
8. A. L. Spek, *Acta Crystallogr.* , 2015, **C71**, 9–18.
9. H. P. K. Brandenburg, Diamond Version 4.2.0. Crystal and M. S. Visualization, *Diamond, 4.2.0.*, 2016.
10. J.-D. Chai and M. Head-Gordon, *Phys. Chem. Chem. Phys.*, 2008, **10**, 6615–6620.
11. Y. Zhao and D. G. Truhlar, *Theor. Chem. Acc.*, 2007, **120**, 215–241.
12. Y. Zhao and D. G. Truhlar, *J. Chem. Theory. Comput.*, 2008, **4**, 1849–1868.
13. N. Mardirossian and M. Head-Gordon, *J. Chem. Theory. Comput.*, 2016, **12**, 4303–4325.
14. W. J. Hehre, R. Ditchfield and J. A. Pople, *J. Chem. Phys.*, 1972, **56**, 2257–2261.
15. P. C. Hariharan and J. A. Pople, *Theor. Chim. Acta*, 1973, **28**, 213–222.
16. K. Fukui, *Acc. Chem. Res.*, 1981, **14**, 363–368.
17. M. J. Frisch, G. W. Trucks, H. B. Schlegel, G. E. Scuseria, M. A. Robb, J. R. Cheeseman, G. Scalmani, V. Barone, B. Mennucci, G. A. Petersson, H. Nakatsuji, M. Caricato, X. Li, H. P. Hratchian, A. F. Izmaylov, J. Bloino, G. Zheng, J. L. Sonnenberg, M. Hada, M. Ehara, K. Toyota, R. Fukuda, J. Hasegawa, M. Ishida, T. Nakajima, Y. Honda, O. Kitao, H. Nakai, T. Vreven, J. A. Montgomery, Jr.; , J. E. Peralta, F. Ogliaro, M. Bearpark, J. J. Heyd, E. Brothers, K. N. Kudin, V. N. Staroverov, R. Kobayashi, J. Normand, K. Raghavachari, A. Rendell, J. C. Burant, S. S. Iyengar, J. Tomasi, M. Cossi, N. Rega, J. M. Millam, M. Klene, J. E. Knox, J. B. Cross, V. Bakken, C. Adamo, J. Jaramillo, R. Gomperts, R. E. Stratmann, O. Yazyev, A. J. Austin, R. Cammi, C. Pomelli, J. W. Ochterski, R. L. Martin, K. Morokuma, V. G. Zakrzewski, G. A. Voth, P. Salvador, J. J. Dannenberg, S. Dapprich, A. D. Daniels, Ö. Farkas, J. B. Foresman, J. V. Ortiz, J. Cioslowski and D. J. Fox, *Journal*, 2009.
18. M. J. Frisch, G. W. Trucks, H. B. Schlegel, G. E. Scuseria, M. A. Robb, J. R. Cheeseman, G. Scalmani, V. Barone, B. Mennucci, G. A. Petersson, H. Nakatsuji, M. Caricato, X. Li, H. P. Hratchian, A. F. Izmaylov, J. Bloino, G. Zheng, J. L. Sonnenberg, M. Hada, M. Ehara, K. Toyota, R. Fukuda, J. Hasegawa, M. Ishida, T. Nakajima, Y. Honda, O. Kitao, H. Nakai, T. Vreven, J. A. Montgomery, Jr.; , J. E. Peralta, F. Ogliaro, M. Bearpark, J. J. Heyd, E. Brothers, K. N. Kudin, V. N. Staroverov, R. Kobayashi, J. Normand, K. Raghavachari, A. Rendell, J. C. Burant, S. S. Iyengar, J. Tomasi, M. Cossi, N. Rega, J. M. Millam, M. Klene, J. E. Knox, J. B. Cross, V. Bakken, C. Adamo, J. Jaramillo, R. Gomperts, R. E. Stratmann, O. Yazyev, A. J. Austin, R. Cammi, C. Pomelli, J. W. Ochterski, R. L. Martin, K. Morokuma, V. G. Zakrzewski, G. A. Voth, P. Salvador, J. J. Dannenberg, S. Dapprich, A. D. Daniels, Ö. Farkas, J. B. Foresman, J. V. Ortiz, J. Cioslowski and D. J. Fox, *Journal*, 2016.
19. T. Lu and F. Chen, *J. Comput. Chem.*, 2012, **33**, 580–592.
20. C. Lee, W. Yang and R. G. Parr, *Phys. Rev. B*, 1988, **37**, 785–789.
21. G. A. Petersson and M. A. Al-Laham, *J. Chem. Phys.*, 1991, **94**, 6081–6090.
22. G. A. Petersson, A. Bennett, T. G. Tensfeldt, M. A. Al-Laham, W. A. Shirley and J. Mantzaris, *J. Chem. Phys.*, 1988, **89**, 2193–2218.
23. M. J. Peach, P. Benfield, T. Helgaker and D. J. Tozer, *J. Chem. Phys.*, 2008, **128**, 044118.
24. T. Yanai, D. P. Tew and N. C. Handy, *Chem. Phys. Lett.*, 2004, **393**, 51–57.
25. C. Ohrenberg, P. Ge, P. Schebler, C. G. Riordan, G. P. A. Yap and A. L. Rheingold, *Inorg. Chem.*, 1996, **35**, 749-754.
